# Epigenetic modulation through BET bromodomain inhibitors as a novel therapeutic strategy for progranulin-deficient frontotemporal dementia

**DOI:** 10.1038/s41598-024-59110-7

**Published:** 2024-04-20

**Authors:** Zachary C. Rosenthal, Daniel M. Fass, N. Connor Payne, Angela She, Debasis Patnaik, Krista M. Hennig, Rachel Tesla, Gordon C. Werthmann, Charlotte Guhl, Surya A. Reis, Xiaoyu Wang, Yueting Chen, Michael Placzek, Noelle S. Williams, Jacob Hooker, Joachim Herz, Ralph Mazitschek, Stephen J. Haggarty

**Affiliations:** 1 Chemical Neurobiology Laboratory, Precision Therapeutics Unit, Center for Genomic Medicine, Departments of Neurology and Psychiatry, Harvard Medical School, Massachusetts General Hospital, Boston, MA USA; 2 Department of Chemistry & Chemical Biology, Harvard University, Cambridge, MA USA; 3 Center for Systems Biology, Massachusetts General Hospital, Boston, MA USA; 4 Harvard T.H. Chan School of Public Health, Boston, MA USA; 5 Department of Molecular Genetics, University of Texas Southwestern Medical Center, Dallas, TX USA; 6 Center for Translational Neurodegeneration Research, University of Texas Southwestern Medical Center, Dallas, TX USA; 7 Faculty of Chemistry and Earth Sciences, Institute of Organic Chemistry and Macromolecular Chemistry, Friedrich Schiller University Jena, Jena, Germany; 8 Department of Biochemistry, University of Texas Southwestern Medical Center, Dallas, TX USA; 9 Department of Radiology, Harvard Medical School, Boston, MA USA; 10 Athinoula A. Martinos Center for Biomedical Imaging, Massachusetts General Hospital, Boston, MA USA; 11 Department of Neuroscience, University of Texas Southwestern Medical Center, Dallas, TX USA; 12 Department of Neurology and Neurotherapeutics, University of Texas Southwestern Medical Center, Dallas, TX USA; 13 Broad Institute of MIT and Harvard, Cambridge, MA USA

**Keywords:** Drug discovery, Drug screening, Medicinal chemistry, Target identification, Target validation, Chemical biology, Chemical genetics, Chemical tools, Mechanism of action, Small molecules, Target identification, Target validation, Neuroscience, Epigenetics in the nervous system, Neural ageing, Stem cells in the nervous system, Stem cells, Neural stem cells, Pluripotent stem cells

## Abstract

Frontotemporal dementia (FTD) is a debilitating neurodegenerative disorder with currently no disease-modifying treatment options available. Mutations in *GRN* are one of the most common genetic causes of FTD, near ubiquitously resulting in progranulin (PGRN) haploinsufficiency. Small molecules that can restore PGRN protein to healthy levels in individuals bearing a heterozygous *GRN* mutation may thus have therapeutic value. Here, we show that epigenetic modulation through bromodomain and extra-terminal domain (BET) inhibitors (BETi) potently enhance PGRN protein levels, both intracellularly and secreted forms, in human central nervous system (CNS)-relevant cell types, including in microglia-like cells. In terms of potential for disease modification, we show BETi treatment effectively restores PGRN levels in neural cells with a *GRN* mutation known to cause PGRN haploinsufficiency and FTD. We demonstrate that BETi can rapidly and durably enhance PGRN in neural progenitor cells (NPCs) in a manner dependent upon BET protein expression, suggesting a gain-of-function mechanism. We further describe a CNS-optimized BETi chemotype that potently engages endogenous BRD4 and enhances PGRN expression in neuronal cells. Our results reveal a new epigenetic target for treating PGRN-deficient forms of FTD and provide mechanistic insight to aid in translating this discovery into therapeutics.

## Introduction

The selective degeneration of the frontal and temporal lobes characterizes frontotemporal dementia (FTD). It is the second most common cause of presenile dementia, accounting for 5–15% of all dementia cases^1^. Clinically, FTD patients commonly present with severe personality and behavioral changes, as well as fluent or non-fluent aphasias^2^. Pathologically, FTD patients show evidence of abnormal intracellular protein aggregation, with the specific compositions of aggregating proteins distinguishing FTD subtypes^1^.

One of the most significant genetic risk factors for FTD is mutations in the *GRN* gene, which encodes the progranulin (PGRN) protein (FTD-*GRN*). *GRN* mutation frequency in FTD is estimated to be between 1 and 11%, with a large range due to significant differences in mutation frequency among various populations^3,4^. By age sixty, over half of *GRN* mutation carriers are affected by FTD; by age seventy, over 90% are affected^5^. To date, at least seventy pathogenic mutations in *GRN* have been identified, with the majority introducing a premature stop codon^3^. Almost all of these mutations reduce *GRN* mRNA and corresponding PGRN protein, leading to PGRN haploinsufficiency.

PGRN is a highly glycosylated, 88 kDa protein, which is secreted and cleaved either extracellularly or after re-internalization into lysosomes to 6 kDa fragments (granulins) via the actions of several proteases^3,6,7^. PGRN modulates various biological processes, such as mediating wound repair and inflammation, promoting epithelial cell growth, regulating lysosomal homeostasis, and inhibiting transcriptional elongation^8–10^. PGRN deficiency causes broad lysosomal dysfunction characterized by inclusion-filled lysosomes and increased expression of lysosomal proteins such as cathepsin D and LAMP1. In contrast, homozygous loss of function mutations in *GRN* causes the severe lysosomal storage disorder neuronal ceroid lipofuscinosis^11,12^. Furthermore, PGRN haploinsufficiency causes aberrant accumulation of cytosolic ubiquitin and TAR DNA-binding protein (TDP-43)-positive inclusions—pathological hallmarks of FTD-*GRN*^13^.

A possible therapeutic mechanism for treating FTD-*GRN* is to increase the expression of PGRN from the remaining wild-type allele. In support of this therapeutic hypothesis, enhancing PGRN protein levels has shown benefit in several animal models^14–16^. Most recently, it was demonstrated that delivering PGRN to the brains of *Grn*^-/-^ mice via an engineered protein transport vehicle (PTV) fusion protein rescued several pathological phenotypes, including restoring lipid homeostasis, reducing gliosis, and preventing neurodegeneration^16^ However, an essential requirement for therapeutic approaches that upregulate PGRN may be long-term, chronic dosing before the emergence of symptoms. Thus, from a therapeutic perspective, approaches that enhance PGRN via genetic methods or through intravenous administration of recombinant PGRN may have limitations.

Identifying novel small molecules that can enhance PGRN protein levels has been the focus of many drug development efforts, including our own^17,18^. As a proof of concept for this approach, previous works have identified histone deacetylase (HDAC) inhibitors as enhancers of PGRN^17–19^. However, current clinical HDAC inhibitors have several hematologic, cardiac, and metabolic side effects that may prove limiting outside of an oncology context^20^. We thus sought to identify novel classes of epigenetic regulators with more acceptable safety profiles for long-term dosing in both symptomatic and pre-symptomatic FTD-*GRN* populations.

This manuscript describes the discovery and mechanistic characterization of bromodomain and extra-terminal domain (BET) inhibitors (BETi) as enhancers of *GRN* mRNA and protein levels in human neural progenitor cells and post-mitotic neurons. We showed that BETi can rescue PGRN expression in PGRN haploinsufficient human iPSC-derived NPCs and neurons, as well as a microglia-like cell line. We further investigated the selectivity, kinetic, and protein dependency requirements of BET inhibitors. We showed that BET inhibitors operate to enhance PGRN in NPCs through a unique gain-of-function mechanism. Finally, we synthesized a family of novel BET inhibitors with properties explicitly optimized for CNS drug discovery. We demonstrated that these compounds could potently engage lysate-derived BRD4 and enhance PGRN in NPCs, providing new tools to investigate the neuroepigenetic regulation of *GRN* expression and leads for novel therapeutic development.

## Results

### Bromodomain inhibitors enhance cellular PGRN levels

Previous work has characterized the ability of HDAC inhibitors possessing varying selectivity and kinetic profiles to enhance PGRN protein levels both in mouse primary neurons and Neuro-2A cells, as well as in human NPCs and neurons^17–19,21^. However, while HDAC inhibitors have seen common clinical use in neoplastic diseases, their tolerability and safety profiles are not ideal for chronic dosing in a pre-symptomatic population^20,22^. Motivated by the knowledge that targeting epigenetic regulators can modulate PGRN expression, we sought to discover novel classes of small-molecule enhancers of cellular PGRN.

**Figure 1:**
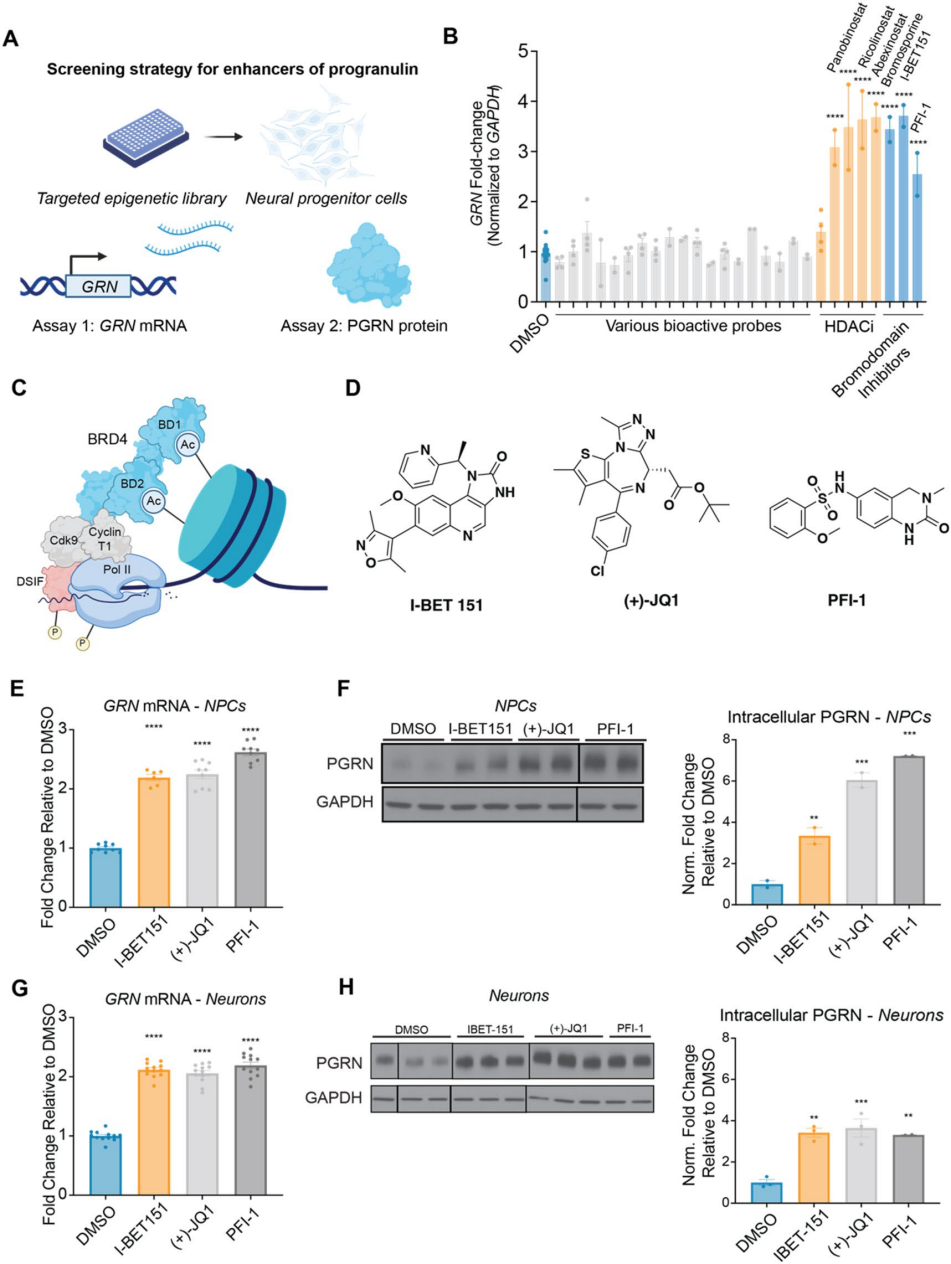
Bromodomain inhibitors enhance cellular PGRN in human neural progenitor cells. (**A**) Assay strategy for identifying novel small-molecule enhancers of PGRN. Assay 1: qPCR to identify *GRN* enhancers. Assay 2: Immunoblotting to validate PGRN enhancement at the protein level. (**B**) Bromodomain inhibitors (10 µM) enhance *GRN* mRNA in human NPCs after 24 h. Data are expressed as mean ± S.E. of *n* = 2–4 compound treatment biological replicates or *n* = *15* DMSO biological replicates normalized to *GAPDH* and shown relative to DMSO. Compound concentrations used: bromosporine, 10 µM; I-BET151, 10 µM; PFI-1, 10 µM. (**C**) Schematic representation of human BRD4 with selected domains and interactors^32^. (**D**) Structures of BET inhibitor probes used for further investigations. (**E**–**F**) BET inhibitors enhance *GRN* mRNA and protein levels in NPCs after 24 h of treatment. (**G**-**H**) BET inhibitors enhance PGRN mRNA levels and protein levels in 18-day differentiated neurons after 24 h of treatment. Solid lines on blots indicate discontinuity. Significance determined by Dunnett’s Multiple Comparison, *p < 0.05, **p < 0.01, ***p < 0.001, ****p < 0.0001.

To assess the regulation of PGRN in human, CNS-relevant cell types, we employed an expandable NPC line (8330–8 RC1) generated through an iPSC intermediate from reprogrammed human fibroblasts (previously described)^23–27^. We first screened for compounds that could enhance *GRN* mRNA and followed up on hits by evaluating PGRN protein levels (Fig. 1A). We performed an initial screen for enhancers of *GRN* mRNA in NPCs, using a library of bioactive probes that were selected for their ability to target diverse epigenetic regulators and signaling pathways related to neuroplasticity (Fig. 1B, Supplemental Table 1). As part of this screen, we included the previously described HDAC inhibitor positive controls panobinostat (1 µM), abexinostat (1 µM), and ricolinostat (10 µM) (Fig. 1B)^18^.

**Table 1:** Calculated K_D,app_ values (with 95% confidence interval) for A and B-series compounds against HEK-293 T and NPC-derived BRD4, as well as the individual bromodomains of BRD4.

	Endogenous BRD4 (HEK293T), *K*_D,app_ (nM)	Endogenous BRD4 (NPC), *K*_D,app_ (nM)	CNS MPO Score
18A	44 (40, 49)	46 (39, 53)	5.0
81A	30 (28, 32)	32 (29, 36)	5.0
82A	49 (45, 53)	45 (40, 50)	4.8
84A	370 (325, 422)	378 (316, 453)	4.2
85A	93 (84, 102)	100 (93, 108)	4.2
86A	559 (505, 620)	559 (482, 649)	4.6
18B	121 (110, 134)	131 (119, 144)	5.2
81B	538 (458, 632)	532 (439, 643)	5.2
82B	519 (479, 563)	430 (357, 517)	5.0
84B	196 (175, 220)	195 (170, 222)	4.6
85B	3909 (3418, 4529)	3813 (2938, 5316)	4.7
86B	7154 (5735, 10,001)	5900 (3734, 16,424)	4.8

Strikingly, from our panel of bioactive probes, we identified three bromodomain inhibitors—bromosporine (10 µM), I-BET151 (10 µM), and PFI-1 (10 µM)—as active enhancers of *GRN* mRNA (Fig. 1B)^28–30^. Bromodomains are modular protein domains that bind to acetylated lysine residues on histones and other proteins^31^. BET family members are a subset of bromodomain-containing proteins and include the related proteins BRD2, BRD3, BRD4, and BRDT, which each possess two bromodomains, BD1 and BD2^31^. Through the Extra-Terminal (ET) domain, BET family members can interact with various transcriptional regulators, including CHD4, ATAD5, and JMJD6^32^. BRD4, in particular, interacts with the Mediator complex, which has been found preferentially in ‘super-enhancer’ regions and associated with active transcriptional regions across various cell types^33–35^. BRD4 also directs CDK9 and Cyclin T1, collectively constituting the Positive Transcription Elongation Factor B (P-TEFb), to primary response genes to promote productive transcriptional elongation by RNA polymerase II (Fig. 1C)^36–39^.

Identifying BET-selective probes I-BET151 and PFI-1 as enhancers of *GRN* mRNA prompted us to investigate the BET family of bromodomain-containing proteins further. For further screening, we utilized the BET-selective probe compounds I-BET151, PFI-1, and ( +)-JQ1 due to their selectivity for BET family members, while bromosporine promiscuously targets bromodomains (Fig. 1D)^29,30,40,41^. We further found that in NPCs, PFI-1 (10 µM), I-BET151 (2.5 µM), and ( +)-JQ1 (1 µM) increased *GRN* mRNA by ~ 2.5-fold and substantially increased PGRN protein levels (Fig. 1E, F, Supplemental Fig. 1). We next asked whether this effect could be recapitulated in growth-factor differentiated neurons. Indeed, we found that BET inhibitors could enhance both *GRN* mRNA and PGRN protein levels in neurons (Fig. 1G, H, Supplemental Fig. 2). Furthermore, we next asked whether BET inhibitors could enhance extracellular PGRN levels in NPC supernatants. We found that I-BET151 (10 µM), ( +)-JQ1 (1 µM) and PFI-1 (10 µM) were all able to enhance extracellular PGRN levels by 30–70% (Supplemental Fig. 3). Finally, we found that ( +)-JQ1 (1 µM), but not the inactive enantiomer (-)-JQ1 (1 µM), enhanced *GRN* mRNA and PGRN protein levels in NPCs, supporting specificity for BET family members (Supplemental Fig. 4)^40^. Collectively, these results demonstrate the potential of targeting BET family members to enhance PGRN in CNS-relevant cell types.

### BET inhibitors can enhance lysosomal genes and rescue loss of PGRN in NPCs and iPSC-derived neurons bearing the GRN R493X mutation

**Figure 2:**
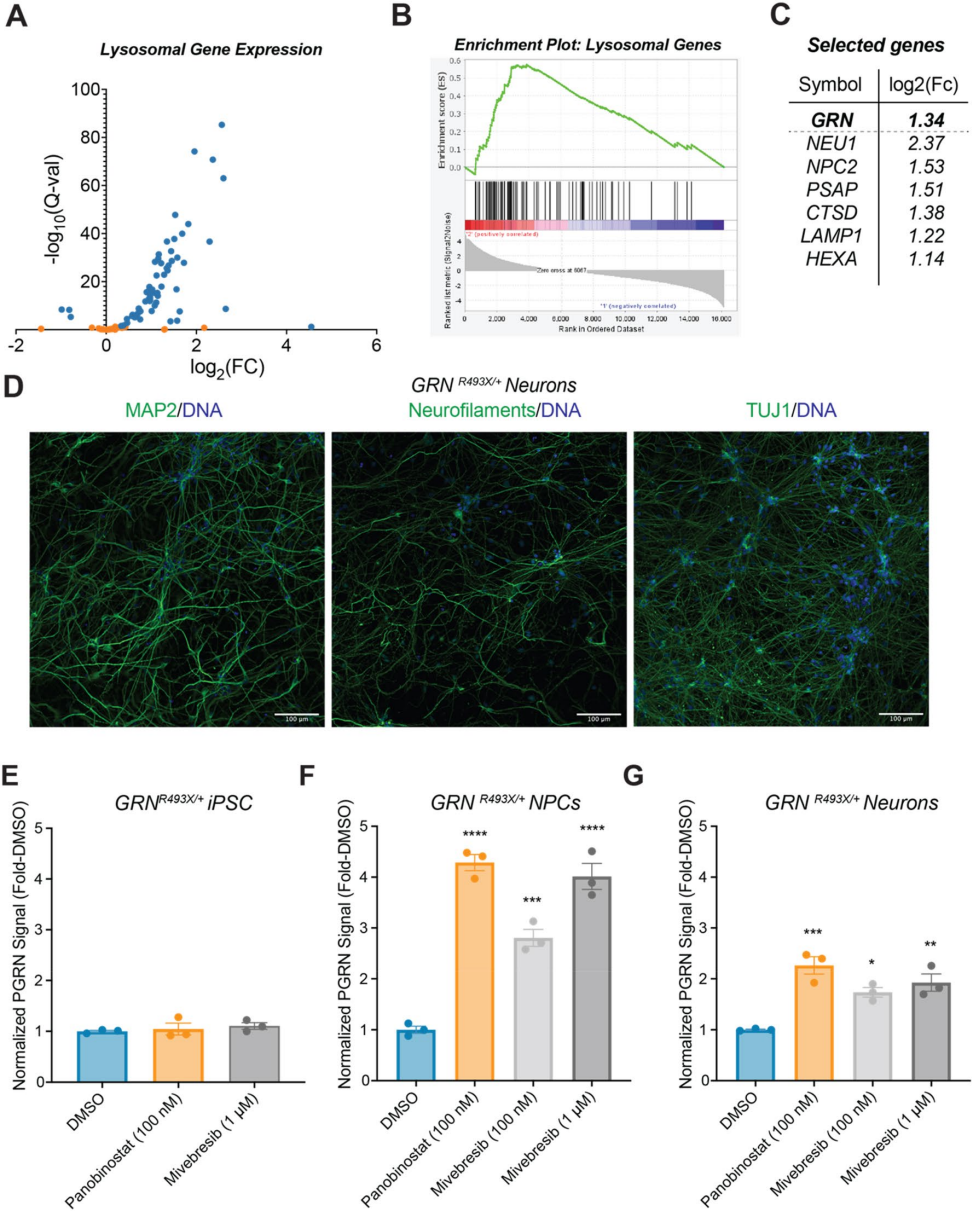
BET inhibitors can enhance lysosomal gene expression and rescue PGRN in *GRN*-haploinsufficient NPCs and neurons. (**A**) RNA-seq data of human dermal fibroblasts treated with 1 µM mivebresib for 24 h displaying only lysosomal genes (list from Sardiello et al^42^., Table S1). Significantly altered genes are marked in blue and non-significant changes are marked in orange. Data shown as − log_10_(Q-value) versus log_2_(fold-change) of mivebresib treatment compared to DMSO, and represent the average of three biological replicates. (**B**) Gene Set Enrichment Analysis conducted using lysosomal gene list^42–44^. (**C**) Fold-change of *GRN* as well as selected lysosomal genes shown in (**A**). (**D**) Immunocytochemistry of fixed, 2-week growth factor differentiated *GRN*^*R493*^^*X/*+^ neurons demonstrating MAP2, neurofilaments (SMI-312R), and TUJ1 expression. (**E**–**G**) 24 h treatment with mivebresib or panobinostat rescues PGRN haploinsufficiency in *GRN-*haploinsufficient NPCs and neurons, but not in undifferentiated iPSCs. Significance determined by Dunnett’s Multiple Comparison, *p < 0.05, **p < 0.01, ***p < 0.001, ****p < 0.0001.

We next profiled the transcriptome-wide effects of BET inhibition. We treated non-immortalized human dermal fibroblasts with 1 µM mivebresib, a potent, clinical-stage pan-BET inhibitor, for 24 h and evaluated gene expression changes via RNA sequencing (Supplemental Fig. 5). We found that BET inhibition led to broad transcriptomic alterations, consistent with the roles of BET family members as transcriptional regulators (Supplemental Fig. 5). We next analyzed gene expression changes in a pre-defined list of lysosomal genes and found that the majority (68/89) were significantly upregulated (Fig. 2A, Supplemental Table 2). To assess significance, we performed a gene set enrichment analysis (GSEA) and found an enrichment of upregulated lysosomal genes (FDR q-value < 0.05, Fig. 2B)^42–44^. We next asked whether this list of 68 upregulated lysosomal genes was enriched for transcription factor associations. We queried this list using ChEA3 against a literature-curated ChIP-seq library, and found an enrichment for transcription factors interferon regulatory factor 8 (IRF8) (FDR: 0.0023) and transcription factor EB (TFEB) (FDR: 0.0023), the master regulator of lysosomal biogenesis (Supplemental Table 3)^42,45^. Finally, in addition to *GRN*, we found upregulation of other lysosomal genes frequently dysregulated in lysosomal storage disorders such as *NEU1*, *NPC2, PSAP, CTSD, LAMP1,* and *HEXA* (Fig. 2C). These data suggest that BET inhibition enhances a gene network regulating lysosomal function.

We next asked whether BET inhibitors could rescue PGRN expression in cell lines bearing *GRN* mutations relevant to human disease. To this end, we derived a suite of disease-relevant cell lines from an iPSC line heterozygous for one of the most common PGRN haploinsufficiency mutations in humans, *GRN* R493X (*GRN*^*R493*^^*X/*+^), developed as part of the NIH iPSC Neurodegenerative Disease Institute^46,47^. From this line, we generated NPCs by directing the differentiation of iPSCs into neural rosettes and FACS sorting for CD133^+^/CD184^+^/CD271^−^ cells^26^. We then differentiated these NPCs into MAP2^+^/SMI-312R^+^/TUJ1^+^ neurons by withdrawing mitogenic factors (Fig. 2D)^27,48–50^.

To assess whether BET inhibitors can rescue PGRN expression in *GRN*^*R493*^^*X/*+^ iPSCs, NPCs, and neurons, we utilized a quantitative capillary gel electrophoresis system (ProteinSimple Jess). We first demonstrated the ability of our PGRN antibody to linearly quantify increases in full-length PGRN across a variation in lysate concentration from microglia-like HMC3 cells (0.125–1 mg/mL) (Supplemental Fig. 6). We next demonstrated the specificity of this detection method by showing a ~ 50% reduction in signal in a human iPSC line bearing a heterozygous *GRN* R493X mutation and a complete loss of signal in a human iPSC line bearing a homozygous *GRN* R493X mutation (Supplemental Fig. 6).

We treated *GRN*^*R493*^^*X/*+^ iPSCs, NPCs, and 14-day growth factor differentiated neurons with mivebresib or positive control panobinostat for 24 h (Fig. 2E–G) and found that mivebresib significantly increased PGRN protein levels in *GRN*^*R493X/*+^ NPCs and neurons but not in iPSCs^51^. We speculate that differences in chromatin state between iPSCs, NPCs, and neurons may result in differential responsiveness to compounds targeting epigenetic regulators. Alternatively, it could be due to differential expression of the epigenetic regulators themselves, or differences in their interaction partners between cell types. Taken together, these data demonstrate the ability of BET inhibitors to rescue PGRN haploinsufficiency in NPCs and neurons bearing a patient-relevant *GRN* haploinsufficiency mutation.

### BET and HDAC inhibitors can enhance PGRN expression in microglia-like cells

We next asked whether BET inhibition could enhance PGRN in microglia. Recent studies have increasingly recognized microglia as important mediators in the pathogenesis of FTLD^52–54^. For example, in the frontal cortex of FTLD-*GRN* mutation carriers, levels of activated ionized calcium binding adapter molecule 1 (IBA1)-positive microglia are higher than in healthy controls^53^. Moreover, microglia have the most significant transcriptional perturbations upon loss of *Grn*, and at both the mRNA and protein levels microglia express higher amounts of PGRN when compared to astrocytes and neurons^55–57^. Compounds that enhance PGRN expression in microglia may thus offer a promising therapeutic strategy for the treatment of FTD-*GRN*.

**Figure 3:**
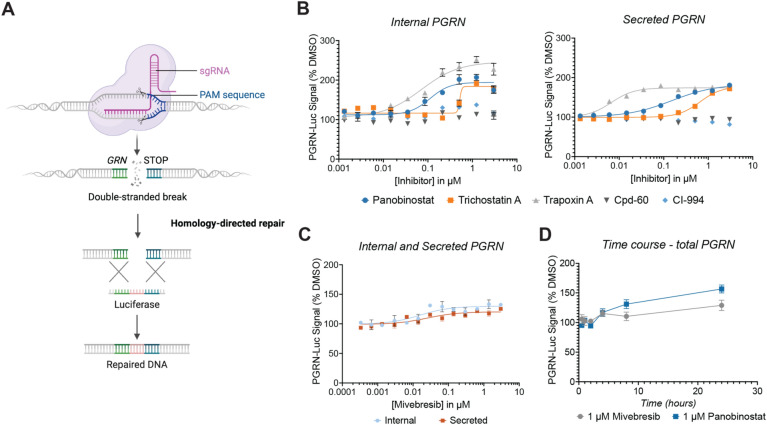
BET and HDAC inhibitors can enhance PGRN in the HMC3 PGRN-luciferase reporter line. (**A**) Schematic for CRISPR-knock in of luciferase at endogenous *GRN* locus. Adapted from “CRISPR/Cas9 Gene Editing,” by BioRender.com (2024). (**B**) 20 h treatment with various histone deacetylase inhibitors enhances both internal and secreted PGRN-Luc signal. Data are expressed as mean ± S.E. of 3 treatment biological replicates and 68 DMSO replicates. (**C**) 18 h treatment with mivebresib enhances both internal and secreted PGRN-Luc signal. Data are expressed as mean ± S.E. of 3 treatment biological replicates and 31 DMSO replicates. (**D**) Total PGRN-Luc signal after treatment with mivebresib or panobinostat treatment for indicated timeframes. Data are expressed as mean ± S.E. of 2 biological replicates.

To address this question, we utilized an immortalized, embryonic microglia-like human cell line, HMC-3 cells, which recapitulate many aspects of primary microglia^58^. We used CRISPR/Cas9-mediated knock-in (CRISPR-KI) to endogenously tag the *GRN* allele with luciferase, generating a PGRN-luciferase (PGRN-Luc) reporter line (Fig. 3A). We posited that this strategy would offer clear advantages over synthetic reporter-gene constructs, such as a luciferase governed by a synthetic *GRN* promoter, as artificial systems might not precisely replicate the chromatin arrangement of the native *GRN* locus. Moreover, as PGRN is a secreted protein, utilizing a tagged fusion protein enables separate readouts of extracellular and intracellular luciferase activity. Thus, this line allows us to also identify modulators of PGRN localization, which would not be possible with an approach that solely reports on *GRN* mRNA expression*.*

After validation of our reporter line utilizing PCR with insert-specific primers and DNA sequence confirmation, we first asked whether HDAC inhibitors could enhance PGRN in this microglia-like cell type (Supplemental Fig. 7). Previous work has characterized hydroxamic acid, ortho-amino anilide, and macrocyclic HDAC inhibitors with varying selectivity and kinetic profiles to enhance PGRN in both NPCs and in neurons^17–19^. These studies reported that hydroxamic acid-based and macrocyclic HDAC inhibitors successfully increase PGRN in NPCs and neurons, whereas ortho-amino anilides are unable to do so. We found that the hydroxamic acids panobinostat and trichostatin A, as well as the macrocyclic inhibitor trapoxin A, were able to enhance both internal and secreted PGRN at sub-µM concentrations (Fig. 3B). However, similar to our previous findings, the ortho-amino anilides Cpd-60 and CI-994 were either weakly or entirely unable to enhance secreted PGRN, suggesting shared mechanisms of HDAC regulation of *GRN* between microglia and neuronal cell types (Fig. 3B).

We next asked whether mivebresib could enhance PGRN in HMC3 PGRN-Luc cells. We found that mivebresib enhances both internal and secreted PGRN at sub-µM concentrations, to a milder extent (20–30%) than do HDAC inhibitors (80–150%) (Fig. 3C). Finally, we found that mivebresib enhanced total (combined internal and secreted) PGRN-Luc signal to the same extent as did panobinostat after 8 h of treatment, with a magnitude of an effect that levels out more rapidly over time than panobinostat (Fig. 3D). These results demonstrate that both HDAC inhibitors and BET inhibitors can enhance PGRN in a microglia-like context, with BET inhibitors producing a milder response.

### BET inhibitors depend on BET family members for PGRN induction and act rapidly and durably

**Figure 4:**
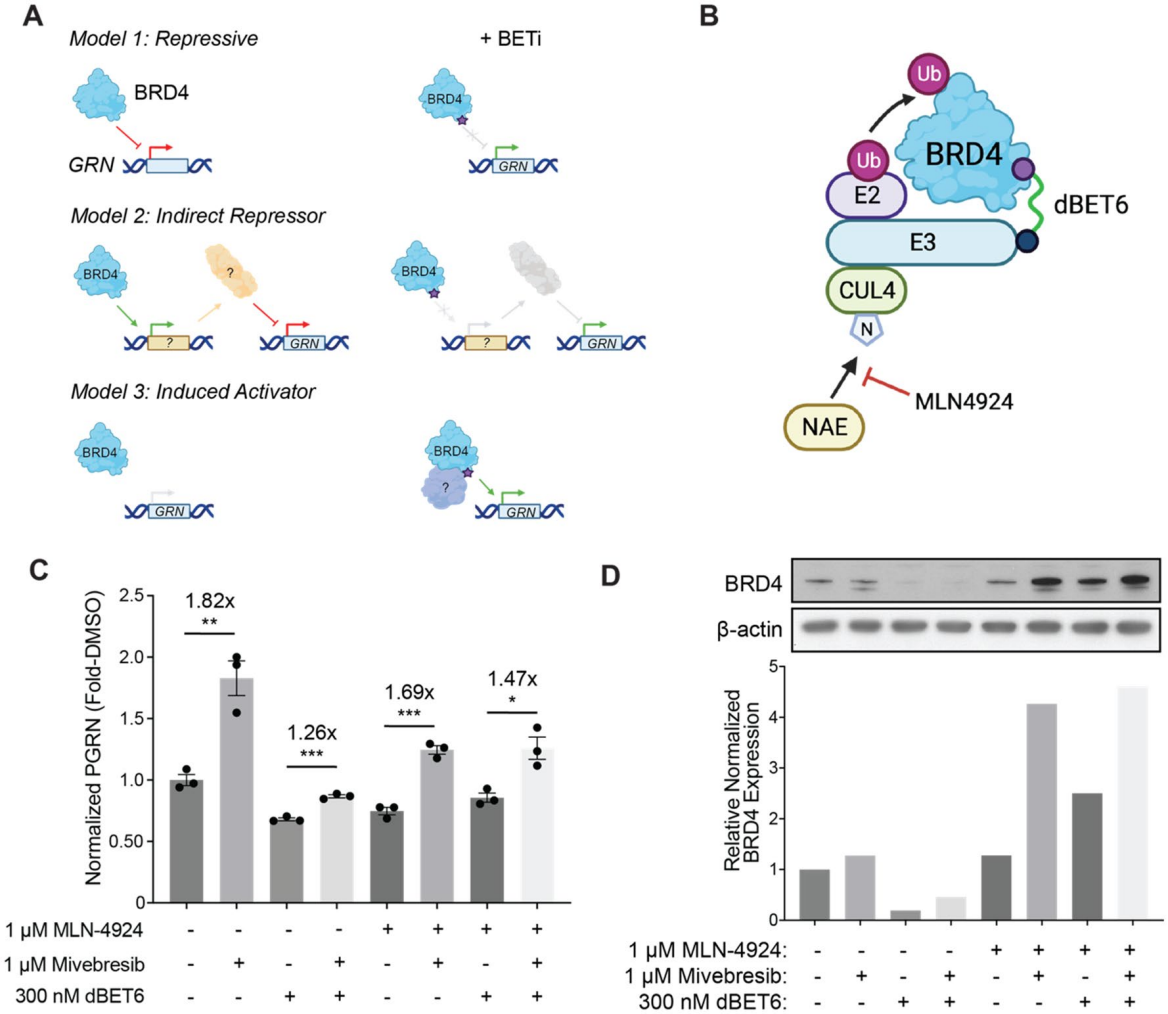
Protein dependencies of BET inhibitors as enhancers of PGRN. (**A**) Mechanistic models for BET inhibition enhancing PGRN protein levels. (**B**) Mechanism of BRD4 degradation through dBET6. (**C**) Human NPCs in various co-treatments with MLN-4924 (1 µM), mivebresib (1 µM), and dBET6 (300 nM). Cells were treated with 1 µM MLN-4924 or DMSO only for 2 h prior to the addition of 300 nM dBET6 or DMSO. Cells were then allowed to incubate for four hours prior to the addition of 1 µM mivebresib or DMSO for 24 h. Data are expressed as mean ± S.E. of 3 biological replicates. (**D**) Immunoblots against BRD4 for human NPCs treated as described in (**C**), with quantification. Significance determined by Dunnett’s Multiple Comparison, *p < 0.05, **p < 0.01, ***p < 0.001, ****p < 0.0001.

As BET proteins are generally thought of as transcriptional activators, our discovery of BET inhibitors as enhancers of *GRN* motivated further mechanistic investigation^33–35^. We initially considered three simplified model systems to elucidate the possible mechanisms by which a BET inhibitor could augment *GRN* expression (Fig. 4A). In the “repressive” model, a BET family member such as BRD4 may direct a negative regulatory complex to the *GRN* promoter/enhancer region, thereby repressing *GRN* transcription, similar to BRD4’s role in guiding G9a to repress autophagy-related genes^59^. For our second proposed model, we posited that BET family members could also function as “indirect repressors” by facilitating the expression of a protein that inhibits *GRN* transcription. Finally, in an “induced activator” model, BET family members may not bind to *GRN* under basal conditions; however, upon exposure to BET inhibitors, they are recruited to *GRN* to activate transcription.

We first tested whether BET proteins operate as negative regulators of *GRN.* If this were the case, acute BET degradation should recapitulate the effects of a BET inhibitor. We thus utilized the previously characterized BET degrader dBET6 to ask whether BET degradation can recapitulate the enhancement of PGRN as seen with mivebresib^60^. dBET6 recruits the E3 ligase CRBN, leading to ubiquitination and consecutive proteolytic degradation of BRD4 (Fig. 4B)^60^. As a control, we further asked whether the effects of dBET6 treatment could be rescued upon pre-treatment with the NEDD8 neddylation inhibitor MLN-4924, which ought to inhibit CUL4-E3 ligase activity and thus prevent degradation of BET proteins (Fig. 4B)^61^.

We treated NPCs with dBET6 or DMSO for 4 h, followed by 24 h of mivebresib treatment or DMSO, without removing dBET6 from the media if present. We first found that degradation of BET family members through dBET6 reduces the ability of mivebresib to enhance PGRN (1.82 × compared to 1.26 × enhancement without and with dBET6, respectively) (Fig. 4C). We next found that pre-treatment with MLN-4924 can attenuate the PGRN-enhancing ability of mivebresib in the presence of dBET6 (1.47 × compared to 1.26 × with and without MLN-4924, respectively) (Fig. 4C). These results reveal that BET proteins are necessary for mivebresib's ability to enhance PGRN, providing evidence against the direct and indirect repressor models.

We next used immunoblotting to validate the ability of our perturbagen combinations to modulate BRD4 protein levels. As expected, treatment with degrader dBET6 significantly reduced BRD4 protein levels, which could be rescued by treatment with MLN-4924 (Fig. 4D, Supplemental Fig. 8). Intriguingly, we found that co-treatment with MLN-4924 and either dBET6 or mivebresib enhanced BRD4 protein levels by ~ 2.5-fold and ~ fourfold, respectively. We hypothesize that dual compensatory and control mechanisms may contribute to this observed effect. Inhibition of BRD4 may result in upregulation of BRD4 through a compensatory mechanism; however, this upregulation would then balanced out by a control mechanism leading to enhanced levels of degradation. MLN-4924 treatment under this model would remove the control mechanism, leading to an enhancement of BRD4 only seen in the co-treatment condition. However, we found that BRD4 protein levels alone did not correlate with PGRN protein levels (Supplemental Fig. 9), suggesting that pharmacological manipulation of BRD4, as opposed to solely increasing BRD4 protein levels, is key to the enhancement of PGRN, or, alternatively, that BRD4 is not the most important BET family member in the regulation of PGRN protein levels.

### Selectivity and kinetic requirements of BET inhibitors as enhancers of PGRN

We next sought to understand whether selective inhibition of the N-terminal or C-terminal bromodomains of BET family members is sufficient to enhance PGRN. While first-generation bromodomain inhibitors such as JQ1 lack selectivity for individual bromodomains of BET family members, the development of highly selective chemical probes for individual bromodomains has helped to elucidate their domain-specific biology and hints towards their unique roles in modulating transcription^62–64^. Understanding the bromodomain-selectivity requirements may provide insights into the therapeutic potential of such compounds. For example, BD2-selective inhibition may produce a more desirable safety profile than that of pan-bromodomain BET inhibitors, as evidenced by pre-clinical toxicology data and clinical advancement of the BD2-selective inhibitors ABBV-744 and RVX-208 (apabetalone)^63,65–67^.

**Figure 5:**
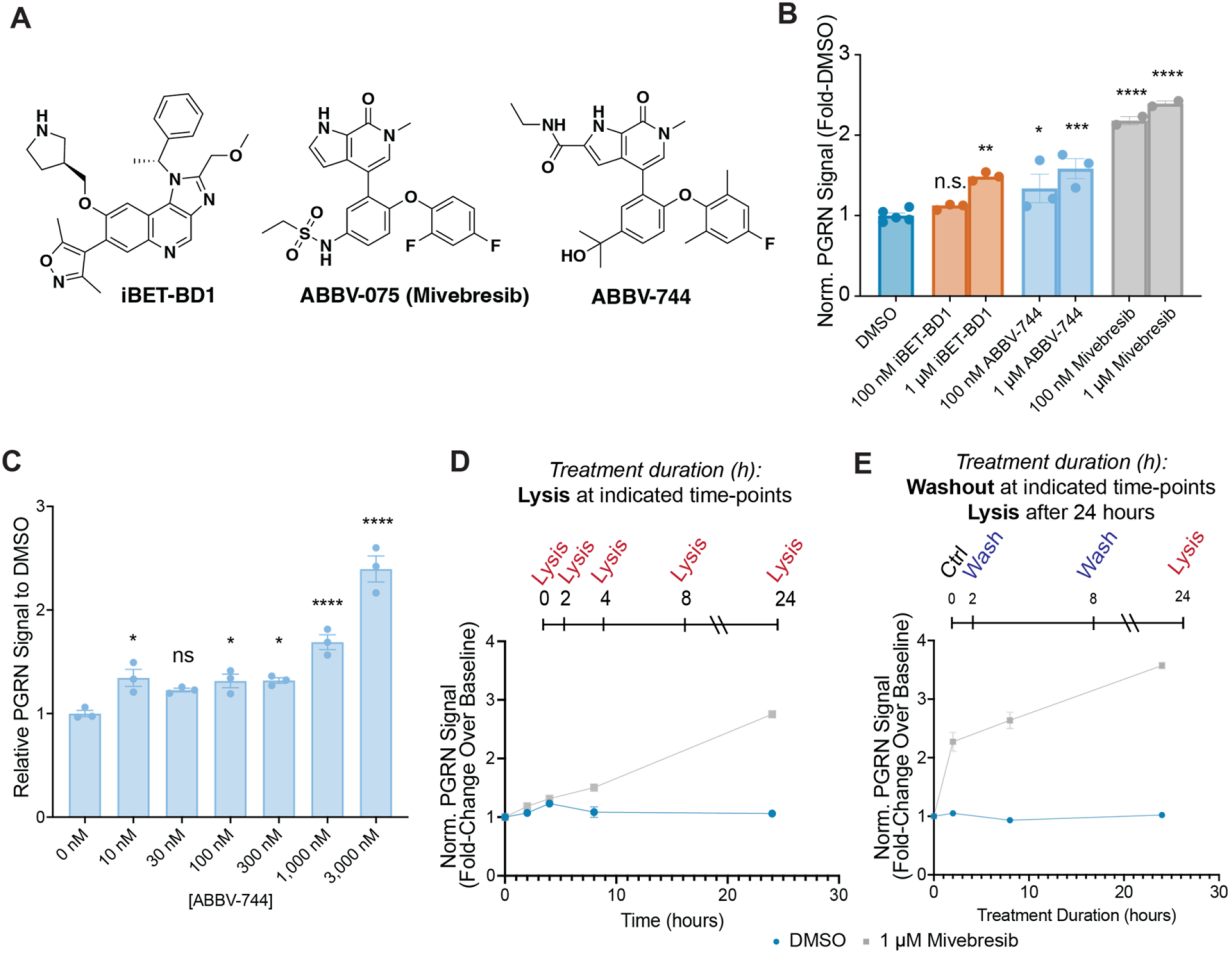
Selectivity and kinetic requirements of BET inhibitors as enhancers of PGRN. (**A**) Structures of selective BET probes used for these studies. (**B**) Selective inhibition of BD2 through ABBV-744 (24 h) is sufficient to enhance PGRN protein levels in human NPCs, while pan-BD engagement through mivebresib produces the largest enhancement. Data are expressed as mean ± S.E. of 2–5 biological replicates. For this experiment, one DMSO data point was excluded due to a suspected issue with the total protein assay (value was 40% of the average of the other five data points, Q-value of 0.886 compared to a Q_crit_ = 0.625 for 6 data points). Significance is compared to the DMSO control group. (**C**) 24-h treatment with ABBV-744 at various doses enhances PGRN in NPCs. (**D**) mivebresib (1 µM) potently upregulates PGRN protein levels at the 8-h and 24-h time points in human NPCs. Data are expressed as mean ± S.E. of 2 biological replicates. Significance is compared to the DMSO control group. (**E**) Treatment with mivebresib for short timeframes, followed by compound washout and then lysis after 24 h from initial compound addition, enhances PGRN protein levels in human NPCs. Data are expressed as mean ± S.E. of 3–6 biological replicates. Significance determined by Dunnett’s Multiple Comparison, *p < 0.05, **p < 0.01, ***p < 0.001, ****p < 0.0001.

To investigate the selectivity requirements of BET inhibitors as enhancers of PGRN, we employed iBET-BD1 (BD1-selective, K_D_ = 31 nM and 5.6 µM against recombinant BRD4 BD1 and BD2, respectively), ABBV-744 (BD2-selective, K_D_ = 435 nM and ≤ 2.0 nM against recombinant BRD4 BD1 and BD2, respectively), and mivebresib (pan-BD, K_D_ ≤ 2.8 nM and ≤ 2.0 nM against recombinant BRD4 BD1 and BD2, respectively) (Fig. 5A)^63,64,68^. Although the BD1 and BD2 domains have high structural homology among BET family members, both iBET-BD1 and ABBV-744 partially achieve selectivity through differential interactions with a divergent His433 (BRD4 BD2) or Asp144 (BRD4 BD1) residue and adjacent water network located in the BC loop of each bromodomain^63,64^.

We first tested the ability of these compounds to enhance PGRN protein levels in human NPCs. We chose to utilize concentrations of 100 nM and 1 µM, with the lower concentrations of iBET-BD1 and ABBV-744 to engage their intended targets selectively. In comparison, both probes may begin to engage both bromodomains at higher concentrations^68^. We found that at a concentration appropriate for selective BD1 inhibition (100 nM), iBET-BD1 was unable to enhance PGRN protein levels, while ABBV-744 treatment significantly enhanced PGRN protein levels by ~ 30% (Fig. 5B). At a concentration (1 µM) where these compounds are expected to engage both bromodomains, both compounds significantly enhance PGRN protein levels by ~ 50–60% (Fig. 5B). Moreover, dual BD1 and BD2 engagement through mivebresib at 100 nM or 1 µM enhances PGRN protein levels by ~ 120–140%, respectively (Fig. 5B). We next probed a greater range of concentrations for ABBV-744. We found that mid to high nM concentrations were sufficient to enhance PGRN protein levels, with higher concentrations producing more substantial PGRN enhancement (Fig. 5C). These results suggest that PGRN enhancement is mediated through the pharmacological engagement of BD2.

Finally, we sought to understand the kinetics of PGRN enhancement in response to BET inhibition. We first treated human NPCs with either mivebresib or DMSO for various timeframes and lysed cells immediately after the duration of each time course (Fig. 5D). We found that mivebresib treatment induced PGRN upregulation in a time-dependent manner, with ~ 1.4-fold and ~ 2.7-fold increases in PGRN observed after 8 and 24 h, respectively (Fig. 5D). We then treated NPCs with mivebresib or DMSO for 2 or 8 h, washed out compounds, and lysed cells after 24 h from the initial compound treatment (Fig. 5E). We found that treatment of NPCs with mivebresib for 2 h, followed by 22 h of incubation post-washout, was sufficient to significantly enhance PGRN protein levels > twofold, with a more pronounced effect after 8 h of mivebresib exposure (Fig. 5E). These data suggest that intermittent exposure to a BET inhibitor would be sufficient to produce therapeutic increases in PGRN protein levels.

### Novel CNS-optimized bromodomain inhibitors can potently engage endogenous BRD4 and enhance PGRN levels in NPCs

While BET family probes such as JQ-1 and I-BET151 can cross the blood–brain barrier, there is a need for more diverse chemotypes of BET inhibitors optimized for use in the study of CNS disorders^69–72^. For PGRN-enhancing compounds to be effective either for use as in vivo tools or potential therapeutics, they must be CNS-permeable and exhibit suitable pharmacokinetic properties. We thus sought to develop a novel chemotype of BET inhibitor with physiochemical properties explicitly optimized for CNS drug discovery efforts.

**Figure 6:**
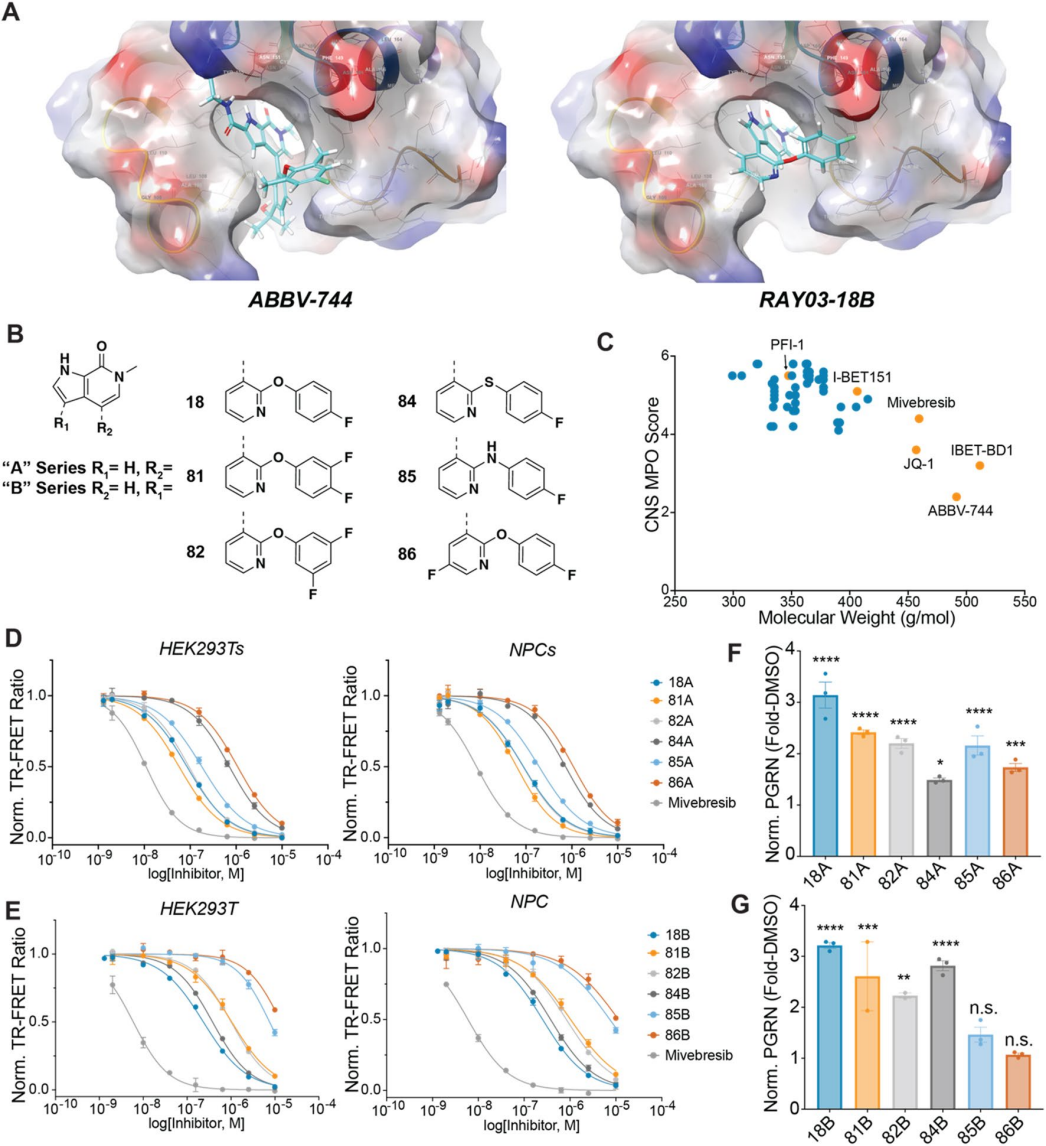
Discovery of novel CNS-optimized BET inhibitors as enhancers of PGRN. (**A**) Optimized poses for ABBV-744 and RAY03-18B docked into BRD4 BDII (PDB: 6ONY). (**B**) Structures of selected novel BET inhibitors. (**C**) Novel BET inhibitors (blue) score highly in CNS MPO scoring in comparison to other common BET inhibitors (orange). (**D**, **E**) Novel compounds can potently engage BRD4 derived from both HEK-293 T and NPC lysates. Data are expressed as mean ± S.E. of 2 biological replicates. (**F**, **G**) Compounds (1 µM) can enhance PGRN in human NPCs after 24 h of treatment. Data are expressed as mean ± S.E. relative to the DMSO control group of 2–3 biological replicates. Data represents compiled experimental runs from different days normalized within each experiment to DMSO. Significance determined by Dunnett’s Multiple Comparison, *p < 0.05, **p < 0.01, ***p < 0.001, ****p < 0.0001.

To guide our synthetic efforts, we utilized CNS Multiparameter Optimization (MPO) scoring, which has previously been used to guide the optimization of drug candidates for desirable ADME attributes, safety profiles, and CNS permeability^73,74^. As a starting point for our synthetic efforts, we docked ABBV-744 into BRD4 BD2, which, as a positive control for our docking studies, produced a pose nearly identical to that of the published bound crystal structure (PBD: 6ONY) (Fig. 6A, left). Our docking analyses of this and other BET inhibitors suggested that the binding site would tolerate alterations in the attachment point of substituents off the core pyrrolopyridine ring system (Fig. 6A, right). For benchmarking and our potency optimization efforts, we thus synthesized analog pairs with equivalent substitutions connected to either the 6 or 5-membered core ring (“A” and “B” series compounds, respectively, Fig. 6B). The compounds we synthesized, including those shown, typically have a molecular weight of less than 400 Da and exhibit higher CNS MPO scores than established BET inhibitors (Fig. 6C).

To analyze the ability of our A and B-series compounds to bind BRD4, we utilized our recently reported CoraFluor-based TR-FRET assay, which allows for the characterization of target engagement toward endogenous BRD4 derived from cellular lysates of cell types of interest^68,75,76^. This assay overcomes a fundamental limitation of commercial in vitro BRD4 TR-FRET assays, which typically utilize a recombinantly-expressed protein that may lack critical post-translational modifications or native binding partners in cells. We found that the A-series analogs were able to potently engage BRD4 derived from both HEK-293 T and NPC lysates, with K_D,app_ values ranging from 30 to 559 nM, establishing the ability of these compounds to engage endogenous BRD4 from relevant cell lines (Fig. 6D, Table 1). In general, we found that the B-series analogs were slightly less potent than those in the A-series, with the most potent derivatives, 18B and 84B, engaging BRD4 derived from NPC lysates with K_D,app_ values of 131 nM and 196 nM, respectively (Fig. 6E, Table 1). Furthermore, using the recombinant, individually purified bromodomains of BRD4 (GST-tagged BD1 and BD2), we found that both our A and B-series compounds bound to recombinant BRD4 BD1 with similar affinities as to BD2, except for 85A, which is ~ 5.5-fold selective for BD1 over BD2 (Supplemental Fig. 10, Supplemental Table 4). Collectively, these assays demonstrate the ability of a new chemotype of pyrrolopyridine-based BET inhibitors to engage BRD4 in a cellular milieu.

Next, we asked whether these pyrrolopyridine compounds could enhance PGRN protein levels in human NPCs. We found that compound treatment (1 µM for 24 h) was sufficient to induce > twofold increase in PGRN protein levels for 18A, 81A, 82A, and 85A, while 84A and 86A produced a less substantial response (Fig. 6F), likely reflecting the reduced potencies of these compounds. We found that of the B-series compounds, 24 h of treatment with 18B, 81B, 82B, and 84B could significantly enhance PGRN protein levels (Fig. 6G). In contrast, 85B and 86B were unable to upregulate PGRN, again matching our expectations given their relatively lower potency in our in vitro target engagement assays. In line with these observations, we found that PGRN enhancement in NPCs correlated (R^2^ = 0.50) with the ability of BET inhibitors to engage BRD4 derived from NPC lysates (Supplemental Fig. 11). These results demonstrate that novel, CNS MPO-optimized BET inhibitors can enhance PGRN protein levels in human NPCs.

Finally, we sought to understand the pharmacokinetic properties of two of our most potent PGRN enhancers, 18A and 18B, in CD-1 mice. While both compounds were cleared quite rapidly, they both readily cross the blood–brain barrier, suggesting that these pyrrolopyridine compounds may be valuable starting points for future medicinal chemistry efforts (Supplemental Fig. 12, Supplemental Table 5).

## Discussion

Here, we have presented the discovery that BET inhibitors enhance PGRN in CNS-relevant cell types, including post-mitotic neurons. We showed that BET inhibitors can enhance a pro-lysosomal gene network in human dermal fibroblasts, restore PGRN to therapeutically relevant levels in NPCs and neurons carrying a *GRN* haploinsufficiency mutation, and mildly enhance PGRN in a microglia-like cell line. These findings suggest that modulation of *GRN* in response to small-molecule epigenetic modifiers may vary depending on the distinct epigenetic states of different cell types, which has implications for translational efforts and, ultimately, the safety and efficacy of the therapeutic strategy. Thus, by screening for PGRN enhancers in human iPSC-derived neuronal cells, we ensured that the epigenetic state of post-mitotic neuronal chromatin was incorporated early in the discovery process.

Represented by the prototypical pyrrolopyridine 18B, we synthesized a novel BET inhibitor chemotype with pharmacological properties optimized for use in the study of CNS disorders. We showed that many of these pyrrolopyridine compounds could potently engage BRD4 derived from cellular lysates and that they enhanced PGRN in human NPCs. Moreover, we demonstrated that two of our compounds could reach the CNS in mice, suggesting the value of this pyrrolopyridine scaffold for future in vivo studies. Our current medicinal chemistry efforts are now focused on refining these compounds’ metabolic properties for optimal exposure, facilitating our evaluation of PGRN regulation within murine models.

Our data suggest that BET inhibitors can rapidly upregulate PGRN protein expression, with significant differences seen after 8 h of compound treatment. Furthermore, we found that rapid compound treatment timeframes followed by a washout period were sufficient to enhance PGRN in human NPCs. If these results translate in vivo, we anticipate prolonged BET target engagement will be unnecessary for PGRN enhancement, which may be a key mechanistic differentiator from using BET inhibitors in oncology settings.

Our mechanistic investigations suggest that while pan-bromodomain inhibitors are most effective at enhancing PGRN, BD2-selective inhibition is sufficient to enhance PGRN protein levels in human NPCs. At the chromatin level, BD1-selective inhibition can efficiently evict BRD4 from chromatin, while BD2-selective inhibition cannot^64^. Furthermore, at the transcriptomic level, alterations induced through selective BD2 inhibition are less substantial than when compared to BD1-selective inhibition^64^. Most clinical-stage BET inhibitors, with exceptions such as RVX-208 (apabetalone) and ABBV-744, bind to BD1 and BD2 with equal potency. However, pan-bromodomain BET inhibitors have significant side effects that may restrict their clinical utility in the chronic treatment of a CNS disorder. In particular, severe, dose-limiting thrombocytopenia and substantial gastrointestinal side effects appear to be an on-target effect of pan-bromodomain BET target engagement^77–79^. Data from clinical trials of RVX-208 suggest that BD2-selective inhibitors may have much more manageable safety and tolerability profiles—an essential requirement for therapeutic uses of small-molecule PGRN enhancers^67,80–83^. Our work thus suggests that current clinical-stage BET inhibitors may have value as repurposed therapeutics for FTD-*GRN*. Our current medicinal chemistry efforts aim to optimize our BET inhibitors’ selectivity for preferential BD2 inhibition and improve their in vivo pharmacokinetic profile.

In this work, we harness the epigenetic plasticity of BET family members for the enhancement of *GRN*. Our finding that BET degraders do not recapitulate BET inhibitors in enhancing PGRN suggests an unexpected gain-of-function mechanism. This finding highlights the unique ability of small molecules to alter pharmacology beyond mere inhibition of protein function. Indeed, this work suggests that small molecule BET inhibitors, despite their effective disruption of bromodomain-acetyl lysine binding, do not invariably inhibit the transcription-enhancing function of BET family proteins. Consistent with this hypothesis, in various cell types, BET inhibition causes downregulation of the majority of genes, while a minority of genes do appear to be upregulated on rapid timescales^64,84^.

The mechanisms driving these responses are likely multifaceted. For example, BET inhibition liberates P-TEFb from the inhibitory 7sk snRNP complex^85^. *GRN* may be a gene that is ‘poised’ for translation by RNA polymerase II, its processivity regulated through recruitment and activation of the positive transcription elongation factor P-TEFb by BRD4^86,87^. Thus, free P-TEFb could then associate with poised RNA Pol II to rapidly enhance *GRN* transcription. Alternatively, BET inhibitors may alter the 3D genome architecture to affect *GRN* regulation. Topologically associated domains (TADs) are organizational units within the eukaryotic genome characterized by high levels of intra-chromatin contact^88^. TADs can enhance the contact frequency of intra-domain regulatory units, promoting coordinated gene enhancement or repression^89^. BET family members work synergistically with various architectural proteins to preserve the structure of TAD boundaries^90–92^. Inhibiting BET proteins could compromise the stability of these boundaries and lead to the formation of different gene co-regulatory networks, enhancing *GRN* transcription. Our future mechanistic studies will focus on understanding how BET inhibitors alter the genomic architecture of NPCs and neurons and relating these epigenetic changes to *GRN* regulation to advance the concept of cell-type specific *GRN* regulation via differential targeting of epigenetic regulatory mechanisms.

Outside of FTD-*GRN*, PGRN enhancement may have broader therapeutic potential against neurodegeneration, as well as neuropsychiatric disorders like bipolar disorder and schizophrenia^93^. Recently, mutations in *GRN* have also been associated with the development of amyotrophic lateral sclerosis, Parkinson’s disease, and Alzheimer’s disease^94^. That *GRN* mutations are associated with other forms of neurodegenerative disease suggests a common mechanism for PGRN in protecting against neurodegeneration. One such proposed mechanism is through maintaining proper lysosomal function. Compromised lysosomal activity is a common feature observed in neurodegenerative diseases, and PGRN deficiency is known to cause lysosomal dysfunction through alterations in lysosomal protein composition and a reduction in lipid homeostasis^11,95^. Furthermore, PGRN enhancement may help reduce excessive neuroinflammatory responses contributing to neuronal degeneration^56,96–98^. We thus speculate that enhancing PGRN expression may aid in maintaining lysosomal homeostasis and tame inflammatory processes, which possibly will have therapeutic applications in neurodegenerative diseases. Together, our work points toward a novel epigenetic target for treating neurodegenerative diseases and provides the field with a starting point for new tool compounds to study BET proteins in the context of CNS disorders.

## Methods

### Human iPSC-derived neural progenitor cell culture

The derivation of healthy control NPCs from iPSCs reprogrammed according to Sheridan et al. from the clinically unaffected human fibroblast cell line GM08330 (8330–8, Coriell Institute for Medical Research, Camden, NJ) has been described (Cheng et al., 2017). Isogenic iPSC containing an allelic series of a pathogenic *GRN* mutation, (*GRN*^+*/*+^, *GRN*^R493^^X/+^ (clone G05), and *GRN*^R493X/R493X^ (clone A02) were acquired from The Jackson Laboratory, which generated these iPSCs as part of a project with the NIH iPSC Neurodegenerative Disease Institute. NPCs were derived from the *GRN*^R493X^ allelic series as described by Cheng et al. (2017), with the exception that nascent NPCs were purified by magnetic-activated cell sorting as described by Bowles et al^26,27,47,99^. Culturing of NPCs occurred as previously described^100^. Briefly, NPCs were cultured in T75 flasks (Corning #353,110, #353,133, or #353,136) and 6-well plates (Corning #353,046, CellTreat #229,105, or CytoOne CC7682-7506), or 24-well plates (Corning #353,047) which were coated first with 20 µg/mL poly-ornithine (Sigma Aldrich #P35307) in ddH_2_O for 2–4 h at 37 °C and then with 5 μg/mL laminin (Sigma Aldrich #L2020) in PBS overnight at 37 °C (coating volumes: 15 mL for T-75 flasks, 2 mL for 6-well plates, 1 mL for 24-well plates). Coated plates were stored at 4 °C until use for up to 5 weeks.

Media for culturing NPCs (“NS media”) consists of 70% DMEM (Dulbecco's modified Eagle's Medium, Gibco #11,995), 30% Ham’s F12 with L-glutamine (Modified Cellgro/Mediatech #10–080-CV), with 1X penicillin/streptomycin (100X, Gibco #15,140–122), and 1X B27 Supplement (50X, Gibco #17,504–044). Media was supplemented with EGF (20 ng/mL, Epidermal Growth Factor, Sigma Aldrich #E9644, prepared as 1000X stock in DMEM), bFGF (20 ng/mL, basic Fibroblast Growth Factor, ReproCELL #03–0002, prepared as 1000X stock in PBS), and heparin (5 μg/mL, Sigma Aldrich #H3149, prepared as 1000X stock in Ham's F12) immediately before use. Growth factor stock solutions were stored at 4 °C for up to one month.

NPCs were maintained at 37 °C with 5% CO_2_ in a humidified atmosphere and passaged at a 1:2 or 1:3 ratio, or seeded with 3–4 × 10^6^ cells per T75 flask. For passaging, confluent cultures were washed with PBS and then treated with TrypLE Select (Life Technologies #12,563,029) until cell detachment. TrypLE treatment was stopped via addition of NPC media. Cells were gently triturated to obtain a single-cell suspension and were centrifuged at 300 rcf for 5 min and then resuspended in NPC media (with growth factors). Coated flasks/plates were first washed with PBS, and then the flasks/plates were allowed to equilibrate at 37 °C/5% CO_2_ with NPC media with growth factors prior to the addition of cells.

### Human iPSC-derived neuron culture

Human iPSC-derived neurons were derived from NPCs by growth factor withdrawal. NPCs were grown on plastic tissue culture ware in 6-well plates (Corning #353,046, CellTreat #229,105, or CytoOne CC7682-7506) that were coated with concurrently with 20 μg/mL polyornithine (Sigma Aldrich #P3655) and 5 μg/mL laminin (Sigma Aldrich #L2020) in dPBS (Thermo Fisher #NC9655718). Plates were stored at 4 °C and washed with 1 mL DPBS prior to plating cells.

NPCs were seeded at 0.5 × 10^6^ cells per well in a poly-ornithine/laminin single coated 6-well plate in 2 mL NPC media with above growth factors. Cells were allowed to incubate at 37 °C for 48–72 h to grow to confluency. After reaching confluency, the media was aspirated, and cells were incubated in NPC media without growth factors for the indicated timeframes, with media changes every 3–4 days.

### Human iPS cell culture

Culturing procedure occurred as previously described, with the exception of iPS cells maintained in mTeSR + media (StemCell Technologies 100-0276) with 1% penicillin/streptomycin (100X, Gibco #15,140-122).^26^. Cells were fed every other day and passaged upon confluency on Matrigel (Corning #354,277)-coated 6-well plates (Corning #353,046, CellTreat #229,105, or CytoOne CC7682-7506).

### Compound preparation and treatment for experiments conducted in Fig. 1

All compounds were purchased from commercial vendors. All compounds were made at 1000 × stocks in DMSO (Sigma Aldrich #D2438), except for sodium valproate, which was dissolved in ddH2O. NPCs and neurons were generated and maintained as described above. For treatment, the stock compounds were diluted 1:1000 in NPC media (final DMSO concentration 0.1%), which was then applied to confluent NPCs or 18-day differentiated neurons derived from confluent NPC cultures for indicated time periods.

### Determination of gene expression changes in human cell culture

RNA was generated from each well of a 6-well treated plate of NPCs or neurons. First, media from each well was aspirated, and washed with 1 mL of dPBS. Wells were then lysed with 1 mL TRIzol Reagent (ThermoFisher #11,596,026). Wells were then incubated at room temperature for 5 min, and then RNA was extracted using the DirectZol RNA MiniPrep Kit (Zymo Research #R2052). RNA was stored at − 80 °C until further use. cDNA was generated using the High-Capacity cDNA synthesis Kit with RNase inhibitor (ThermoFisher #4,368,814) using 1000–1200 ng RNA per reaction. cDNA was either used immediately or stored at -20 °C until use, where it was then diluted 1:4 with DNase/RNase-free water (Invitrogen #10,977–015).

qPCR was conducted using the Roche 480 Light Cycler in a 384-well plate. To each well was added 5 μL of TaqMan 2X Gene Expression Master Mix (ThermoFisher #4,369,510), 0.5 μL of 20X TaqMan primer probe (ThermoFisher, *GRN*: Hs00963707_g1, *GAPDH:* Cat.#432924E), 0.5 μL of DNase/RNase-free H_2_O, and 4 μL of above diluted cDNA. Results were normalized to *GAPDH*, and replicate mean values and standard error of the mean are reported.

### Western blotting and analysis

Cell pellets were collected from each well of a 6- well plate (or lysed directly in-well for 24-well plates), lysed in radio immunoprecipitation assay (RIPA) buffer (Boston BioProducts #BP-115) with EDTA-free protease inhibitors (Sigma Aldrich #4,693,159,001) and a phosphatase inhibitor cocktail (Sigma Aldrich #P5726) and nutated at 4 °C for 15–30 min. Lysates were clarified via centrifugation at > 14,000 rcf at 4 °C for > 10 min and the supernatants were collected. Protein quantification was done with the Pierce bicinchoninic assay (BCA) (Thermo Fisher #23,227). Lysates were then stored between − 20 and − 80 °C until ready for use. Before use, lysates were boiled at 95 °C with SDS loading buffer (New England BioLabs #B7703S) with 1 × DTT (New England BioLabs #B7705S) for 5–15 min, then spun down.

In probing for PGRN, proteins were separated on NuPAGE 4–12% Bis–Tris gels (ThermoFisher #NP0335BOX) in MOPS SDS Running buffer (ThermoFisher #NP0001). To each well, 8 μg total protein was loaded and gels were run at 125 V for 1 h. Gels were then transferred onto 0.45 μm PVDF membranes (ThermoFisher #88,518) or 0.2 μm nitrocellulose membranes (Bio-Rad #1,620,112). Membranes were then blocked in 5% milk in TBST for 1 h and probed overnight at 4 °C with primary antibodies in 5% BSA + 0.02% sodium azide (PGRN antibody: Invitrogen #40–3400, diluted 1:1000 in blocking buffer; GAPDH: Abcam #ab8245, diluted 1:10,000 in blocking buffer). Membranes were then washed with PBS, incubated with secondary antibody in TBST containing 5% milk for 1 h. Secondary antibodies were as follows: for PGRN, anti-rabbit-HRP, Cell Signaling #7074S, 1:2000; for GAPDH: anti-mouse-HRP, Cell Signaling #7076S, 1:2000. Membranes were then washed with PBS for 1 h, and developed with chemiluminescence reagents (Pierce ECL Western Blotting Substrate, ThermoFisher #PI32106; SuperSignal West Dura Extended Duration Substrate, ThermoFisher #PI34076; SuperSignal West Femto Chemiluminescent Substrate, ThermoFisher #PI34096). Protein quantification was done with ImageJ and normalized to GAPDH.

In probing for BRD4, the above procedure was followed, except lysates were generated in M-PER Lysis buffer (Thermo Fisher #78,501) with protease inhibitors (Thermo A32955). Proteins were separated on NuPAGE 4–12% Bis–Tris Gels (Novex NP0335BOX) in MOPS SDS Running buffer (Invtirogen NP0001-02). 3.75 µg total protein was loaded into each well, and gels were run at 200 V for 50 min. Gels were then transferred onto PVDF membranes (Immobilon-P, Millipore IPVH00010). Membranes were then blocked in 5% BSA (Sigma A7906) in TBST (10x, Boston BioProducts IBB-181) for 1 h at room temperature and probed overnight at 4 °C with primary antibodies individually (BRD4: CST 13440S, 1:1000; β-actin: Sigma A1978, 1:10,000) in 5% BSA in blocking buffer. Membranes were washed with TBST for 1 h, and secondary antibodies used were as follows: for BRD4, anti-rabbit-HRP, CST #7074S, for β-actin, anti-Mouse-HRP, CST #7076S. Membranes were washed with TBST for 1 h, and developed with the chemiluminescence reagents used above.

### Quantification of extracellular PGRN

For ELISA experiments, PGRN levels were quantified following the procedure from a human PGRN ELISA kit (AdipoGen #AG-45A-0018YPP-KI01). Briefly, NPCs were grown to confluency according to above procedure in 6-well plates and treated with indicated compounds for 24 h. The cell culture supernatant was collected and incubated with protease inhibitors (Sigma Aldrich, #4,693,159,001) prior to dilution 1:5 in ELISA buffer. Reads were taken using the SpectraMax Plus 384 Microplate Reader (Molecular Devices).

### RNA sequencing of human dermal fibroblasts

Non-immortalized HDF cells (UCSF Memory and Aging Center, FTD25(X)) were cultured in high glucose DMEM with 10% FBS with supplemented L-glutamine and Pen/Strep, and grown in 100 mm plates for treatment. Cells were treated with DMSO or 1 µM mivebresib (1% DMSO) for 24 h. Trizol (Invitrogen) was used to isolate total cellular RNA, according to the manufacturer’s protocol. RNA concentrations were assessed using a NanoDrop spectrophotometer (Thermo Scientific) and samples were diluted to 100 ng/10 µL in nuclease-free water (Ambion). 5 µg of each RNA sample was submitted for processing.

### Enrichment analyses

Gene set enrichment analysis for lysosomal genes (list from Sardiello et al. 2009, Table S1) was performed using GSEA Desktop v3.0, with the following input parameters: number of permutations: 1000, scoring scheme: weighted, normalization mode: meandiv, ranking metric: Signal2Noise, sorting: real^42–44^. TF Enrichment Analysis was performed using ChIP-X Enrichment Analysis Version 3 (https://maayanlab.cloud/chea3/), with upregulated lysosomal genes as the input. Input queried against “Literature” library^45^.

### PGRN quantification using the Jess

PGRN capillary gel electrophoresis experiments were performed using Jess (Bio-Techne) and data were processed and analyzed using Compass for Simple Western software (version 6.0). Runs were conducted using EZ Standard Pack 1 12–230 kDa (Bio-Techne PS-ST01EZ-8), 12–230 kDa pre-filled plates (PS-PP03), 12–230 kDa separation module (SMW004), anti-rabbit HRP detection (DM-001), RePlex (RP-001), and total protein detection modules (DM-TP01, biotin labeling reagent 042-973) according to the manufacturer’s instructions, and with anti-PGRN primary (Abcam ab208777) diluted 1:50 in milk-free antibody dilution buffer (043–524). The following run settings were used for each experiment: separation time: 25 min, separation voltage: 375 V, RePlex purge time: 30 min, biotin labeling time: 30 min, antibody diluent time: 5 min, primary antibody time: 30 min, secondary antibody time: 30 min, total protein HRP time: 30 min. All intensity quantifications are normalized to the total protein assay quantification unless otherwise indicated.

For initial antibody optimization, a lysate of HMC-3 (ATCC CRL-3304) cells engineered to express a C-terminal luciferase fusion to PGRN (unrelated to this current study) was used at the indicated concentrations for the dilution series in M-PER lysis buffer with protease inhibitors, with anti-PGRN (Abcam ab208777) used at the indicated dilutions. For the knockout validation experiments, *GRN*^+/+^, *GRN*^+/−^, *GRN*^−/−^ lysates from iPSCs (cultured as described above) were generated in M-PER with protease inhibitors and used at 1 mg/mL in M-PER lysis buffer.

### Immunocytochemistry of GRN^R493^^X/+^ neurons

An Ibidi 96-well imaging plate (Ibidi 89,626) was coated with 300 µL simultaneously of 20 μg/mL polyornithine (Sigma Aldrich #P3655) and 5 μg/mL laminin (Sigma Aldrich #L2020) in dPBS (Thermo Fisher #NC9655718) and placed into an incubator at 37 °C/5% CO_2_ overnight. Plates were stored at 4 °C and washed with 300 µL dPBS prior to plating cells. *GRN*^*R493*^^*X/*+^ NPCs were grown to confluency on 6-well plates according to the procedure described above, and then were seeded on the Ibidi plate in 300 µL of NS media (without growth factors). Plates were returned to the incubator, and media was replaced every 3 days. After 14 days, plates were removed from the incubator, and 150 µL of media was gently removed from each well. To each well is then added 150 µL 8% formaldehyde (Tousmis 1008A) in PBS, and the plate was allowed to sit at room temperature for 40 min. Wells were then washed with 400 µL PBS, and then to each well was added 400 µL PBS. Plates were then sealed and placed at 4 °C until further use.

Plates were removed from the 4 °C, and to each well is added 300 µL of blocking buffer (2% goat serum (Life Technologies 16,210,064), 1% BSA (Sigma A7906), 0.1% gelatin (Sigma G1890), 0.1% Triton X-100 (Sigma-Aldrich T8787), 0.05% Tween-20 (Sigma-Aldrich P1379), filtered using a 0.45 um filter and stored at 4 °C prior to use. Plates were allowed to block for 1 h at room temperature. After 1 h, 100 µL of 4 × antibody solutions were added to produce wells with the following concentrations: anti-MAP2 (EnCor 7377-030,122) 1:2500, SMI-312R (Covance E04), 1:1000, TUJ1 (Synaptic Systems 302,304) 1:1000. Antibodies were allowed to incubate in wells overnight at 4 °C with light nutation. The next day, antibody solutions were aspirated, and wells were washed with 400 µL PBS × 5. To each condition was then added 200 µL of secondary antibody solutions (for MAP2, anti-chicken 647 (Life Technologies A21449) at 1:500, for SMI-312R, anti-Mouse 647 (Invitrogen A31571) at 1:500, for TUJ1, anti-guinea pig 647 (LifeTech A21450) at 1:500, with phalloidin (1:1000) and Hoechst (1:5000) in blocking buffer. Wells were allowed to incubate at RT for 1 h, after which, wells were washed with 400 µL PBS × 5 prior to imaging. Images taken on the InCell 6000 (GE Healthcare).

### Compound treatment of GRN^R493X/+^ iPSCs, NPCs, and neurons

*GRN*^*R493X/*+^ iPSCs were cultured according to above procedure. At ~ 65% confluency, compound-media solutions were made up in MTeSR + media (1 µM mivebresib, 100 nM panobinostat, 0.1% DMSO final). Media was aspirated from each well, and to each well is added respective compound (or DMSO) solutions. After 24 h of compound treatment, samples were lysed with 500 µL M-PER lysis buffer with protease inhibitor for 15 min at room temperature. Samples were then transferred to 2-mL microcentrifuge tubes and stored in the − 80 °C. Samples were later removed from − 80 °C, allowed to thaw, and spun down at 4 °C at 20 k rcf for 10 min. 350 µL of supernatant was then transferred to fresh 2-mL microcentrifuge tubes. Sample concentration was determined via BCA assay, normalized to 250 µg/mL, and analyzed via capillary gel electrophoresis.

For *GRN*^*R493*^^*X/*+^ NPCs, cells were grown to confluency in 6-well plates according to the above NPC culturing procedure. To each well was directly added 2 µL of 100 µM panobinostat, 10 µM mivebresib, or 100 µM mivebresib, or DMSO only to produce final in-well concentrations of 100 nM or 1 µM, respectively (0.1% DMSO final concentration). For neurons, NPCs were grown to confluency in a 6-well plate, and then growth factor-withdrawal differentiated according to above protocol for 14 days. Compounds were added to neurons by creating 2 × compound solutions in NS media, and performing a half media change to dilute compounds or DMSO only to 1 × (0.1% DMSO final concentration).

After 24 h of compound treatment, wells were aspirated, washed with 2 mL PBS, and then lysed in 250 µL M-PER lysis buffer with protease inhibitors. Samples then collected in 1.5 mL microcentrifuge tubes and were spun down at 4 °C at 20,000 rcf for 10 min. 175 mL supernatant was then transferred to fresh microcentrifuge tubes, and samples were stored in the − 20 °C until further analysis. After determining sample concentration via BCA, NPC samples were normalized to 240 µg/mL, and neuron samples were normalized to 500 µg/mL prior to analysis via capillary gel electrophoresis.

### HMC-3 cell culture

HMC3 cells (ATCC CRL-3304) were cultured in 90% EMEM with EBSS and L-Glutamine (Lonza), 10% FBS (Sigma), 1% penicillin–streptomycin (Gibco), with 1 mM sodium pyruvate (Sigma) and allowed to proliferate at 37 °C with 5% CO_2_. Cells were typically sub-cultured between 1:3 and 1:8, and media was replaced upon acidification.

### Generation of luciferase homology arm

Two PCR reactions were set up using reagents provided by the TrueTag Donor DNA Kit (Invitrogen A42990). Each PCR reaction contained 25 µL of 2 × Phusion Master Mix, 22 µL nuclease-free water, 1 µL of 10 µM forward and reverse primers (Invitrogen 10,336,022, sequences below), and 1 µL of universal C-terminal luciferase template (TrueTag Donor DNA Kit) at 20 ng/µL.

Forward primer: 5′ OOCCTTTGAGGGACCCAGCCTTGAGACAGCTGCTGGGAAGTGGCTCAGGTTCTGGA 3′

Reverse primer: 5′ ZOCCGAGGGCTGCAGAGTCTTCAGTACTGTCCCTCACTTGGCCGATCGCATACAGAG 3′

### Generation of endogenously tagged luciferase reporter line

A 6-well plate of HMC-3 media was prepared and warmed in the incubator at 37 °C/5% CO_2_. To three 500 µL tubes was added 20 µL of supplemented Nucleofector Solution (Amaxa Cell Line Nucleofector Kit V, cat VCA-1003). To two tubes was added 1250 ng Cas9 (0.25 µL, 5 mg/mL, Invitrogen A36498). To one Cas9-containing tube was added 7 µL of luciferase homology arm, prepared as described above, and to the other Cas9-containing tube was added 7 µL DI water. To these two tubes was then added 7.5 pmol sgRNA (Invitrogen A35534, sequence: G*C*C*UUGAGACAGCUGCUGUG with modified scaffold) (1µL, 100 pmol, was diluted into 9 µL nucleofector solution, and then 0.75 µL was added to each tube). To the third tube was added 1 µL of supplied pMaxGFP vector at 0.5 µg/µL. Reagents were allowed to sit at room temperature for 15 min.

HMC-3 cells at passage 6 were trypsinized from a T-75 flask after washing with PBS. Prior to aspiration and trypsinization, the flask appeared to be ~ 90% confluent. Cells were counted via the manual hemocytometer and were at 350,000 cells/mL. Cells were centrifuged at 250 rcf for 5 min at RT. The cell pellet was then re-suspended in 400 µL of supplemented nucleofector solution. After light mixing to ensure homogeneity, 100 µL of this cell suspension was then added to the above tubes containing transfection reagents. Then, using the supplied pipettes, these solutions were transferred into the supplied cuvettes, ensuring that the bottom of the cuvette does not have air bubbles. The cuvette was then capped and labeled. The cuvettes were then taken over to the Amaxa Nucleofector II, and the procedure for THP-1 cells was run using "V-001" protocol for high expression. After each cuvette was nucleofected, 500 µL of HMC-3 media was added to each cuvette. The entire volume was then transferred using the supplied pipettes to one well of the 6-well plate that had been prepared above and warming in the incubator. Plate returned to the incubator.

### Tagged HMC-3 enrichment

Lines were passaged according to normal HMC-3 cell culture protocol. Lines were scaled up to subculturing in T-75 flasks. For initial attempts at puromycin enrichment, HMC-3 cells (both wild-type and the edited line) were grown in T-75 flasks, and to each line is added puromycin at 0.5 µg/mL. Flasks were allowed to incubate for five days in the incubator, with media changed into fresh culture media containing 0.5 µg/mL puromycin. Cultures were then scaled up, and the puromycin-treated cells were seeded onto 96-well plates (Corning 3704) at 5000 cells/well in 100 µL media along with non-puromycin treated cells and cells that were nucleofected but did not receive a homology arm template.

After 1 month of passaging, a T-75 flask was washed with PBS, trypsinized, and then re-suspended in HMC-3 media to 170 cells per mL. 30 µL of this suspension was then seeded onto a white, transparent bottom 384-well plate (Thermo 164,610) using the Multidrop Combi 384 (Thermo Scientific). After 4 days, 15 µL of media was removed from each well, replaced with fresh media, and transferred to a fresh 384-well plate. Active luciferase reagent (Thermo 88,263) was prepared according to manufacturer’s protocol and 15 µL was dispensed to each well using the Multidrop Combi 384. Plate was allowed to nutate at RT for 15 min. Luminescence then read using a Tecan Spark. Several wells were confirmed to have luciferase activity above media alone. After growing to near confluency, one positive well and one negative well were aspirated, washed with 30 µL PBS, and then trypsinized with 40 µL tryplE. The cell suspension was transferred using 40 µL of HMC-3 to a fresh 96-well plate containing 200 µL HMC-3 media. Cultures were grown into progressively larger plate formats over the upcoming weeks. Luciferase activity was validated via sampling the culture media of cells grown in T-75 flasks. Aliquots of cells were frozen down in HMC-3 media with 10% DMSO and are referred to as the “HMC3 GRN-Luc Enriched” line.

### Validation of endogenous tagging

PCR Validation of endogenous knock-in: Cell pellets of the wild-type HMC-3 and enriched GRN-Luc lines were generated from two confluent wells of a 6-well plate and stored at − 80 °C. DNA was isolated from each pellet using the Qiagen DNeasy Blood and Tissue Kit (Qiagen 69,504) following the instructions for cell pellets. 100 ng of isolated genomic DNA for each cell line was then amplified using the following primer pairs, with each primer set containing one primer that amplifies from the insert and the other from the endogenous loci.

Primers were purchased from Integrated DNA Technologies with the following sequences:

Primer ‘F1’: 5′ CACTGCTGTCCTGCTGGCTT 3′

Primer ‘R1’: 5′ GGAGGGGATGGCAGCTTGTAATG 3′

Primer ‘F2’: 5′ GCCCTAGAACCTGGTGCATGAC 3′

Primer ‘R2’: 5′ TCAGGCAGCCTCTGGTACAGCC 3′

For validation via agarose gel electrophoresis, PCR was conducted via addition of 100 ng isolated genomic DNA (< 1 µL each sample) to 25 µL Phusion Flash HF Master Mix, 2X (Invitrogen F548L) followed by addition of 1 µL of each of the following primer pairs as 25 µM stock solutions in TE buffer (to 500 nM final concentrations). Solutions were then filled with DI water to 50 µL and then run on the Thermocycler (Bio-Rad T100) with the following protocol: 98 °C for 1 min, [98 °C for 15 s, 68 °C for 10 s, 72 °C for 30 s] with the bracketed component repeated 32x, followed by 72 °C for 10 min and a hold at 4 °C.

PCR products were visualized on a 2% agarose gel (UltraPure Agarose, Invitrogen 16,500–500) made in 1 × TAE buffer (Thermo Fisher Scientific 15-558-026) with 1 µL ethidium bromide. PCR reactions were diluted into a 6 × loading buffer containing Xylene Cyanol (Sigma-Aldrich X4126). 30 µL sample was loaded into each lane, and Quick-Load Purple 1 kb plus DNA Ladder (NEB N0550S). Gel was run for 2 h in TAE buffer at 90 V.

For sequencing, PCR was conducted via addition of 100 ng isolated genomic DNA (< 1 µL each sample) to 25 µL Phusion Flash HF Master Mix, 2X (Invitrogen F548L) followed by addition of 1 µL primers F1/R2 and separately F2/R1 from 25 µM stock solutions in TE buffer (to 500 nM final concentrations). Solutions were then filled with DI water to 50 µL and then run on the Thermocycler (Bio-Rad T100) with the following protocol: 98 °C for 10 s, [98 °C for 1 s, 68 °C for 55 s, 72 °C for 15 s] with the bracketed component repeated 40x, followed by 72 °C for 1 min and a hold at 4 °C.

PCR products were visualized on a 2% agarose gel (UltraPure Agarose, Invitrogen 16,500–500) made in 1 × TAE buffer (Thermo Fisher Scientific 15-558-026) with 1 µL ethidium bromide. PCR reactions were diluted into a 6 × loading buffer containing Xylene Cyanol (Sigma-Aldrich X4126). 30 µL sample was loaded into each lane, and Quick-Load Purple 100 bp DNA Ladder (NEB N0551S). Gel was run for 2 h in TAE buffer at 90 V.

Bands were excised, and DNA was isolated using the Qiaquick PCR Purification Kit (Qiagen 28,104) following manufacturer’s instructions. A small aliquot of purified DNA was then validated on a 2% agarose gel, and the remainder of the aliquot was sequenced using Next-Generation Sequencing by the CCIB DNA Core Facility at Massachusetts General Hospital (Cambridge, MA).

### PGRN-Luc compound screening assays

HMC3 PGRN-Luc cells were trypsinized from confluent T-75 flasks and re-suspended in HMC-3 media at 150,000–200,000 cells/mL. 30 µL of cell suspension was then seeded onto each well of black, 384-well clear-bottom plates (Corning 3712) using the Multidrop Combi (Thermo Fisher). Cells were allowed to adhere to plates in the incubator at 37 °C/5% CO_2_ for 6–8 h. After 6–8 h, compounds were added to each well using the D300e (Tecan) compound dispenser, and all wells were DMSO-normalized (0.3%). Cells were allowed to incubate with compounds for indicated timeframes.

Plates were removed from the incubator and allowed to cool to RT. 15 µL of media was removed from each well using a multichannel pipette and added to separate Corning 3712 plates for a read of “secreted” PGRN. To both the media-containing plates as well as the cell-containing plates (with 15 µL media remaining) is then added 15 µL of active luminescence reagent (Thermo 18,636) which was generated according to manufacturer’s instructions. Plates were allowed to nutate at RT for 15 min prior to reading using the EnVision (PerkinElmer). For calculation of the internal PGRN-Luc signal, the secreted signal (15 µL media) was subtracted from the signal of the cell-containing plates (with 15 µL media remaining). For “total” PGRN-Luc signal, a sample of media was not aliquoted into a separate plate, and instead, 30 µL of active luminescence reagent was added directly to each well.

For the mivebresib and panobinostat time-course experiment, the above procedure was followed, with the exception that cells were seeded at 66,600 cells/mL in 30 µL media, and allowed to grow overnight prior to compound addition for the indicated timeframes.

### Probing BET protein dependency

NPCs were cultured normally in 6-well plates as described previously. Upon reaching confluency, DMSO or MLN-4924 (Cayman #15,217, 10 mM stock in DMSO) were diluted 1:5000 into NPC media with growth factors. 1 mL of media was aspirated from the cell cultures, and 1 mL of MLN-4924 or DMSO dilutions were then added to respective wells (final well concentration: 1 µM MLN-4924 in select wells, 0.01% DMSO for all wells). Plates returned to the incubator. After 1 h, to the indicated wells is directly added 1 µL 600 µM dBET6 in DMSO (MedChem Express HY-112588) for a final concentration of 300 nM, or 1 µL DMSO. Plates returned to the incubator. After 4 h, to the indicated wells is directly added 1 µL 2 mM mivebresib (MedChem Express HY-100015) in DMSO for a final concentration of 1 µM, or 1 µL DMSO. All plates were returned to the incubator overnight. The next day, 24 h from the addition of mivebresib, plates were removed from the incubator, and all wells were washed with 2 mL PBS, and then lysed in 300 µL M-PER lysis buffer with protease inhibitors. Cells were allowed to lyse for 15 min at 4 °C with light nutation, and then lysate was transferred to a 1.5-mL Eppendorf tube which was then spun down at 20 k rcf at 4 °C for 10 min. 200 µL of supernatant was then transferred to a fresh, labeled Eppendorf rube, which was then stored at − 20 °C prior to further analysis. Sample concentration was quantified using the BCA assay as described above, and normalized to 250 µg/mL in M-PER lysis buffer prior to PGRN quantification using the Jess.

### Probing selectivity requirements for PGRN enhancement

NPCs were cultured normally as described previously. 6-well plates were seeded at 300,000 cells/well in 2 mL of NPC media with EGF, bFGF, and heparin as described above. Cells were allowed to grow to confluency for three nights at 37 °C with 5% CO_2_. After cells reached ~ 100% confluency, iBET-BD1 (Glixx Laboratories, GLXC-22368), ABBV-744 (MedChem Express, HY-112090), and mivebresib (MedChem Express HY-1000015) were diluted from 500 µM stocks in DMSO 1:500 into NPC media with growth factors to create 1 µM compound solutions (0.2% DMSO final). These solutions were then diluted 1:10 in NPC media with growth factors containing 0.2% DMSO to make 100 nM compound solutions. Treatment wells were washed with 2 mL PBS prior to addition of 2 mL NPC media with growth factors containing compound (or DMSO only) at indicated concentrations (3 replicates per condition, 6 DMSO replicates). Plates were then allowed to incubate at 37 °C with 5% CO_2_ for 24 h.

After 24 h, cells were removed from the incubator, washed with 1 mL PBS, and then lysed with 500 µL M-PER lysis buffer (Thermo Fisher #78,501) with 1 cOmplete Mini, EDTA-free protease inhibitor added (Sigma-Aldrich#11,836,170,001) per 15 mL lysis buffer. Plates were allowed to nutate at 4 °C for 15 min before transferring the lysate to 1.5 mL Eppendorf tubes, which were then spun down at 20,000 rcf at 4 °C for 10 min. 400 µL of the supernatant was then transferred to fresh 1.5-mL Eppendorf tubes, which were then frozen down at − 80 °C prior to further use. Samples were removed from the − 80 °C and concentrations were determined via BCA assay (Thermo Scientific #23,227) using a pre-diluted BSA standard (Life Technologies #23,208) on a 96-well polystyrene plate (Thermo Fisher #12,565,500). Samples were diluted to 0.25 mg/mL using M-PER lysis buffer prior to PGRN quantification using the Jess.

### ABBV-744 full dose–response in human NPCs

NPCs were cultured as described above, and seeded onto coated 24-well plates at 150,000 cells/well in 1 mL NPC media with growth factors. Plates were allowed to grow to ~ 100% confluency for three nights at 37 °C with 5% CO_2_. After reaching confluency, wells were washed with 1 mL PBS, and then to wells is added 2 mL NPC media with growth factors containing ABBV-744 at the indicated concentrations (or DMSO only) (DMSO concentration: 0.6% DMSO). Plates were allowed to incubate overnight at 37 °C with 5% CO_2_ for 24 h. After 24 h, wells were washed with 1 mL PBS, and then lysed in-well using 100 µL M-PER lysis buffer with 1 cOmplete Mini, EDTA-free protease inhibitor added per 15 mL lysis buffer. Lysis was allowed to occur for 10 min at 4 °C, and then crude lysates were transferred to 1.5-mL Eppendorf tubes and spun down at 20 k rcf for 10 min at 4 °C. The supernatant was then transferred to fresh 1.5-mL Eppendorf tubes and stored at -20 °C until further use. Samples were normalized to 0.25 mg/mL in M-PER prior to running on the Jess.

### Mivebresib time-course experiment in NPCs

NPCs were cultured as described above, and seeded onto coated 24-well plates at 150,000 cells/well in 1 mL NPC media with growth factors. Plates were allowed to grow to ~ 100% confluency for three nights at 37 °C with 5% CO_2_. After reaching confluency, wells were washed with PBS, and then to wells is added 1 mL of 1 µM mivebresib (diluted 1:50,000 from 50 mM DMSO stock) in NPC media with growth factors, or media containing DMSO only. At the indicated time-points (0, 2, 4, 8, and 24 h), select wells were washed with 1 mL PBS, and then lysed using 100 µL M-PER lysis buffer with cOmplete Mini, EDTA-free protease inhibitor (1 tablet per 15 mL M-PER). Wells at the “0 h” timepoint did not receive either compound or media containing DMSO (they were immediately lysed as a baseline). Crude lysates were stored at -20 °C while waiting for the conclusion of the time-course experiment. After the final samples were generated, all samples were spun down at 4 °C for 10 min at 20 k rcf. 50 µL of supernatant was then carefully transferred to new 1.5-mL Eppendorf tubes and stored at -20 °C until analysis on the Jess. Prior to running samples on the Jess, the concentration of samples was determined via BCA assay and samples were normalized to 0.25 mg/mL using M-PER lysis buffer.

### Mivebresib washout experiment in NPCs

NPCs were cultured as described above and seeded at 300,000 cells/well in 2 mL NPC media with growth factors. Cells were allowed to grow to ~ 100% confluency over three nights. The media was then replaced with 2 mL of 1 µM mivebresib (diluted 1:10,000 from 10 mM stock in DMSO) or DMSO-containing media only. Plates were then placed into the incubator at 37 °C/5% CO_2_. At the indicated timepoints (2 h, 8 h, 24 h), plates were removed from the incubator, washed with 2 mL PBS, and then fresh NPC media with growth factors containing DMSO only was added to each well. For the “0 h” timepoint, all wells were filled with DMSO-containing NPC media with growth factors. The 2 h and 8 h timepoints were returned to the incubator for 22 h and 16 h, respectively. After 24 h had elapsed since the beginning of the experiment, all plates were washed with 2 mL PBS, and then lysed with 300 µL M-PER with cOmplete Mini, EDTA-free protease inhibitor (1 tablet per 25 mL M-PER). Crude lysates were then transferred to 1.5-mL Eppendorf tubes and spun down at 20 k rcf for 10 min at 4 °C, and then 150 µL of supernatant was pipetted into fresh 1.5-mL Eppendorf tubes. Samples were placed at -20 °C prior to further analysis. After concentration determination via BCA assay, samples were normalized to 150 µg/mL, and then samples were analyzed using the Jess.

### Preparation of HEK293T and NPC cell extracts for target engagement studies (Figs. 3D and 4C)

A cell pellet from one 15 cm dish (~ 25 M cells) of HEK293T cells, or from one confluent T-75 flask 8330-8 NPC cells (~ 9 M cells), was allowed to thaw on ice and cells were suspended in 400 μL lysis buffer (25 mM HEPES, 150 mM NaCl, 0.2% (v/v) Triton X-100, 0.02% (v/v) TWEEN-20, pH 7.5 supplemented with 2 mM DTT and one Roche cOmplete, Mini, EDTA-free protease inhibitor cocktail tablet (Sigma 11,836,170,001) per 7 mL. Cells were homogenized via passage through a 27.5-gauge needle 5 times, and the resulting mixture was incubated with slow, end-over-end mixing at 4 °C for 15 min. The lysate was clarified via centrifugation at 16,100 × g for 10 min at 4 °C then 800 μL (1:3 dilution) dilution buffer (25 mM HEPES, 150 mM NaCl, 0.005% (v/v) TWEEN-20, pH 7.5) was added and the lysate was re-clarified at 16,100 × *g* for 10 min at 4ºC. Total protein was quantified via detergent-compatible Bradford assay (ThermoFisher 23,246). The lysate was used fresh or flash-frozen in liquid nitrogen and stored at − 80 °C in single-use aliquots.

### TR-FRET measurements

Unless otherwise noted, experiments were performed in white, 384-well microtiter plates (Corning 3572 or PerkinElmer ProxiPlate-384 Plus) in 30 μL or 10 μL assay volume, respectively. TR-FRET measurements were acquired on a Tecan SPARK plate reader with SPARKCONTROL software version V2.1 (Tecan Group Ltd.), with the following settings: 340/50 nm excitation, 490/10 nm (Tb), 520/10 nm (FITC) emission, 100 μs delay, and 400 μs integration. The 490/10 and 520/10 nm emission channels were acquired with a 50% mirror and a dichroic 510 mirror, respectively, using independently optimized detector gain settings unless specified otherwise. The TR-FRET ratio was taken as the 520/490 nm intensity ratio on a per-well basis.

### Determination of equilibrium dissociation constant (K_D_) of JQ1-FITC toward individual recombinant bromodomains and K_D,app_ toward endogenous BRD4 in HEK293T/NPC lysates

Assays were performed as described^68^. Recombinant BRD4(BD1) and BRD4(BD2) were purchased from BPS Biosciences, Inc and Epicypher, Inc (GST-BRD4(BD1), 31,040; GST-BRD4(BD2), 15–0013, respectively). Recombinant bromodomains were diluted to 0.5 nM in assay buffer (25 mM HEPES, 150 mM NaCl, 0.5 mg/mL BSA, 0.005% TWEEN-20, pH 7.5) with 2 nM CoraFluor-1-labeled GST V_H_H (ChromoTek ST-250), then JQ1-FITC was added in serial dilution (c_max_ = 100 nM) using an HP D300 digital dispenser and allowed to equilibrate for 2 h at room temperature before TR-FRET measurements were taken. Nonspecific signal was determined with 50 μM JQ1-Acid, and data were fitted to a One Site–Specific Binding model in Prism 9. The *K*_D_ determined from the one-site model was then used in Eq. 1 to adjust for a two-site model due to the dimeric GST protein:

1 $${K}_{D,2-site}={K}_{D,1-site}\times (1+\sqrt{2})$$

For the profiling of endogenous BRD4, cell lysates as prepared above were diluted to 0.8 mg/mL total protein in 1:3 lysis buffer:dilution buffer with 0.5 nM rabbit anti-BRD4 antibody (Cell Signaling Technology; E2A7X) and 1 nM CoraFluor-1-labeled anti-rabbit Nano-Secondary (ChromoTek CTK0101). JQ1-FITC was added in serial dilution (c_max_ = 200 nM) using an HP D300 digital dispenser and allowed to equilibrate for 2 h at room temperature before TR-FRET measurements were taken. Nonspecific signal was determined with 50 μM JQ1-Acid, and data were fitted to a One Site–Specific Binding model in Prism 9. The *K*_D,app_ determined from the one-site model was then adjusted for a two-site model using Eq. 1.

### TR-FRET ligand displacement assays for recombinant bromodomains and endogenous BRD4 in HEK293T/NPC lysate

The following assay parameters have been used: (i) 4 nM GST-BRD4(BD1), 4 nM CoraFluor-labeled GST V_H_H, 20 nM JQ1-FITC in assay buffer, (ii) 4 nM GST-BRD4(BD2), 4 nM CoraFluor-labeled GST V_H_H, 20 nM JQ1-FITC in assay buffer, (iii) HEK293T or NPC cell lysate at 0.8 mg/mL total protein, 1 nM rabbit anti-BRD4 antibody, 2 nM CoraFluor-labeled anti-rabbit Nano-Secondary, 20 nM JQ1-FITC. For all experiments, test compounds were added in serial dilution (c_max_ = 10 μM) using an HP D300 digital dispenser and allowed to equilibrate for 2 h at room temperature before TR-FRET measurements were taken. The assay floor (background) was defined with the 10 μM mivebresib dose, and the assay ceiling (top) was defined via a no-inhibitor control. Data were fitted to a four-parameter dose–response model in Prism 9.

### Calculation of inhibitor K_D_ and K_D,app_ values from measured TR-FRET IC_50_

For TR-FRET ligand displacement assays with recombinant bromodomains, we have determined the *K*_D_ of the respective fluorescent tracer (JQ1-FITC) under each assay condition. For endogenous BRD4 in whole cell extract, we have determined the *K*_D,app_ of JQ1-FITC under each assay condition. Inhibitor *K*_D_ and *K*_D,app_ values have been calculated using Cheng-Prusoff principles, outlined in Eq. 2 below:

2 $${K}_{D} or {K}_{D,app}= \frac{{IC}_{50}}{1+\frac{[S]}{{K}_{X}}}$$

where IC_50_ is the measured IC_50_ value, [S] is the concentration of fluorescent tracer, and *K*_X_ is the *K*_D_ or *K*_D,app_ of the fluorescent tracer for a given condition^101^.

### BET inhibitor treatment of NPCs followed by PGRN quantification

NPCs were cultured as described above. NPCs were seeded on coated 6-well plates at 300,000 cells per well in 2 mL NPC media with growth factors and were allowed to grow to confluency over 3 nights at 37 °C with 5% CO_2_. After cells reached confluency, compound-media solutions were added to each well, either through a full media replacement into fresh NPC media with growth factors with 1 × compound, or through dilution of 20 × compound solutions in fresh NPC media with growth factors directly into each well.

After 24 h of compound treatment, media was aspirated, cells were washed with 2 mL PBS, and then to each well was added 1 mL PBS. Cells were then scraped using a cell lifter (Costar 3008), and then transferred to 1.5-mL eppendorfs where they were then spun down at 400 rcf at room temperature for 5 min. The supernatant was then aspirated off, and pellets were stored in the − 80 °C until further analysis.

Pellets were removed from the − 80 °C and allowed to thaw briefly on ice. Pellets were lysed for 10–15 min at 4 °C with 100 µL M-PER lysis buffer with 1 cOmplete Mini, EDTA-free protease inhibitor. Lysates were then clarified via spinning at 20 k rcf at 4 °C for 10 min, and then the supernatant was then transferred to new 2-mL Eppendorf tubes. Concentrations were determined via a BCA assay, and samples were normalized to 250–500 µg/mL prior to PGRN quantification via the Jess.

### Pharmacokinetic analyses of 18A and 18B

Analysis of pharmacokinetic parameters of 18A and 18B was performed by the Preclinical Pharmacology Core Lab (UT Southwestern Medical Center). Animal work described in this manuscript has been approved and conducted under the oversight of the UT Southwestern Institutional Animal Care and Use Committee and were carried out in accordance with relevant guidelines and regulations. UT Southwestern uses the “Guide for the Care and Use of Laboratory Animals” when establishing animal research standards*.* All methods are reported in accordance with ARRIVE guidelines. Compounds were suspended in 10% DMSO/20% PEG400/70% (30% HPβCD in water) at 0.484 mg/mL (18A) or at 0.5 mg/mL (18B). Compounds were administered to male CD1 mice (Charles River Laboratories, Wilmington, MA) IV via the tail vein at 5 mg/kg (0.30 mL dose volume for 18A, 0.35 mL dose volume for 18B). Mice were randomly assigned to time point groups and all investigators were aware of these assignments. After inhalation overdose of CO_2_, blood and tissues were collected from three mice per timepoint, at 5, 15, 30, 60, 240, 360, and 480 min post dosing, such that a mean concentration and standard deviation about the mean could be reported. Blood and tissues were also collected from mice dosed with Vehicle only at 480 min. Blood was collected using the anticoagulant ACD (acidified citrate dextrose) and plasma was isolated from the blood by centrifugation at 4 °C, 9600 × g for 10 min. After rinsing with PBS, tissues were weighed, snap-frozen in liquid nitrogen and stored at − 80 °C until processing. Brain was homogenized in a three-fold volume of PBS (weight by volume). Plasma and brain homogenates were extracted using a two-fold volume of methanol containing 0.15% formic acid and 37.5 ng/ml of an internal standard, n-benzylbenzamide (Sigma, St. Louis, MO). Samples were vortexed for 15 s, incubated at RT for 10 min and then centrifuged at 16,100 × g for 5 min at 4 °C. The supernatant was spun a second time and then analyzed by LC–MS/MS using a Sciex (Framingham, MA) 4000QTRAP® (positive ESI, multiple-reaction monitoring mode) after separation on an Agilent (Santa Clara, CA) C18 XDB column (5 micron packing, 50 × 4.6 mm size). The following separation gradient was used (Buffer A: water with 0.1% formic acid; Buffer B: MeOH with 0.1% formic acid): 0–1 min 5% B, 1–1.5 min gradient to 100% B, 1.5–3 min 100% B, 3–3.2 min gradient to 5% B, 3.2–4.5 min 5% B. For 18A, the transition (m/z) of 336.0/305.0 was monitored (retention time: 2.08 min); for 18B, the transition (m/z) of 336.1/169.0 was monitored (retention time: 2.09 min). The transition 212.1/91.1 (retention time 2.05 min) was monitored for the internal standard, n-benzylbenzamide. Tissues and plasma from untreated mice were used as controls to establish background noise for LC–MS/MS analysis of compound levels. Pharmacokinetic parameters were calculated using sparse sampling and the noncompartmental analysis tool in WinNonlin (Certara Corp, Princeton, NJ) for plasma and brain. Because mice were not perfused prior to collection of brain, the amount of compound estimated to be in the vasculature of the brain was subtracted using the measured plasma concentration and literature values for the volume of residual blood (30 µl/g tissue) in the brain Kwon^102^.

### Modeling of RAY03-18B and ABBV-744 in BRD4 BDII

For docking studies, Schrödinger (version 2022-1, Schrödinger, LLC, New York, NY, 2021) GlideXP docking protocol was utilized^103–105^. PDB 6ONY (BRD4 BDII with bound ABBV-744) was first prepared using default settings in the Protein Preparation Wizard^106^. Waters were removed beyond 3 Å of heteroatoms, and H-bond assignment was optimized. Highly surface exposed waters were manually inspected and removed prior to docking. Receptor Grid Generation was then performed using ABBV-744 as the reference for the receptor site.

Structures of RAY03-18B and ABBV-744 were generated using LigPrep using OPLS4 with protonation states determined using Epik at pH 7 ± 2.0^107,108^. Ligands were then docked into the previously mentioned generated grid using Glid XP docking, with default settings.

### Calculation of CNS MPO Scoring for Synthesized Compounds

CNS MPO scores were computed from structures that were input into CDD (collaborativedrug.org).

### Chemistry

^1^H and ^13^C NMR spectra were recorded on a Bruker AV-III-400 or 500 MHz NMR spectrometer. Chemical shifts are reported in δ values in ppm downfield from TMS as the internal standard. ^1^H NMR data are reported as follows: chemical shift, multiplicity (s = singlet, d = doublet, t = triplet, q = quartet, b = broad, m = multiplet), coupling constant (Hz), integration. ^13^C chemical shifts are reported in δ values in ppm downfield from TMS as the internal standard. Low resolution mass spectra were obtained on Waters Acquity Ultra Performance LC with electrospray ionization and SQ detector by injecting sample in a steady flow of 1 mM ammonium acetate in 20% water-acetonitrile at the rate of 0.2 mL/min. The purity of compounds were determined by analytical HPLC, performed on a Shimadzu Prominence-HPLC with ELSD PDA multi, and a Gemini NX C-18 column (250 × 4.6 mm, 5μ) with mobile phase (A) 0.1% Formic Acid in water and mobile phase (B) 0.1% Formic Acid in Acetonitrile using following gradient of B/A (0 min, 10%), (5 min, 90%), (6 min, 95%), (10 min, 95%), (10 min, 10%) and (14 min, 10%) at 1.0 mL/min flow rate. Analytical thin layer chromatography was performed on 250 μm silica gel F_254_ plates. Preparative thin layer chromatography was performed on 1000 μm silica gel F_254_ plates. Flash column chromatography was performed employing 230–400 mesh silica gel.

### Synthesis of “A” series compounds:


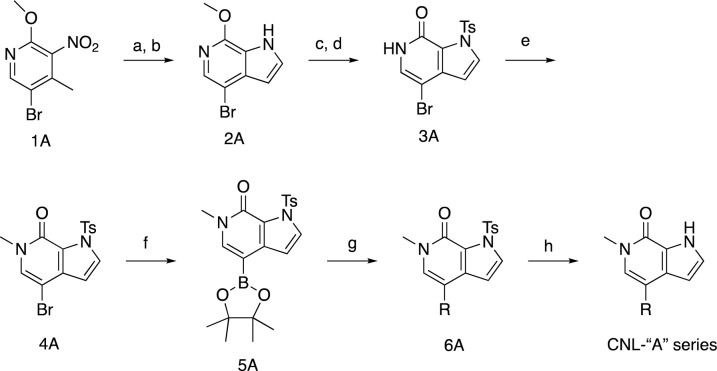


***Reagents and conditions*** *: (a) LiOMe,1,1-dimethoxy-N,N-dimethylmethanamine, DMF, 100 °C; (b) Ra-Ni, MeOH, rt; (c) NaH, pTsCl,THF, rt; (d) 4 M HCl, 1,4-dioxane, 65 °C; (e) Cs*_*2*_ *CO*_*3*_ *, MeI, 1,4-dioxane, rt; (f) bis(pinacolato)diboron, KOAc, Pd*_*2*_ *dba*_*3*_ *, X-phos, 1,4-dioxane, 90 °C; (g) Aryl bromides (18-P, 81-P, 82-P, 84-P, 85-P, 86-P, K*_*2*_ *CO*_*3*_ *, Pd*_*2*_ *dba*_*3*_ *, meCgPPh, 1,4-dioxane-water (4:1), 65 °C; (h) K*_*2*_ *CO*_*3*_ *, MeOH–water, 85 °C.*

*4-Bromo-7-methoxy-1H-pyrrolo[2,3-c]pyridine (2A)*

To a solution of 5-Bromo-2-methoxy-4-methyl-3-nitropyridine (**1A**) (10.0 g, 40.5 mmol) in DMF (100 mL) was added 1 M lithium methanolate in methanol (2.5 mL, 2.5 mmol, 1 M) and heated to 100 °C. To this reaction mixture 1,1-dimethoxy-*N,N*-dimethylmethanamine (40 mL, 296 mmol) was added dropwise over 10 min. The reaction mixture was further stirred at 100 °C for 5 h and then allowed to cool to ambient temperature. Then 600 mL water was added gradually to the reaction mixture. The resulting precipitate was collected by vacuum filtration, washed with water (50 mL), and dried to provide (*E/Z*)-2-(5-bromo-2-methoxy-3-nitropyridin-4-yl)-*N,N*-dimethylethenamine (**1A-a)** (10.5 g, 86% yield) as bright red solid. It was reacted further without purification. MS (ESI^+^) m/z 302.0 (M + H)^+^.

(*E/Z*)-2-(5-Bromo-2-methoxy-3-nitropyridin-4-yl)-*N,N*-dimethylethenamine (5 g, 16.6 mmol), iron (7 g, 125.4 mmol) and ammonium chloride (15 g, 280.4 mmol) were dissolved in 5:3:2 mixture of THF: Ethanol: Water (50 mL). The reaction mixture was stirred at room temperature for 12 h. After TLC analysis (20% EtOAc in Hexanes) showed complete consumption of starting material, the reaction mixture was filtered filter and extract product with ethyl acetate (30 mL × 2 times). Finally, purify the concentrated residue by silica gel column chromatography (5–40% EtOAc in Hexane) to provide compound **2A.** (5 g, 38% yield over two steps). MS (ESI^+^) m/z 226.9 (M + H)^+^. ^1^H NMR (500 MHz, CDCl_3_) δ 8.70 (bs, 1H), 7.84 (s, 1H), 7.31 (t, *J* = *2.5 *Hz, 1H), 6.57 (t, *J* = *2.5 *Hz, 1H), 4.08 (s, 3H).

#### 4-Bromo-1-tosyl-1H-pyrrolo[2,3-c]pyridin-7(6H)-one (3A)

To a cooled (0–5 °C) solution of **2A** (3.63 g, 16.1 mmol) in dry THF (50 mL) under nitrogen was added sodium hydride (0.72 g of 60% dispersion in oil, 18.0 mmol) and stirred for 10 min. p-toluenesulfonyl chloride (3.3 g, 17.4 mmol) was then added portion-wise over 5 min, and the reaction mixture was allowed to warm to ambient temperature and stirred under nitrogen for 1 h. The reaction mixture was concentrated, diluted with ethyl acetate (50 mL) and washed with water (2 × 20 mL). The organic layer was concentrated and purified by silica gel column chromatography (2–20% EtOAc in hexanes) to provide 4-bromo-7-methoxy-1-tosyl-1H-pyrrolo[2,3-c]pyridine (**2A-a)** (4.9 g, 80% yield). MS (ESI^+^) m/z 381.1 (M + H)^+^.

4-bromo-7-methoxy-1-tosyl-1H-pyrrolo[2,3-c]pyridine (4.9 g, 12.8 mmol) in 1,4-dioxane (50 mL) and 4 M HCl in 1,4-dioxane (50 mL) were stirred at 65 °C for 5 h. The reaction mixture was then cooled to ambient temperature. The reaction mixture was filtered, rinsed with water (10 mL), and dried to provide compound **3A** (4.35 g, 92% yield) as a beige color solid. MS (ESI^+^) m/z 367.1 (M + H)^+^. ^1^H NMR (500 MHz, DMSO-d_6_) δ 11.50 (bs, 1H), 8.03 (d, *J* = 3.0 Hz, 1H), 7.94 (d, *J* = 8.0 Hz, 2H), 7.41 (d, *J* = 8.0 Hz, 2H), 7.35 (s, 1H), 6.59 (d, *J* = 3.5 Hz, 1H), 2.37 (s, 3H).

#### 4-Bromo-6-methyl-1-tosyl-1H-pyrrolo[2,3-c]pyridin-7(6H)-one (4A)

To a suspension of compound **3A** (4.34 g, 11.8 mmol) and cesium carbonate (5.35 g, 16.4 mmol) in dioxane (75 mL), iodomethane (3.9 g, 16.5 mmol) was added dropwise and stirred at ambient temperature for 4 h. The reaction mixture was concentrated, diluted with ethyl acetate (75 mL) and washed with water (2 × 25 mL). The organic layer was concentrated and purified by silica gel column chromatography (0–5% MeOH in CHCl_3_) to provide compound **4A** as white solid (3.6 g, 78% yield). MS (ESI^+^) m/z 380.8 (M + H)^+^.^1^ H NMR (500 MHz, CDCl_3_) δ 8.00 (d, *J* = 8.0 Hz, 2H), 7.93 (d,* J* = 3.5 Hz, 1H), 7.31 (d, *J* = 8.0 Hz, 2H), 7.17 (s, 1H), 6.51 (d, *J* = 3.5 Hz, 1H), 3.50 (s, 3H), 2.40 (s, 3H).

#### 6-Methyl-4-(4,4,5,5-tetramethyl-1,3,2-dioxaborolan-2-yl)-1-tosyl-1H-pyrrolo[2,3-c]pyridin-7(6H)-one (5A)

A mixture of compound **4A** (1.04 g, 2.6 mmol), 4,4,4′,4′,5,5,5′,5′-octamethyl-2,2′-bi(1,3,2-dioxaborolane) (0.80 g, 3.2 mmol), potassium acetate (0.50 g, 5.1 mmol), tris(dibenzylideneacetone)dipalladium(0) (60 mg, 0.065 mmol), and 2-dicyclohexylphosphino-2′,4′,6′-triisopropylbiphenyl (X-PHOS, 123 mg, 0.26 mmol) was degassed by vacuum and filled with nitrogen (repeated three times). Degassed dry 1,4-dioxane (20 mL) was added to the reaction mixture and heated under argon at 90 °C for 5 h. The consumption of starting material was monitored by TLC (1% MeOH in CHCl_3_). The reaction mixture was cooled to ambient temperature, concentrated, and partitioned between ethyl acetate (40 mL) and water (2 × 20 mL). The ethyl acetate layer was washed with brine (2 × 10 mL), dried over anhydrous Na_2_SO_4_, and concentrated. The residue was purified by silica gel column chromatography (10–80% ethyl acetate in hexanes) to provide compound **5A** as a waxy solid (5.4 g, 73% yield). MS (ESI^+^) m/z 429.1 (M + H)^+^. ^1^H NMR (500 MHz, DMSO-d_6_) δ 7.98 (d, *J* = 3.5 Hz, 1H), 7.90 (d, *J* = 8.0 Hz, 2H), 7.72 (s, 1H), 7.41 (d, *J* = 8.0 Hz, 2H), 6.81 (d, *J* = 3.5 Hz, 1H), 3.43 (s, 3H), 2.37 (s, 3H), 1.29 (s, 12H).

### Synthesis of pendant aryl bromides

#### 3-Bromo-2-(4-fluorophenoxy)pyridine (18-P)

To a solution of 4-fluorophenol (160 mg, 1.43 mmol) in dry DMF, 60% sodium hydride in mineral oil (60 mg, 1.5 mmol) was added portion-wise at ambient temperature and stirred for 10 min. 3-bromo-2-chloropyridine (276 mg, 1.43 mmol) was added to the reaction mixture and heated at 150 °C for 2 h. The reaction mixture was cooled, concentrated, diluted with ethyl acetate (30 mL), washed with water (10 mL), dried over Na_2_SO_4_, concentrated, and purified by silica gel column chromatography (0–5% methanol in CHCl_3_) to provide title compound (300 mg, 77%). MS (ESI^+^) m/z 267.9 (M + H)^+^. ^1^H NMR (500 MHz, CDCl_3_) 8.05 (dd*, J* = 1.5, 5.0 Hz, 1H), 7.93 (dd*, J* = 1.5, 7.5 Hz, 1H), 7.15–7.08 (m, 4H), 6.89 (dd*, J* = 5.0, 8.0 Hz, 1H).

#### 3-bromo-2-(3,4-difluorophenoxy)pyridine (81-P)

To a solution of 3,4-difluorophenol (500 mg, 3.85 mmol) in dry DMF, 60% sodium hydride in mineral oil (138 mg, 5.78 mmol) was added portion-wise at ambient temperature and stirred for 10 min. 3-bromo-2-chloropyridine (730 mg, 3.80 mmol) was added to the reaction mixture and heated at 150 °C for 2 h. The reaction mixture was cooled, concentrated and then diluted again with ethyl acetate (30 mL). Thereafter, the crude product was washed with water (10 mL), dried over Na_2_SO_4_, concentrated, and finally, purified by silica gel column chromatography (0–5% methanol in CHCl_3_) to provide the title compound as a colorless liquid at room temperature (1 g, 90.9% yield). ^1^H NMR (400 MHz, CDCl_3_) δ 8.07 (q, *J* = *2.1* Hz, 1H), 7.94 (dd,* J*_*1*_ = *7.9* Hz*, J*_*2*_ = *1.7* Hz*,* 1H), 7.19 (q*, J* = *9.2* Hz, 1H), 7.04 (dq*, J*_*1*_ = *10.8* Hz*, J*_*2*_ = *3.2* Hz*,* 1H), 6.93 (m, 2H).

#### 3-bromo-2-(3,5-difluorophenoxy)pyridine (82-P)

To a solution of 3,5-difluorophenol (500 mg, 3.85 mmol) in dry DMF, 60% sodium hydride in mineral oil (138 mg, 5.78 mmol) was added portion-wise at ambient temperature and stirred for 10 min. 3-bromo-2-chloropyridine (730 mg, 3.80 mmol) was added to the reaction mixture and heated at 150 °C for 2 h. The reaction mixture was cooled, concentrated and then diluted again with ethyl acetate (30 mL). Thereafter, the crude product was washed with water (10 mL), dried over Na_2_SO_4_, concentrated, and finally, purified by silica gel column chromatography (0–5% methanol in CHCl_3_) to provide the title compound as an ivory color solid substance (800 mg, 72.7% yield). ^1^H NMR (500 MHz, CDCl_3_) δ 8.12 (q, *J* = *2.1* Hz, 1H), 7.96 (dd*, J*_*1*_ = *7.6* Hz*, J*_*2*_ = *1.4* Hz*,* 1H), 6.98 (q*, J* = *4.1* Hz, 1H), 6.71 (m*,* 3H).

#### 3-bromo-2-((4-fluorophenyl)thio)pyridine (84-P)

To a solution of 4-fluorobenzenethiol (183 mg, 1.43 mmol) in dry DMF, Potassium carbonate (395 mg, 2.86 mmol) was added portion-wise at ambient temperature and stirred for 10 min. 3-bromo-2-chloropyridine (276 mg, 1.43 mmol) was added to the reaction mixture and heated at 140 °C for 2 h. The reaction mixture was cooled, concentrated and then diluted again with ethyl acetate (30 mL). Thereafter, the crude product was washed with water (10 mL), dried over Na_2_SO_4_, concentrated, and finally, purified by silica gel column chromatography (0–5% methanol in CHCl_3_) to provide the title compound as yellow semi liquid crystalline substance at room temperature (265 mg, 65.2% yield). ^1^H NMR (500 MHz, CDCl_3_) δ 8.24 (q, *J* = *2.1* Hz, 1H), 7.74 (dd*, J*_*1*_ = *8.3* Hz*, J*_*2*_ = *1.4* Hz*,* 1H), 7.54 (td*, J*_*1*_ = *6.2* Hz*, J*_*2*_ = *2.5* Hz*,* 2H), 7.13 (m*,* 2H), 6.89 (q*, J* = *4.1* Hz, 1H).

#### 3-bromo-N-(4-fluorophenyl)pyridin-2-amine (85-P)

To a solution of 4-fluoroaniline (159 mg, 1.43 mmol) in dry DMF, 60% sodium hydride in mineral oil (50 mg, 2.1 mmol) was added portion-wise at ambient temperature and stirred for 10 min. 3-bromo-2-chloropyridine (276 mg, 1.43 mmol) was added to the reaction mixture and heated at 150 °C for 2 h. The reaction mixture was cooled, concentrated and then diluted again with ethyl acetate (30 mL). Thereafter, the crude product was washed with water (10 mL), dried over Na_2_SO_4_, concentrated, and finally, purified by silica gel column chromatography (0–5% methanol in CHCl_3_) to provide the title compound as yellowish liquid crystalline material at room temperature (307 mg, 80.3% yield). ^1^H NMR (500 MHz, CDCl_3_) δ 8.12 (q, *J* = *2.1* Hz, 1H), 7.73 (dd*, J*_*1*_ = *7.9* Hz*, J*_*2*_ = *1.7* Hz*,* 1H), 7.54 (m*,* 2H), 7.04 (m*,* 2H), 6.92 (bs*,* 1H), 6.63 (q*, J* = *4.4* Hz, 1H).

#### 3-bromo-5-fluoro-2-(4-fluorophenoxy)pyridine (86-P)

To a solution of 4-fluorophenol (160 mg, 1.43 mmol) in dry DMF, 60% sodium hydride in mineral oil (50 mg, 2.1 mmol) was added portion-wise at ambient temperature and stirred for 10 min. 3-bromo-2-chloro-5-fluoropyridine (300 mg, 1.43 mmol) was added to the reaction mixture and heated at 150 °C for 2 h. The reaction mixture was cooled, concentrated and then diluted again with ethyl acetate (30 mL). Thereafter, the crude product was washed with water (10 mL), dried over Na_2_SO_4_, concentrated, and finally, purified by silica gel column chromatography (0–5% methanol in CHCl_3_) to provide the title compound as an ivory solid substance at room temperature (368 mg, 90% yield). ^1^H NMR (500 MHz, CDCl_3_) δ 8.10 (d, *J* = *2.8* Hz, 1H), 7.50 (d*, J* = *2.8* Hz*,* 1H), 7.11 (m*,* 2H), 7.04 (m*,* 2H).

#### General procedure for Suzuki coupling of 5A and aryl bromide

Aryl bromide pendant (0.10–0.20 mmol) (**6a-f)**, anhydrous K_2_CO_3_ (3.0 eq.), meCgPPh (0.06 eq.) and Pd_2_dba_3_ (0.03 eq.) were added to a three-neck flask and the reaction mixture was degassed by vacuum and back-filled with nitrogen (repeated three times). 1.1 M solution of boronic acid pinacol ester intermediate **5A** (1.5 eq.) in degassed 4:1 dioxane-water mixture was then added to the reaction mixture and stirred at 65 °C for 2–4 h. Reaction was monitored using TLC analysis (80% EtOAc in hexanes or 5% MeOH in CHCl_3_) till the disappearance of boronic acid pinacol ester intermediate (2–4 h). The reaction mixture was diluted with ethyl acetate and washed with water. The organic layer was dried over Na_2_SO_4,_ evaporated to dryness and purified using preparative TLC (5% MeOH in CHCl_3_).

#### 4-(2-(4-Fluorophenoxy)pyridin-3-yl)-6-methyl-1-tosyl-1H-pyrrolo[2,3-c]pyridin-7(6H)-one (18A-CPL)

Was obtained in 47% yield following general procedure above. MS (ESI^+^) m/z 490.1 (M + H)^+^. ^1^H NMR (500 MHz, DMSO-d_6_) δ 7.97 (dd, *J* = 3.5, 5.5 Hz, 1H), 7.99–7.95 (m, 3H), 7.85 (dd, *J* = 2.5, 7.5 Hz, 1H), 7.64 (s, 1H), 7.42 (d, *J* = 8.5 Hz, 2H), 7.24–7.16 (m, 5H), 6.63 (d, *J* = 3.5 Hz, 1H), 3.47 (s, 3H), 2.38 (s, 3H).

#### 4-(2-(3,4-difluorophenoxy)pyridin-3-yl)-6-methyl-1-tosyl-1,6-dihydro-7H-pyrrolo[2,3-c]pyridin-7-one (81A-CPL)

The title compound was obtained in 70% yield following the general Suzuki coupling procedure as described above. ^1^H NMR (500 MHz, CDCl_3_) δ 8.17 (q, *J* = *2.3* Hz, 1H), 8.06 (d, *J* = *9.0* Hz, 2H), 7.93 (d, *J* = *3.4* Hz, 1H), 7.71 (dd, *J*_*1*_ = *7.6* Hz*, J*_*2*_ = *2.1* Hz, 1H), 7.32 (d, *J* = *8.3* Hz, 2H), 7.15 (m, 3H), 6.96 (m, 1H), 6.82 (m, 1H), 6.39 (d, *J* = *3.4* Hz, 1H), 3.58 (s, 3H), 2.41 (s, 3H).

#### 4-(2-(3,5-difluorophenoxy)pyridin-3-yl)-6-methyl-1-tosyl-1,6-dihydro-7H-pyrrolo[2,3-c]pyridin-7-one (82A-CPL)

The title compound was obtained in 74% yield following the general Suzuki coupling procedure as described above. ^1^H NMR (500 MHz, CDCl_3_) δ 8.22 (q, *J* = *2.3* Hz, 1H), 8.05 (d, *J* = *8.3* Hz, 2H), 7.93 (d, *J* = *3.4* Hz, 1H), 7.74 (dd, *J*_*1*_ = *7.2* Hz*, J*_*2*_ = *1.7* Hz, 1H), 7.33 (d, *J* = *8.3* Hz, 2H), 7.17 (dd, *J*_*1*_ = *7.2* Hz*, J*_*2*_ = *5.2* Hz, 1H), 7.15 (s, 1H), 6.64 (qd, *J*_*1*_ = *4.8* Hz*, J*_*2*_ = *2.8* Hz, 3H), 6.38 (d, *J* = *3.4* Hz, 1H), 3.58 (s, 3H), 2.41 (s, 3H).

#### 4-(2-((4-fluorophenyl)thio)pyridin-3-yl)-6-methyl-1-tosyl-1,6-dihydro-7H-pyrrolo[2,3-c]pyridin-7-one (84A-CPL).

The title compound was obtained in 69% yield following the general Suzuki coupling procedure as described above. ^1^H NMR (500 MHz, CDCl_3_) δ 8.36 (q, *J* = *2.3* Hz, 1H), 8.08 (d, *J* = *9.0* Hz, 2H), 7.92 (d, *J* = *3.4* Hz, 1H), 7.46 (td, *J*_*1*_ = *6.7* Hz*, J*_*2*_ = *4.4* Hz, 1H), 7.30 (dd, *J*_*1*_ = *10.7* Hz*, J*_*2*_ = *2.4* Hz*,* 2H), 7.10 (m, 3H), 7.02 (m, 3H), 6.24 (d, *J* = *3.4* Hz, 1H), 3.58 (s, 3H), 2.43 (s, 3H).

#### 4-(2-((4-fluorophenyl)amino)pyridin-3-yl)-6-methyl-1-tosyl-1,6-dihydro-7H-pyrrolo[2,3-c]pyridin-7-one (85A-CPL)

The title compound was obtained in 42% yield following the general Suzuki coupling procedure as described above. ^1^H NMR (400 MHz, CDCl_3_) δ 8.25 (dd, *J*_*1*_ = *5.0* Hz*, J*_*2*_ = *1.9* Hz, 1H), 8.09 (d, *J* = *8.4* Hz, 2H), 7.91 (d, *J* = *3.6* Hz, 1H), 7.38 (m, 5H), 7.08 (s, 1H), 6.98 (t, *J* = *8.7* Hz, 2H), 6.81 (dd, *J*_*1*_ = *7.4* Hz*, J*_*2*_ = *5.0* Hz, 1H), 6.27 (d, *J* = *3.4* Hz,1H), 6.15 (s, 1H), 3.58 (s, 3H), 2.42 (s, 3H).

#### 4-(5-fluoro-2-(4-fluorophenoxy)pyridin-3-yl)-6-methyl-1-tosyl-1,6-dihydro-7H-pyrrolo[2,3-c]pyridin-7-one (86A-CPL)

The title compound was obtained in 61% yield following the general Suzuki coupling procedure as described above. ^1^H NMR (400 MHz, CDCl_3_) δ 8.16 (d, *J* = *2.9* Hz, 1H), 8.05 (d, *J* = *8.5* Hz, 2H), 7.91 (d, *J* = *3.6* Hz, 1H), 7.33 (d, *J* = *8.0* Hz, 2H), 7.23 (d, *J* = *3.0*^1^, 1H), 7.07 (m, 5H), 6.22 (d, *J* = *3.6*^1^, 1H), 3.56 (s, 3H), 2.42 (s, 3H).

### General procedure for detosylation

Product of above coupling in 5 mL methanol–water (4:1) and 3 eq. of K_2_CO_3_ were stirred at 85 °C for 2–3 h. The reaction was monitored using TLC (4% MeOH in CHCl_3_) till the disappearance of starting material (step g product). The reaction mixture was then evaporated to dryness. The crude was suspended in 10 mL water and extracted with 10% MeOH–CHCl_3_. The organic layer was dried over Na_2_SO_4,_ evaporated to dryness and purified using preparative TLC (4% MeOH in CHCl_3_, two repeated runs).

#### 4-(2-(4-fluorophenoxy)pyridin-3-yl)-6-methyl-1H-pyrrolo[2,3-c]pyridin-7(6H)-one (18A)

Was obtained as white solid in 96% yield following general procedure step h. MS (ESI^+^) m/z 336.1 (M + H)^+^.^1^H NMR (500 MHz, DMSO-d_6_) δ 12.09 (bs, 1H), 8.11 (dd,* J* = 1.5 Hz, 4.5 Hz, 1H), 7.89 (d, *J* = 7.0 Hz, 1H), 7.42 (s, 1H), 7.29 (t, *J* = 2.5 Hz, 1H), 7.24–7.14 (m, 5H), 6.29 (s, 1H), 3.57 (s, 3H). ^13^C NMR (125 MHz, DMSO-d_6_) 160.3, 154.1, 149.7, 145.8, 140.3, 129.7, 129.3, 126.8, 123.3, 122.9, 120.7, 119.1, 116.0, 115.9, 109.8, 102.7, 35.6. HPLC: t_*R*_ = 7.18 min, 98.4%.

#### 4-(2-(3,4-difluorophenoxy)pyridin-3-yl)-6-methyl-1,6-dihydro-7H-pyrrolo[2,3-c]pyridin-7-one (81A)

The title compound was obtained as white solid with 80% yield following the general procedure as mentioned in step h. MS (ESI^+^) m/z 354.1 (M + H)^+^.^1^H NMR (500 MHz, CDCl_3_) δ 9.80 (bs, 1H), 8.17 (q, *J* = *2.3* Hz, 1H), 7.84 (dd, *J*_*1*_ = *7.2* Hz*, J*_*2*_ = *1.7* Hz, 1H), 7.27 (t, *J* = *2.8* Hz, 1H), 7.15 (m, 3H), 6.99 (m, 1H), 6.85 (m, 1H), 6.36 (t, *J* = *2.4* Hz, 1H), 3.71 (s, 3H). HPLC: t_*R*_ = 7.454 min, 93.546%.

#### 4-(2-(3,5-difluorophenoxy)pyridin-3-yl)-6-methyl-1,6-dihydro-7H-pyrrolo[2,3-c]pyridin-7-one (82A)

The title compound was obtained as white waxy solid with 82% yield following the general procedure as mentioned in step h. MS (ESI^+^) m/z 353.1 (M^+^). ^1^H NMR (500 MHz, CDCl_3_) δ 10.01 (bs, 1H), 8.22 (q, *J* = *2.1* Hz, 1H), 7.87 (dd, *J*_*1*_ = *7.2* Hz*, J*_*2*_ = *1.7* Hz, 1H), 7.29 (m, 1H), 7.19 (dd, *J*_*1*_ = *6.9* Hz*, J*_*2*_ = *4.8* Hz, 1H), 7.12 (s, 1H), 6.67 (td, *J*_*1*_ = *7.4* Hz*, J*_*2*_ = *2.5* Hz, 2H), 6.62 (m, 1H), 6.35 (d, *J* = *2.1* Hz, 1H), 3.70 (s, 3H). HPLC: t_*R*_ = 7.641 min, 97.941%.

#### 4-(2-((4-fluorophenyl)thio)pyridin-3-yl)-6-methyl-1,6-dihydro-7H-pyrrolo[2,3-c]pyridin-7-one (84A)

The title compound was obtained as white solid with 89% yield following the general procedure as mentioned in step h. MS (ESI^+^) m/z 352.17 (M + H)^+^. ^1^H NMR (500 MHz, CDCl_3_) δ 10.34 (bs, 1H), 8.37 (q, *J* = *2.1* Hz, 1H), 7.58 (dd, *J*_*1*_ = *7.6* Hz*, J*_*2*_ = *2.1* Hz, 1H), 7.43 (qd, *J*_*1*_ = *5.7* Hz*, J*_*2*_ = *3.1* Hz, 2H), 7.30 (t, *J* = *2.4* Hz, 1H), 7.12 (q, *J* = *4.1* Hz, 1H), 7.05 (m, 3H), 6.22 (t, *J* = *2.4* Hz, 1H), 3.73 (s, 3H). HPLC: t_*R*_ = 7.469 min, 99.136%.

#### 4-(2-((4-fluorophenyl)amino)pyridin-3-yl)-6-methyl-1,6-dihydro-7H-pyrrolo[2,3-c]pyridin-7-one (85A)

The title compound was obtained as light yellow waxy solid with 81% yield following the general procedure as mentioned in step h. ^1^H NMR (500 MHz, CDCl_3_) δ 10.41 (bs, 1H), 8.26 (dd, *J*_*1*_ = *5.0* Hz*, J*_*2*_ = *1.9* Hz, 1H), 7.49 (dd, *J*_*1*_ = *7.3* Hz*, J*_*2*_ = *1.9* Hz, 1H), 7.42 (m, 2H), 7.31 (t, *J* = *2.8* Hz, 1H), 7.03 (s, 1H), 6.96 (m, 2H), 6.83 (dd, *J*_*1*_ = *7.3* Hz*, J*_*2*_ = *4.9* Hz, 1H), 6.40 (s, 1H), 6.27 (t, *J* = *2.5* Hz, 1H), 3.73 (s, 3H). HPLC: t_*R*_ = 5.426 min, 99.380%.

#### 4-(5-fluoro-2-(4-fluorophenoxy)pyridin-3-yl)-6-methyl-1,6-dihydro-7H-pyrrolo[2,3-c]pyridin-7-one (86A)

The title compound was obtained as white waxy substance with 65% yield following the general procedure as mentioned in step h. MS (ESI^+^) m/z 353.4 (M^+^). ^1^H NMR (500 MHz, CDCl_3_) δ 10.24 (s, 1H), 8.17 (d, *J* = 2.8 Hz, 1H), 7.36 (d, *J* = 2.8 Hz, 1H), 7.27 (t, *J* = 2.8 Hz, 1H), 7.07 (m, 5H), 6.18 (t, *J* = 2.4 Hz, 1H), 3.70 (s, 3H). HPLC: t_*R*_ = 7.802 min, 94.018%.

### Synthesis of “B”-series compounds


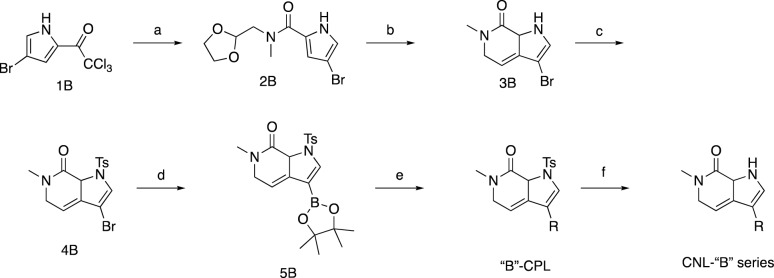


**Reagents and conditions**: *(a) (1,3-dioxolan-2-yl)-N-methylmethanamine, DMF, RT; (b) MsOH, 60 °C; (c) NaH, pTsCl, THF, 0 °C; (d) bis(pinacolato)diboron, KOAc, Pd*_*2*_*dba*_*3*_*, X-phos, 1,4-dioxane, 95 °C; (e) (18-P, 81-P, 82-P, 84-P, 85-P, 86-P), K*_*2*_*CO*_*3*_*, Pd*_*2*_*dba*_*3*_*, meCgPPh, 1,4-dioxane-water (4:1), 65 °C; (f) K*_*2*_*CO*_*3*_*, methanol–water, 85 °C.*

#### N-((1,3-Dioxolan-2-yl)methyl)-4-bromo-N-methyl-1H-pyrrole-2-carboxamide (2B)

To a solution of 1-(4-bromo-1H-pyrrol-2-yl)-2,2,2-trichloroethanone (**1B**) (0.94 g, 3.2 mmol) in dry DMF (2.5 mL), (1,3-dioxolan-2-yl)-*N*-methylmethanamine (0.45 g, 3.8 mmol) was added and stirred at ambient temperature for 18 h. Another batch of (1,3-dioxolan-2-yl)-*N*-methylmethanamine (0.45 g, 3.8 mmol) was added and heated to 70 °C for 5 h. The reaction mixture was evaporated, diluted with ethyl acetate (30 mL) and washed with water (2 × 15 mL). The organic phase was dried over Na_2_SO_4_, evaporated and purified by silica gel column chromatography (20–100% ethyl acetate in hexane) to afford the title compound (0.84 g, 90%) as beige solid. MS (ESI^+^) m/z 289.0 (M + H)^+^.^1^H NMR (500 MHz, DMSO-*d*_6_) δ 11.78 (bs, 1H), 7.03–7.01 (m, 1H), 6.65 (s, 1H), 5.00 (s, 1H), 3.95–3.82 (m, 4H), 3.61 (s, 2H), 3.22 (s, 3H).

#### 3-Bromo-6-methyl-1H-pyrrolo[2,3-c]pyridin-7(6H)-one (3B)

A solution of **2B** (370 mg, 1.3 mmol) in methanesulfonic acid (2.5 mL) was stirred at 60 °C for 24 h. The reaction mixture was cooled and poured into 3 M NaOH solution (15 mL) and stirred for 30 min. The resultant suspension was filtered and purified by preparative TLC (4% methanol in CHCl_3_) to provide title compound (80 mg, 27%) as white solid. MS (ESI^+^) 226.9 m/z (M + H)^+^. ^1^H NMR (500 MHz, DMSO-*d*_6_) δ 12.39 (bs, 1H), 7.47 (s, 1H), 7.28 (d*, J* = 6.5 Hz, 1H), 6.33 (d*, J* = 7.5 Hz, 1H), 3.50 (s, 3H).

#### 3-Bromo-6-methyl-1-tosyl-1H-pyrrolo[2,3-c]pyridin-7(6H)-one (4B)

To a cooled solution of **3B** (150 mg, 0.66 mmol) in dry THF (5 mL), sodium hydride (32 mg of 60% dispersion in oil, 0.80 mmol) was added portion-wise. After 10 min, tosyl chloride (160 mg, 0.84 mmol) was added to the solution in portions and stirred for 1 h at RT. The reaction mixture was diluted with water (30 mL) and extracted with ethyl acetate (30 mL). The organic phase was dried over Na_2_SO_4_ and dried to provide the title compound (250 mg, 98%) as white solid. MS (ESI^+^) m/z 381.0 (M + H)^+^.^1^H NMR (500 MHz, DMSO-*d*_*6*_) δ 8.18 (s, 1H), 8.00 (d,* J* = 8.5 Hz, 2H), 7.53 (d*, J* = 7.0 Hz, 1H), 7.43 (d*, J* = 8.0 Hz, 2H), 6.35 (d*, J* = 7.0 Hz, 1H), 3.41 (s, 3H), 2.38 (s, 3H).

#### 6-Methyl-3-(4,4,5,5-tetramethyl-1,3,2-dioxaborolan-2-yl)-1-tosyl-1H-pyrrolo[2,3-c]pyridin-7(6H)-one (5B): 4B

(127 mg, 0.35 mmol), 4,4,4′,4′,5,5,5′,5′-octamethyl-2,2′-bi(1,3,2-dioxaborolane) (0.42 g, 1.65 mmol), dried potassium acetate (74 mg, 0.75 mmol), Xphos (17 mg, 0.04 mmol) and Pd_2_dba_3_ (8 mg, 0.02 mmol) were taken in an RBF and degassed by vacuum and filled with nitrogen (repeated three times). To the reaction mixture, dry 1, 4-dioxane (2.0 mL) was added and stirred at 95 °C for 3 h. The reaction was diluted with ethyl acetate (30 mL) and washed with water (2 × 15 mL) and brine (2 × 10 mL). The organic phase was evaporated and purified by preparative TLC (1% methanol in chloroform) to provide the target compound (120 mg, 80%) and proto-dehalogenated by-product. It was used for the next step without further purification. A small quantity of material was further purified for analytical sample preparation. MS (ESI^+^) m/z 429.1 (M + H)^+^. ^1^H NMR (400 MHz, CDCl_3_) δ 8.26 (s, 1H), 8.04 (d*, J* = *8.4* Hz, 2H), 7.30 (d*, J* = *8.4* Hz, 2H), 7.01 (d*, J* = *7.2* Hz, 1H), 6.77 (d*, J* = *7.2* Hz, 1H), 3.51 (s, 3H), 2.39 (s, 3H), 1.35 (s, 12H).

### General procedure for step-e (Suzuki coupling of pendant Aryl Bromide and 5B)

Aryl bromide pendant (**18-P, 81-P, 82-P, 84-P, 85-P, 86-P**) (0.10–0.20 mmol), anhydrous K_2_CO_3_ (3.0 eq.), meCgPPh (0.06 eq.) and Pd_2_dba_3_ (0.03 eq.) were added to a three-neck flask and the reaction mixture was degassed by vacuum and back-filled with nitrogen (repeated three times). 1.1 M solution of boronic acid pinacol ester intermediate **5B** (1.5 eq.) in degassed 4:1 1,4-dioxane-water mixture was added to the reaction mixture and stirred at 65 °C for 2–4 h. Reaction was monitored using TLC analysis (80% EtOAc in hexanes or 5% MeOH in CHCl_3_) till the disappearance of boronic acid pinacol ester intermediate (2–4 h). The reaction mixture was diluted with ethyl acetate and washed with water. The organic layer was dried over Na_2_SO_4,_ evaporated to dryness and purified using preparative TLC (5% MeOH in CHCl_3_).

#### 3-(2-(3,4-difluorophenoxy)pyridin-3-yl)-6-methyl-1-tosyl-1,6-dihydro-7H-pyrrolo[2,3-c]pyridin-7-one (81B-CPL)

^1^H NMR (400 MHz, CDCl_3_) δ 8.17 (dd, *J*_*1*_ = *4.8* Hz*, J*_*2*_ = *1.8* Hz*,* 1H), 8.05 (dd*, J*_*1*_ = *20.1* Hz*, J*_*2*_ = *8.4* Hz*,* 2H), 7.86 (m*,* 1H), 7.32 (m*,* 2H), 7.16 (m*,* 2H), 7.03 (m*,* 2H), 6.89 (m*,* 1H), 6.45 (m*,* 1H), 3.52 (s, 3H), 2.41 (s, 3H).

#### 3-(2-(3,5-difluorophenoxy)pyridin-3-yl)-6-methyl-1-tosyl-1,6-dihydro-7H-pyrrolo[2,3-c]pyridin-7-one (82B-CPL)

^1^H NMR (400 MHz, CDCl_3_) δ 8.27 (s, 1H), 8.07 (m*, 3*H), 7.88 (d*, J* = *3.4* Hz, 1H), 7.31 (m*,* 3H), 7.03 (m*,* 2H), 6.77 (d*, J* = *6.9* Hz, 1H), 6.44 (m*,* 1H), 6.36 (d, *J* = *7.0* Hz, 1H), 3.52 (s, 3H), 2.41 (s, 3H).

#### 3-(2-((4-fluorophenyl)thio)pyridin-3-yl)-6-methyl-1-tosyl-1,6-dihydro-7H-pyrrolo[2,3-c]pyridin-7-one (84B-CPL)

^1^H NMR (400 MHz, CDCl_3_) δ 8.36 (dd, *J*_*1*_ = *4.8* Hz*, J*_*2*_ = *1.8* Hz*,* 1H), 8.10 (m*, 3*H), 7.52 (dd*, J*_*1*_ = *7.6* Hz*, J*_*2*_ = *1.8* Hz*,* 1H), 7.46 (m*,* 2H), 7.35 (d*, J* = 8.1 Hz, 2H), 7.09 (m*, 4*H), 6.26 (d, *J* = 7.1 Hz, 1H), 3.55 (s, 3H), 2.42 (s, 3H).

#### 3-(2-((4-fluorophenyl)amino)pyridin-3-yl)-6-methyl-1-tosyl-1,6-dihydro-7H-pyrrolo[2,3-c]pyridin-7-one (85B-CPL)

^1^H NMR (500 MHz, CDCl_3_) δ 8.45 (t, *J* = *2.6* Hz*,* 1H), 8.08 (dd, *J*_*1*_ = *11.7* Hz*, J*_*2*_ = *8.3* Hz*,* 3H), 7.75 (dd*, J*_*1*_ = *7.6* Hz*, J*_*2*_ = *2.1* Hz*,* 1H), 7.35 (q*, J* = *4.1* Hz, 3H), 7.05 (d*, J* = *6.9* Hz, 1H), 6.26 (d, *J* = *7.6* Hz, 1H), 3.54 (s, 3H), 2.43 (s, 3H).

#### 3-(5-fluoro-2-(4-fluorophenoxy)pyridin-3-yl)-6-methyl-1-tosyl-1,6-dihydro-7H-pyrrolo[2,3-c]pyridin-7-one (86B-CPL)

^1^H NMR (500 MHz, CDCl_3_) δ 8.16 (d, *J* = *2.8* Hz*,* 1H), 8.07 (d, *J* = *6.2* Hz*, 3H)*, 7.34 (d, *J* = 8.3 Hz, 2H), 7.31 (s*,* 1H), 7.07 (m*,* 5H), 6.22 (d, *J* = *6.9* Hz, 1H), 3.53 (s, 3H), 2.42 (s, 3H).

### General procedure for step-f (detosylation)

Product of step e in 5 mL methanol–water (4:1) and 3 eq. of K_2_CO_3_ were stirred at 85 °C for 2–3 h. The reaction was monitored using TLC (4% MeOH in CHCl_3_) till the disappearance of starting material (step g product). The reaction mixture was then evaporated to dryness. The crude was suspended in 10 mL water and extracted with 10% MeOH-CHCl_3_. The organic layer was dried over Na_2_SO_4,_ evaporated to dryness and purified using preparative TLC (4% MeOH in CHCl_3_).

#### 3-[2-(4-fluorophenoxy)pyridin-3-yl]-6-methyl-1,6-dihydro-7H-pyrrolo[2,3-c]pyridin-7-one(18B)

Was obtained as white solid in 36% yield from 5B following general Suzuki coupling and detosylation procedures. MS (ESI +) m/z 336.1 (M + H) + 0.1HNMR (500 MHz, DMSO-d6)δ12.33 (bs, 1H),8.02–7.96 (m, 2H), 7.68 (d, J = 3.0 Hz, 1H), 7.26–7.18 (m, 6H),6.68 (d, J = 7.0 Hz, 1H),3.52 (s, 3H).HPLC:tR = 7.28 min, 98.9%

#### 3-(2-(3,4-difluorophenoxy)pyridin-3-yl)-6-methyl-1,6-dihydro-7H-pyrrolo[2,3-c]pyridin-7-one (81B)

The title compound was obtained as greyish solid in 80% yield following the general detosylation procedure as mentioned in step-f. MS (ESI^+^) m/z 354.14 (M + H)^+^. ^1^H NMR (500 MHz, CDCl_3_) δ 10.17 (bs, 1H), 8.08 (q, *J* = 2.3 Hz, 1H), 7.90 (dd*, J*_*1*_ = *7.6* Hz*, J*_*2*_ = *2.1* Hz*,* 1H), 7.63 (d*, J* = *2.8* Hz*,* 1H), 7.14 (m*,* 2H), 7.01 (m*,* 2H), 6.87 (m*,* 1H), 6.70 (d*, J* = *7.6* Hz*,* 1H), 3.66 (s, 3H). HPLC: t_*R*_ = 7.534 min, 97.354%.

#### 3-(2-(3,5-difluorophenoxy)pyridin-3-yl)-6-methyl-1,6-dihydro-7H-pyrrolo[2,3-c]pyridin-7-one (82B)

The title compound was obtained as white solid in 76% yield following the the general detosylation procedure as mentioned in step-f. MS (ESI^+^) m/z 354.14 (M + H)^+^. ^1^H NMR (500 MHz, CDCl_3_) δ 10.60 (bs, 1H), 8.14 (q, *J* = 2.1 Hz, 1H), 7.93 (dd*, J*_*1*_ = *7.2* Hz*, J*_*2*_ = *1.7* Hz*,* 1H), 7.61 (d*, J* = *2.8* Hz*,* 1H), 7.18 (q*, J* = *4.1* Hz*,* 1H), 7.02 (d*, J* = 7.6 Hz, 1H), 6.69 (m*,* 3H), 6.62 (tt*, J*_*1*_ = *9.1* Hz*, J*_*2*_ = *2.4* Hz*,* 1H), 3.68 (s, 3H). HPLC: t_*R*_ = 7.641 min, 97.941%.

#### 3-(2-((4-fluorophenyl)thio)pyridin-3-yl)-6-methyl-1,6-dihydro-7H-pyrrolo[2,3-c]pyridin-7-one (84B)

The title compound was obtained as white solid in 96% yield following the the general detosylation procedure as mentioned in step-f. MS (ESI^+^) m/z 352.30 (M + H)^+^. ^1^H NMR (500 MHz, CDCl_3_) δ 10.49 (bs, 1H), 8.31 (q, *J* = *2.3* Hz, 1H), 7.56 (dd*, J*_*1*_ = *7.6* Hz*, J*_*2*_ = *2.1* Hz*,* 1H), 7.52 (d*, J* = *2.8* Hz*,* 1H), 7.47 (m*,* 2H), 7.07 (m*,* 3H), 7.01 (d*, J* = *6.9* Hz, 1H), 6.51 (d*, J* = *6.9* Hz*,* 1H), 3.70 (s, 3H). HPLC: t_*R*_ = 7.528 min, 95.010%.

#### 3-(2-((4-fluorophenyl)amino)pyridin-3-yl)-6-methyl-1,6-dihydro-7H-pyrrolo[2,3-c]pyridin-7-one (85B)

The title compound was obtained as white solid in 96% yield following the the general detosylation procedure as mentioned in step-f. MS (ESI^+^) m/z 335.3 (M + H)^+^. ^1^H NMR (500 MHz, CDCl_3_) δ 10.79 (bs, 1H), 8.21 (q, *J* = *2.3* Hz, 1H), 7.48 (m*,* 5H), 6.98 (m*,* 3H), 6.83 (dd*, J*_*1*_ = *7.2* Hz*, J*_*2*_ = *5.2* Hz*,* 1H), 6.60 (m*,* 1H), 6.50 (d*, J* = *6.9* Hz, 1H), 3.69 (s, 3H). HPLC: t_*R*_ = 5.384 min, 94.261%.

#### 3-(5-fluoro-2-(4-fluorophenoxy)pyridin-3-yl)-6-methyl-1,6-dihydro-7H-pyrrolo[2,3-c]pyridin-7-one (86B)

The title compound was obtained as white solid in 96% yield following the general detosylation procedure as mentioned in step-f. MS (ESI^+^) m/z 353.4 (M^+^). ^1^H NMR (500 MHz, CDCl_3_) δ 10.70 (bs, 1H), 8.09 (d, *J* = *2.8* Hz, 1H), 7.55 (d*, J* = *2.8* Hz, 1H), 7.36 (d*, J* = *3.4* Hz*,* 1H), 7.08 (m*,* 4H), 6.99 (d*, J* = *6.9* Hz, 1H), 6.46 (d*, J* = 7.6 Hz, 1H), 3.67 (s, 3H). HPLC: t_*R*_ = 7.833 min, 97.276%.

### Supplementary Information

Supplementary Information.

## Data Availability

The authors declare that the data supporting the findings of this study are available within the paper and its Supplementary Information files. Should any raw data files be needed in another format, they are available from the corresponding author upon reasonable request. Patient cell lines are available upon request with an appropriate MTA and approval from MGH IRB.
