# Supplementary material for: Epigenetic modulation through BET bromodomain inhibitors as a novel therapeutic strategy for progranulin-deficient frontotemporal dementia
Source: Sci Rep. 2024 Apr 20;14:9064. doi: 10.1038/s41598-024-59110-7 (PMC11032351; doi:10.1038/s41598-024-59110-7)
Supplement: Supplementary file 1 — Supplementary Information. [file 41598_2024_59110_MOESM1_ESM.docx]

**Supplementary Information**

**
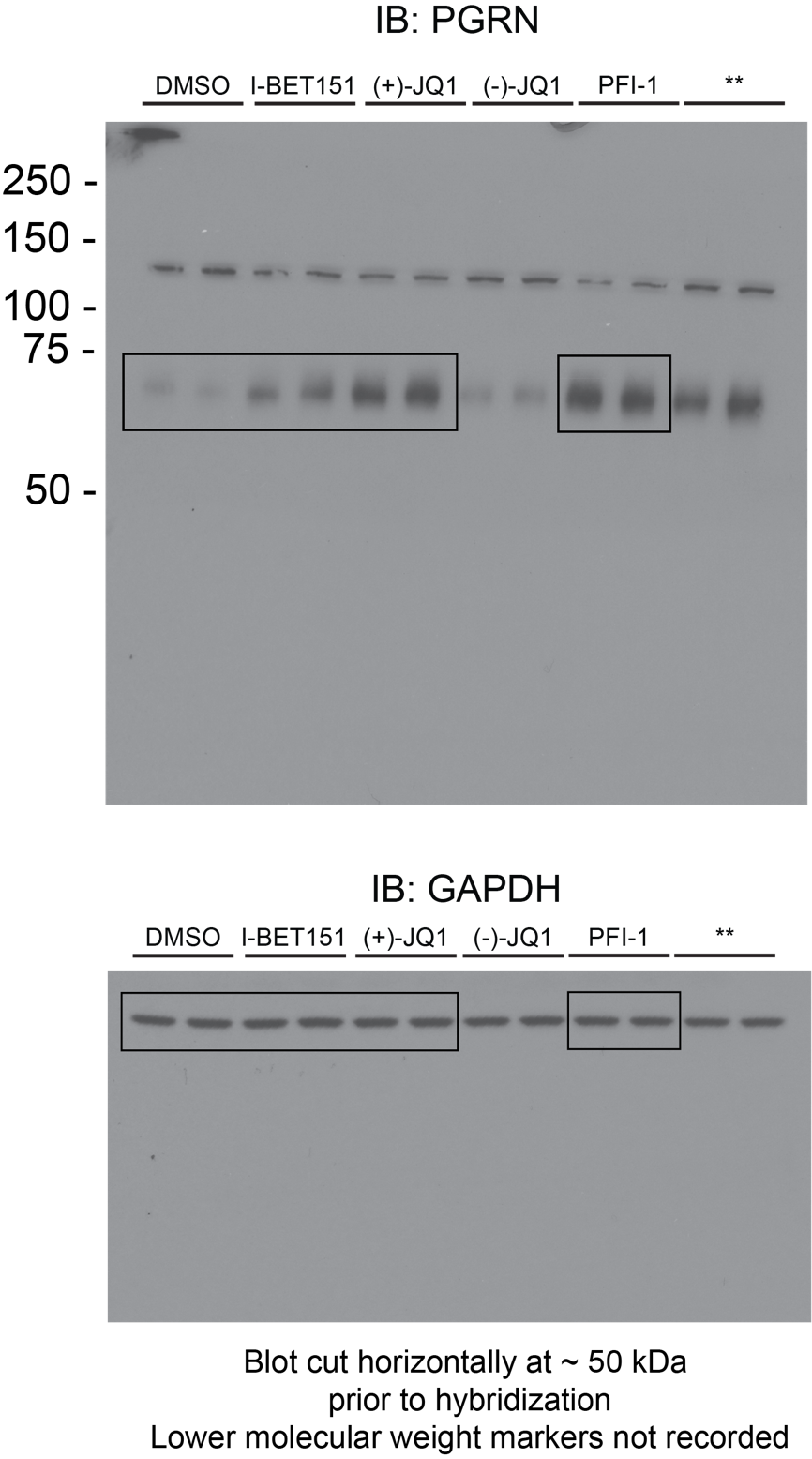
**

**Supplemental Figure 1.** BET inhibitors (2.5 µM I-BET151, 1 µM (+)-JQ1, 10 µM PFI-1) can enhance PGRN in neural progenitor cells after 24 hours while 1 µM inactive control (-)-JQ1 is unable to do so. ** indicates lanes not relevant for current study. GAPDH blot was split horizontally at ~ 50 kDa prior to immunoblotting for another immunoassay not related to this study. Boxed areas indicate data shown in Figure 1.

**
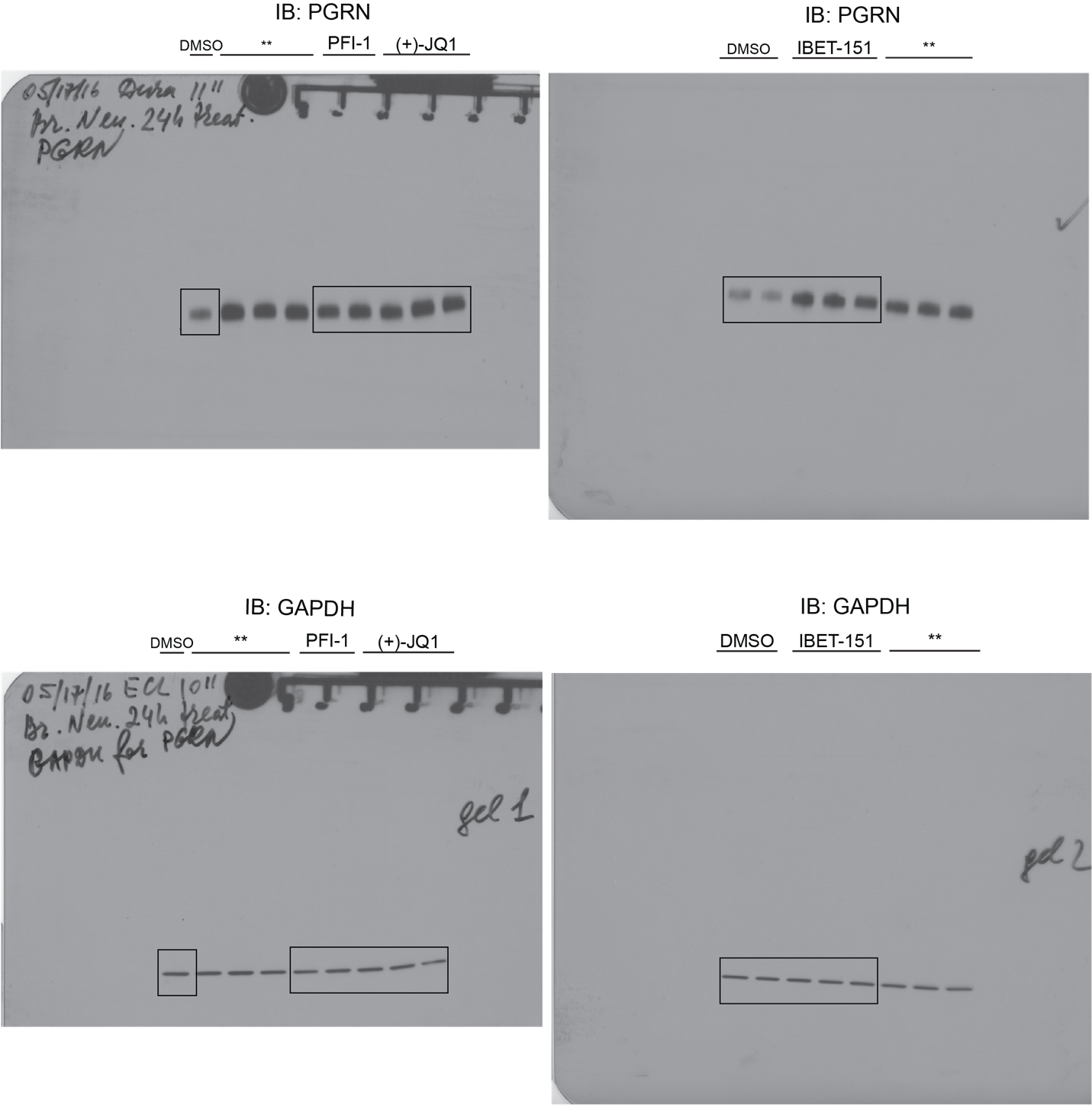
**

**Supplemental Figure 2.** BET inhibitors (2.5 µM I-BET151, 1 µM (+)-JQ1, 10 µM PFI-1) can enhance PGRN expression in neurons after 24 hours. ** indicates lanes not relevant for current study. Boxed areas indicate data show in Figure 1. No separate molecular weight standard was run due to antibody specificity and prior validation of molecular weight.

**
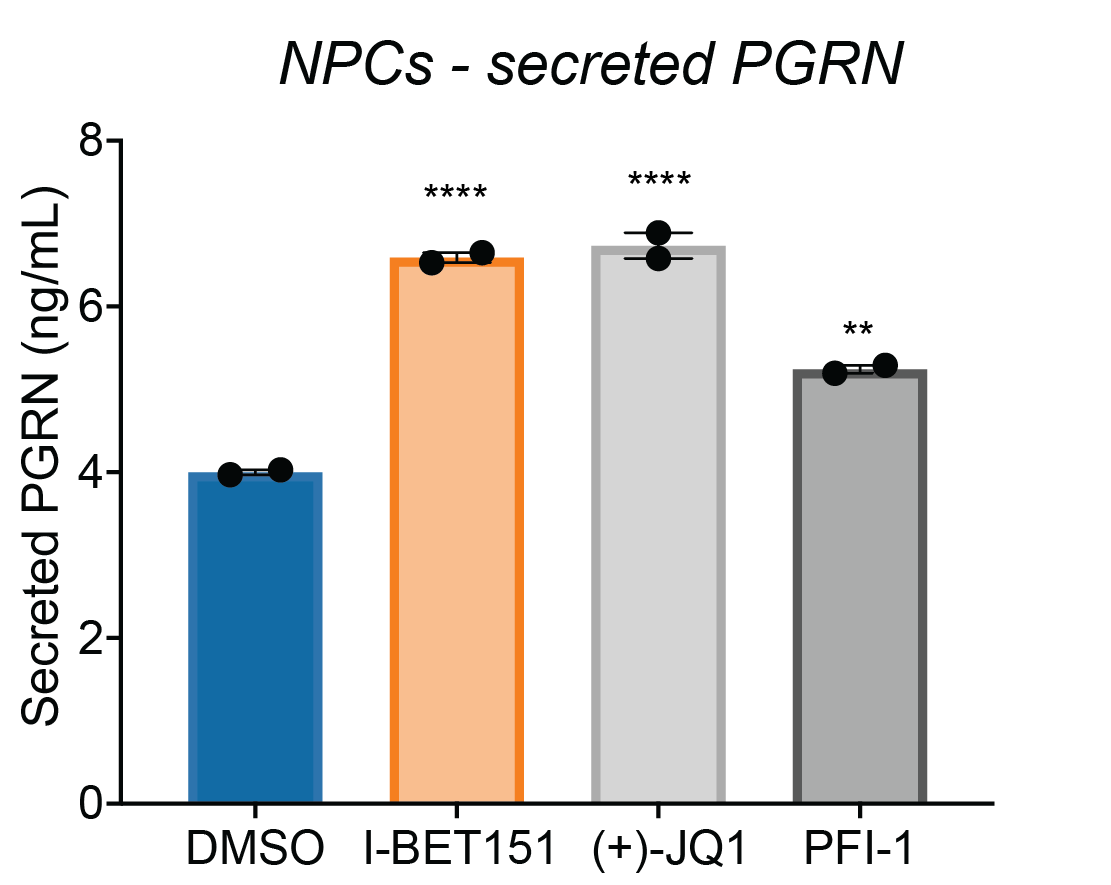
**

**Supplemental Figure 3. BET inhibitors I-BET151 (10 µM), (+)-JQ1 (1 µM), and PFI-1 (10 µM) enhance extracellular progranulin protein levels in NPCs.** Significance determined by Dunnett’s Multiple Comparison, * p <0.05, ** p <0.01, *** p <0.001, **** p <0.0001

**
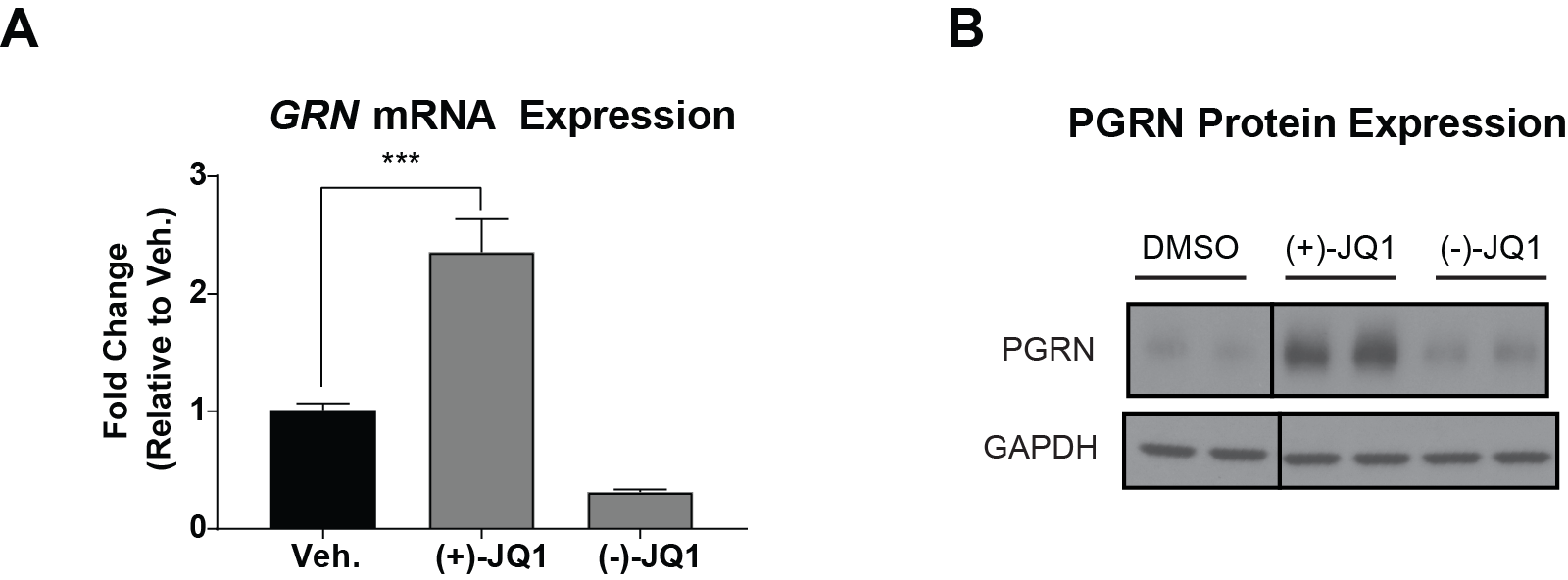
**

**Supplemental Figure 4.** (+)-JQ1 (1 µM), but not the inactive enantiomer (-)-JQ1 (1 µM), is able to enhance GRN mRNA (A) and PGRN protein levels (B) in NPCs after 24 hours. Data for (B) taken from Supplemental Figure 1. Significance determined by unpaired t-test. *** p <0.001


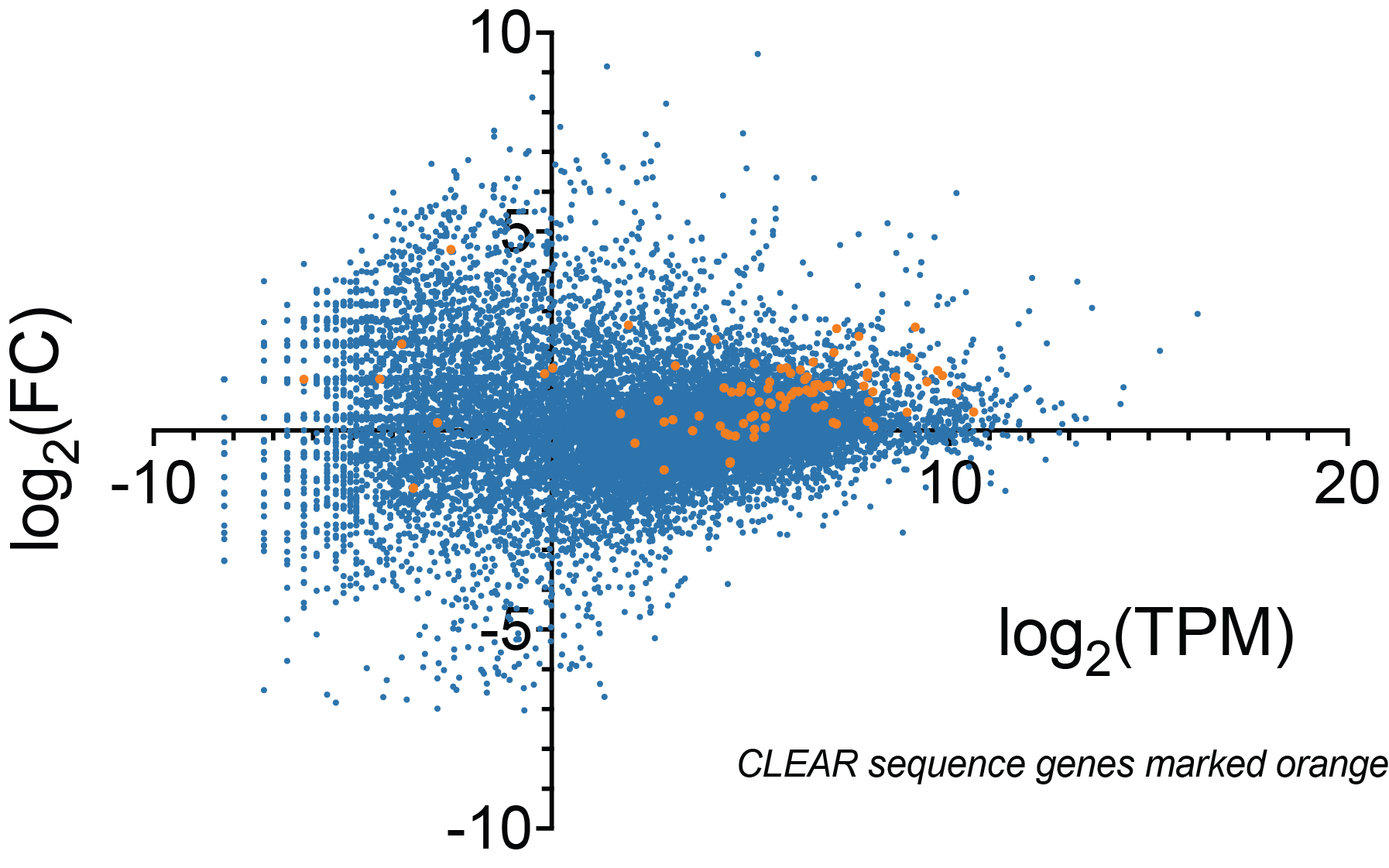


**Supplemental Figure 5.** RNA-seq data of human dermal fibroblasts treated with 1 µM mivebresib for 24 hours. Genes marked in orange are lysosomal genes as identified in Sardiello et al. (2009), Table S1.^42^


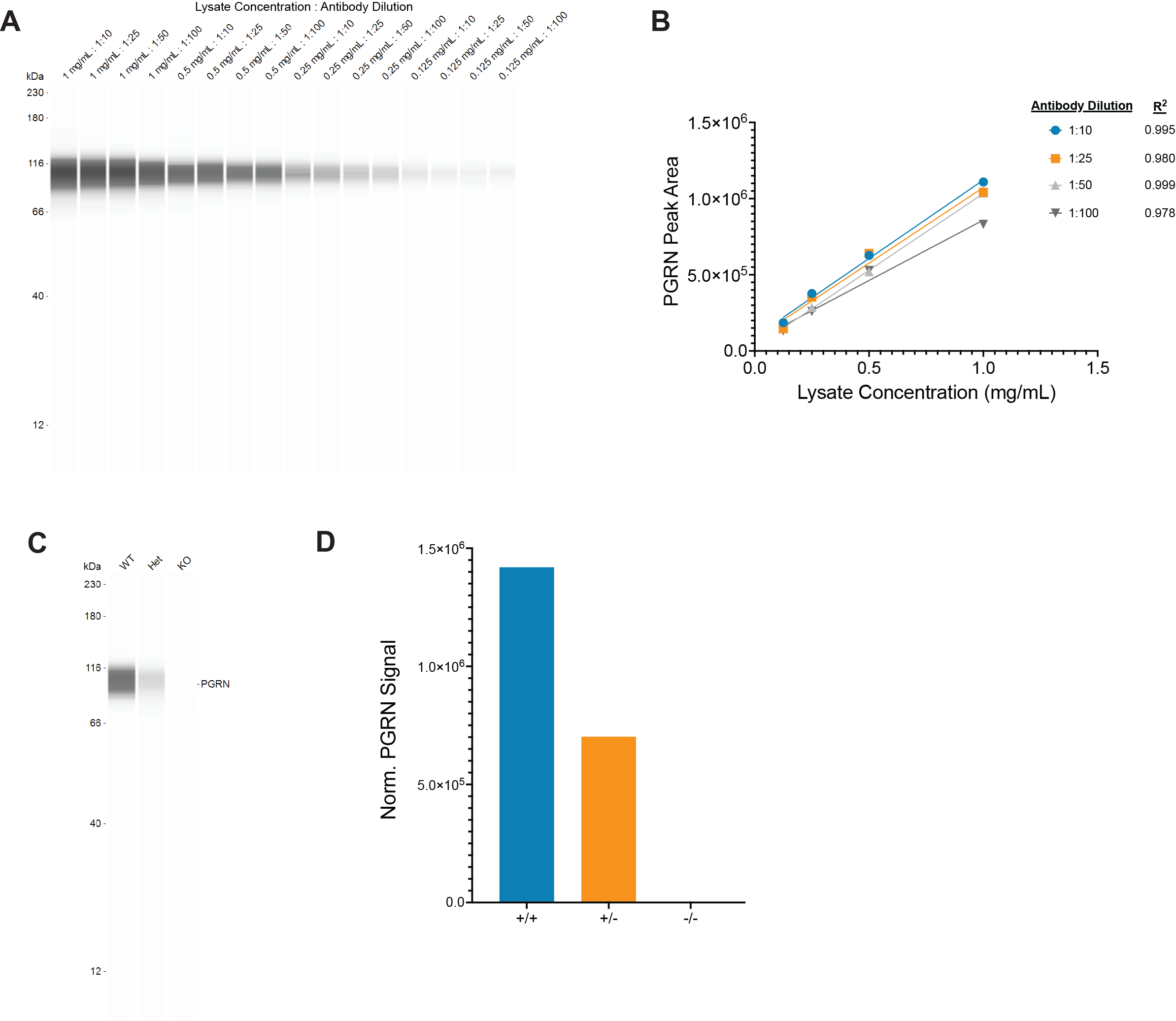


**Supplemental Figure 6.** Validation of Jess automated western blotting for the quantification of PGRN. (A) PGRN can be visualized across a wide range of lysate and antibody concentrations. (B) Quantification of data from (A) showing linearity of signal response. (C) iPSCs bearing a heterozygous and homozygous GRN R493X mutation show decreased expression of PGRN. (D) Quantification of (C).

**
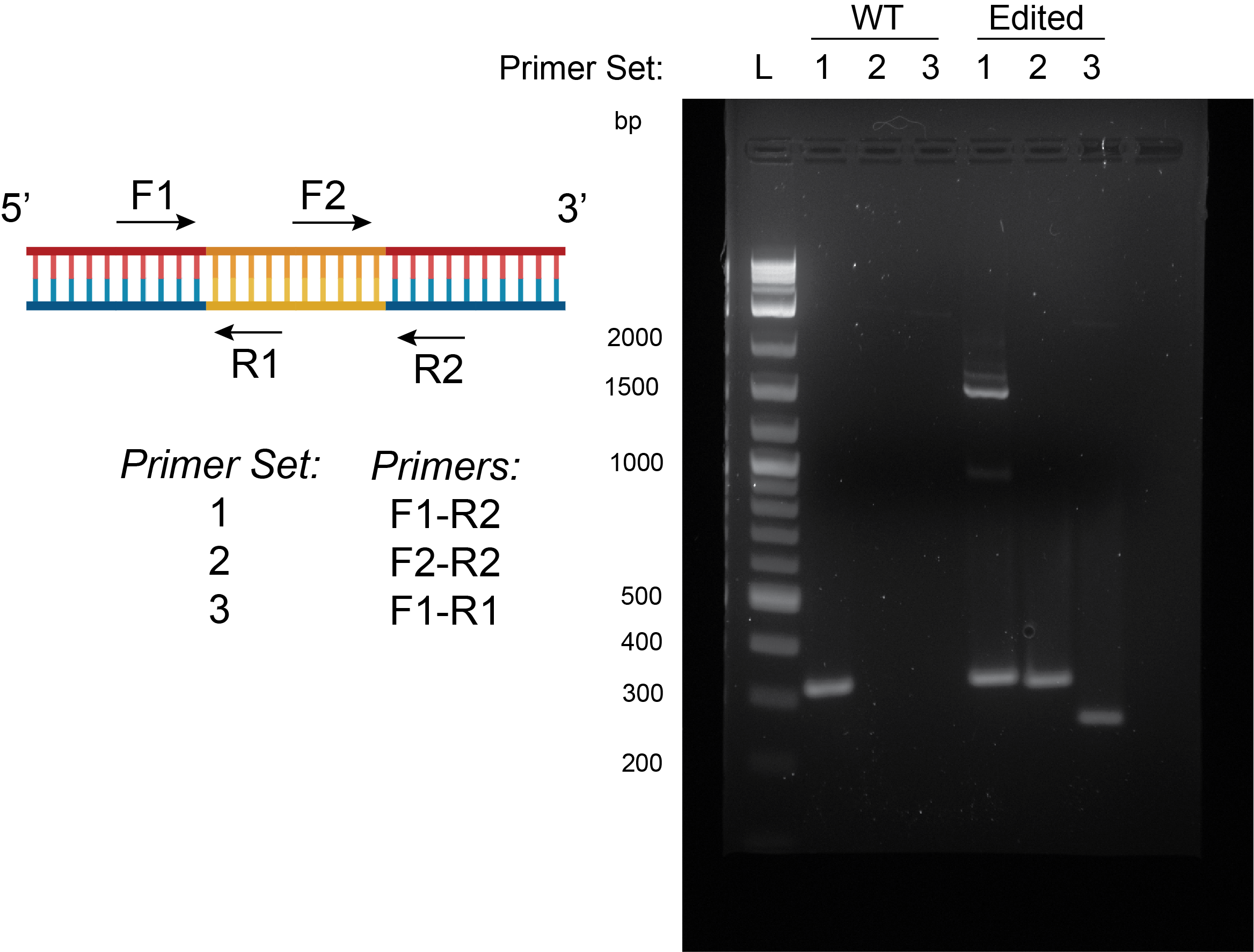
**

**Supplemental Figure 7. HMC3 PCR validation.** Primer pairs were used to amplify genomic DNA from either wild-type or edited lines. Expected amplicon sizes are as follows: Primer Set 1: ~1400 bp (with insert), ~300 bp without insert. Primer set 2: ~300 bp. Primer set 3: ~230 bp. “L” indicates ladder.

**
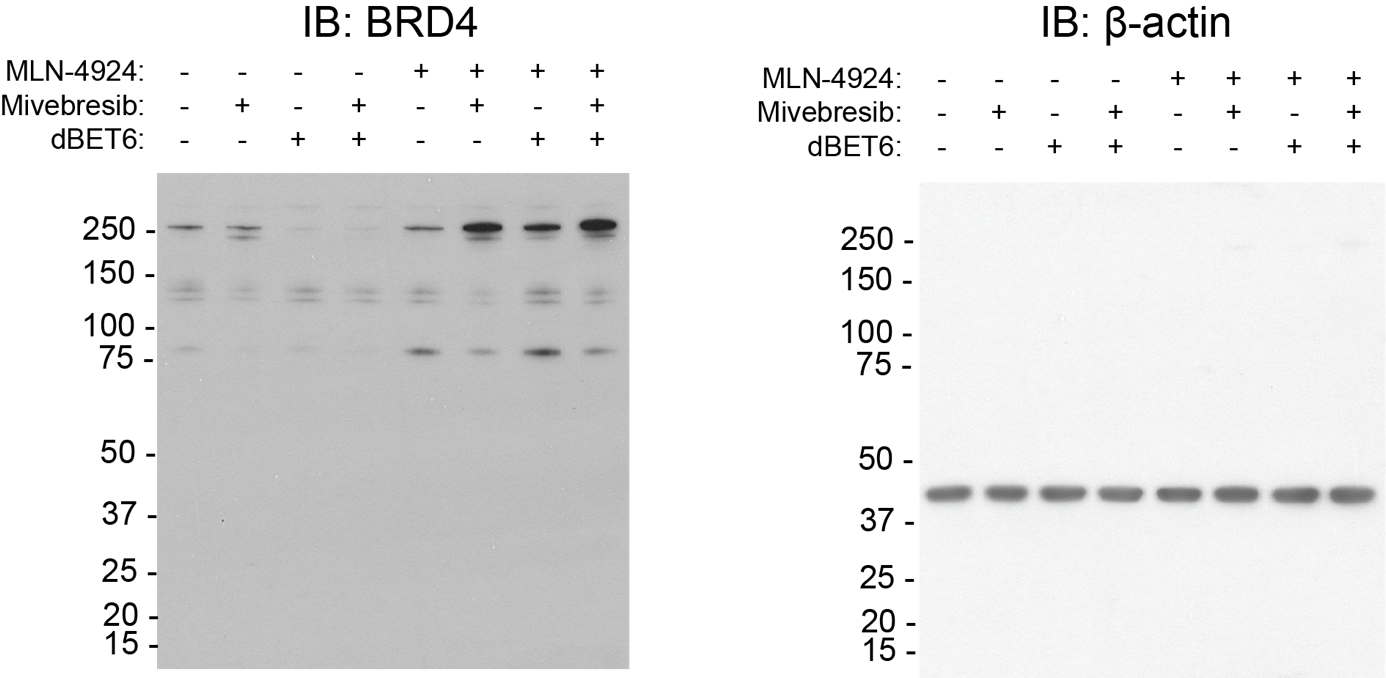
**

**Supplemental Figure 8.** Immunoblots against BRD4 and β-actin for NPCs treated with co-treatments of MLN-4924 (1 µM), mivebresib (1 µM), and dBET6 (300 nM).


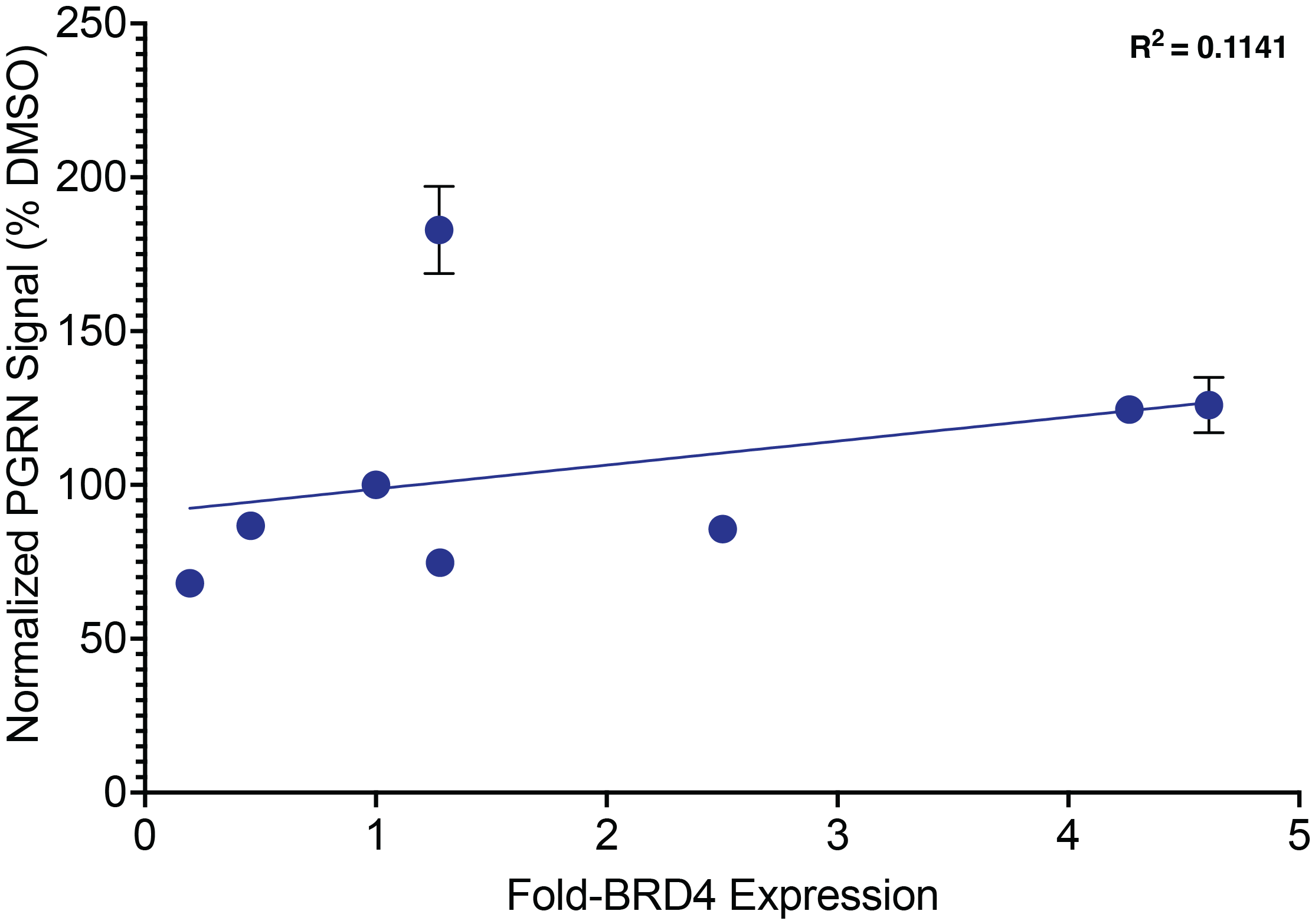


**Supplemental Figure 9.** BRD4 protein levels in NPCs (normalized to DMSO) do not strongly correlate with PGRN protein levels. Data from experiment described in Figure 3C-3E.


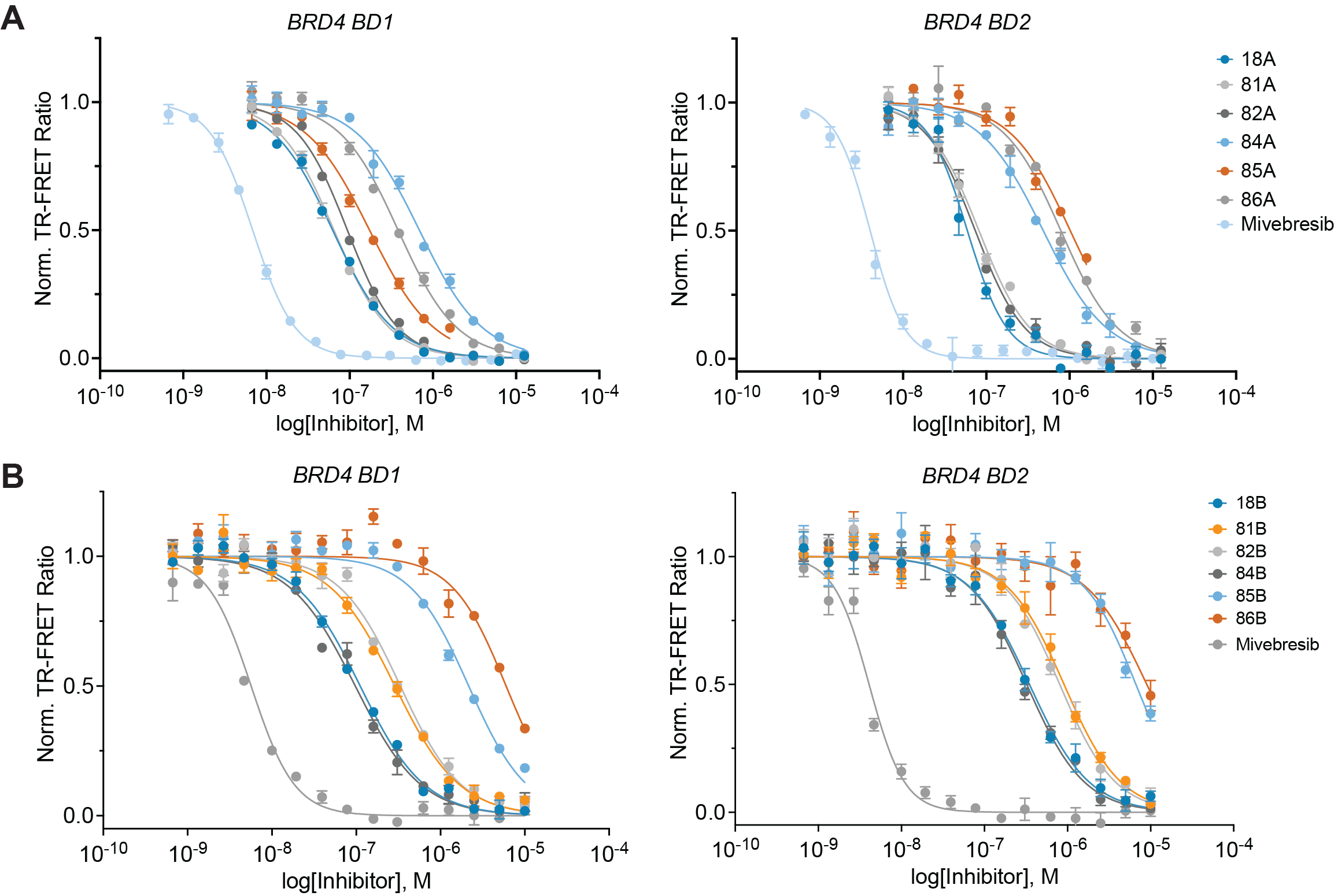


**Supplemental Figure 10.** (A-B) Analogs can engage GST-tagged, recombinantly expressed individual bromodomains of BRD4 with similar potencies. Data shown are from n=3 biological replicates ± SEM.


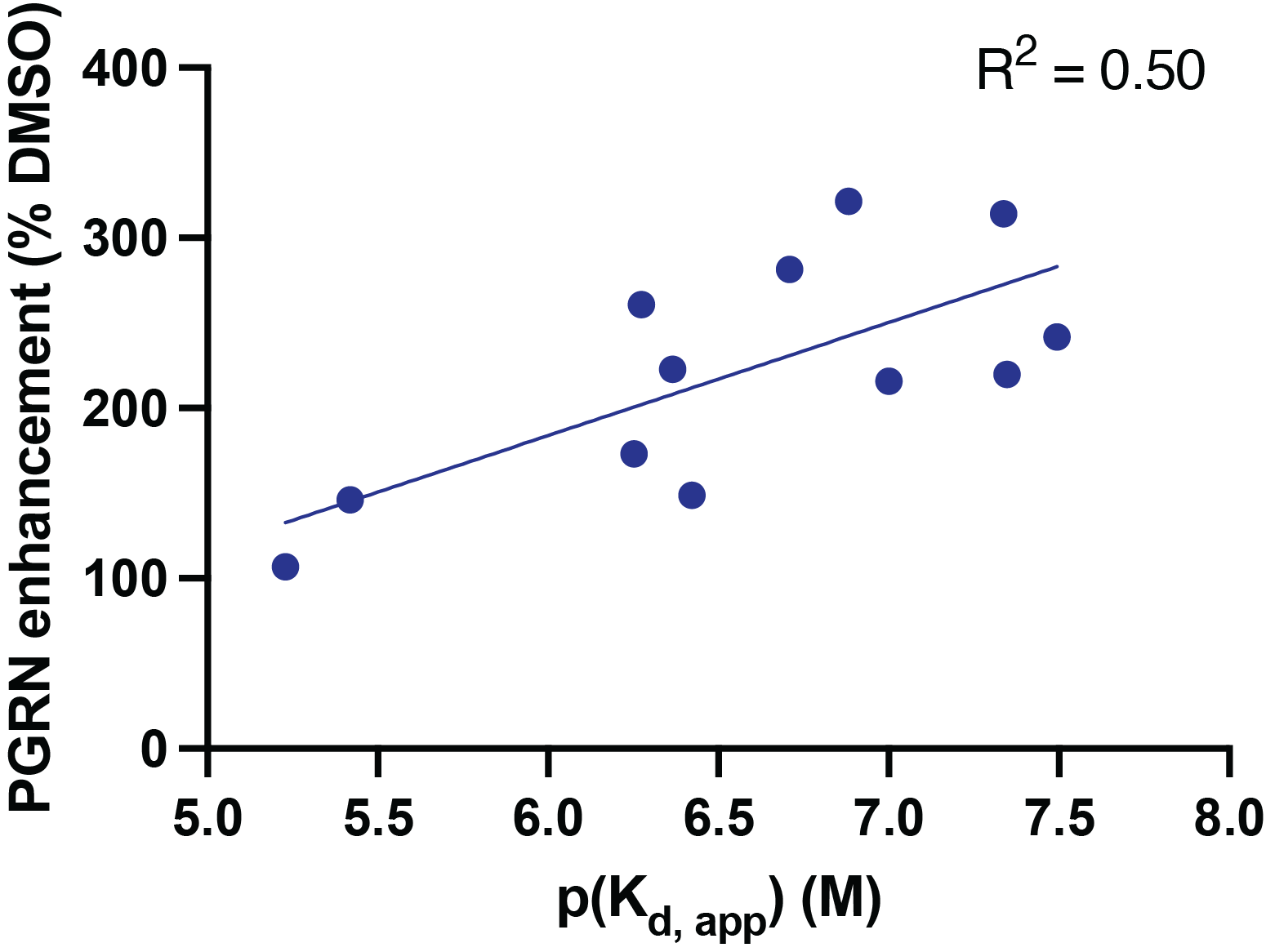


**Supplemental Figure 11.** BET inhibitor target engagement with BRD4 derived from NPC lysates correlates with PGRN enhancement capacity in NPCs. Data re-plotted from experiments described in Table 1 and Figure 6F-6G.

**
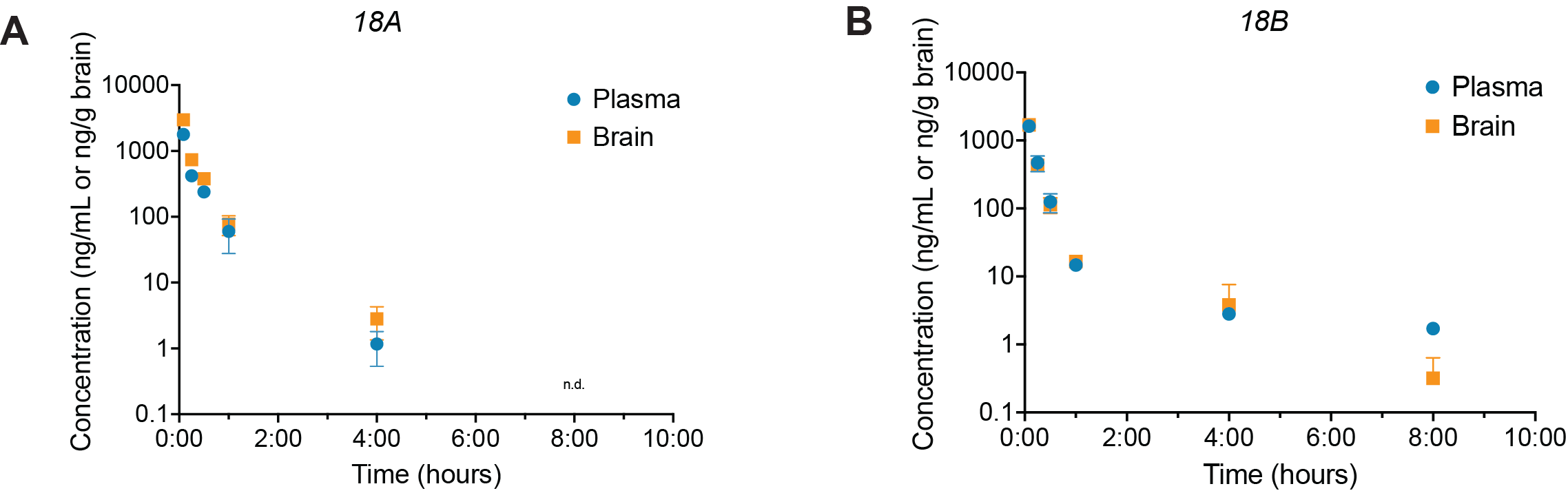
**

**Supplemental Figure 12.** Pharmacokinetic profile of 18A (A) and 18B (B) injected intravenously (5 mg/kg) in CD-1 mice.

Supplemental Table 1. Full list of compounds tested in *GRN* enhancer screen. Data from experiment described in Figure 1B.

| **Compound** | **Dose (µM)** | ***GRN* Fold-change (Norm. to GAPDH) ± Std. Error** |
| --- | --- | --- |
| CHIR-99021 | 10 | 0.79 ± 0.06 |
| JNJ16259685 | 10 | 1.00 ± 0.10 |
| Lanosoprazole | 10 | 1.37 ± 0.23 |
| (-)-Eseroline fumarate | 10 | 1.10 ± 0 |
| (-)-Eseroline fumarate | 25 | 0.79 ± 0.46 |
| Eserine | 25 | 0.73 ± 0.15 |
| BG05-I-9* | 10 | 3.48 ± 0.85 |
| Panobinostat | 1 | 3.08 ± 0.35 |
| Cpd-60 | 10 | 1.39 ± 0.18 |
| Bizine | 10 | 0.92 ± 0.12 |
| Z220650400 | 10 | 1.17 ± 0.10 |
| Roflumilast | 10 | 1.01 ± 0.09 |
| PF-2545920 | 10 | 1.29 ± 0.16 |
| Ruxolitinib | 10 | 1.26 ± 0.04 |
| EPZ005687 | 10 | 1.18 ± 0.10 |
| Bromosporine | 10 | 3.44 ± 0.25 |
| GSK-J4 | 10 | 0.76 ± 0.03 |
| PFI-1 | 10 | 2.54 ± 0.43 |
| GSK-LSD1 | 10 | 0.96 ± 0.11 |
| ACY-1215 | 10 | 3.63 ± 0.57 |
| I-BET151 | 10 | 3.71 ± 0.22 |
| PCI-24781 | 1 | 3.68 ± 0.27 |
| BIX01294 | 1 | 0.80 ± 0.04 |
| GSK1070916 | 1 | 1.45 ± 0 |
| CNL-000552* | 10 | 0.92 ± 0.16 |
| CNL-000509* | 10 | 0.76 ± 0.17 |
| CNL-000487* | 10 | 1.21 ± 0.05 |
| CNL-000558* | 10 | 0.90 ± 0.05 |

* In-house synthesized compounds targeting various epigenetic regulators with currently undisclosed targets.

**Supplemental Table 2.** Alterations of lysosomal genes induced by mivebresib in human dermal fibroblasts. List from Sardiello et al. (2009), Table S1.

| Gene symbol | Log2(fold-change) | Q-value |
| --- | --- | --- |
| CTSH | 2.652 | 2.10E-09 |
| CTSK | 2.602 | 9.77E-64 |
| MCOLN1 | 2.564 | 5.62E-86 |
| NEU1 | 2.366 | 1.83E-71 |
| FUCA1 | 2.294 | 2.22E-37 |
| TMEM74 | 2.173 | 1.64E-01 |
| CREG1 | 1.959 | 6.72E-75 |
| NPC2 | 1.822 | 1.06E-44 |
| ASAH1 | 1.724 | 1.41E-28 |
| MANBA | 1.687 | 1.10E-40 |
| ACP5 | 1.631 | 2.41E-08 |
| OSTM1 | 1.559 | 1.58E-17 |
| CTSF | 1.580 | 1.01E-30 |
| CTSS | 1.574 | 1.76E-04 |
| NPC1 | 1.530 | 1.77E-48 |
| PSAP | 1.511 | 1.72E-38 |
| MPO | 1.298 | 6.05E-01 |
| SCPEP1 | 1.441 | 2.21E-33 |
| LGMN | 1.455 | 2.33E-29 |
| HPSE | 1.426 | 3.18E-04 |
| CTSD | 1.383 | 1.93E-27 |
| TPP1 | 1.361 | 2.24E-25 |
| CLCN7 | 1.349 | 2.02E-37 |
| CTSA | 1.344 | 2.62E-26 |
| GAA | 1.285 | 1.36E-23 |
| SLC17A5 | 1.220 | 2.25E-28 |
| LAMP1 | 1.227 | 1.15E-18 |
| IDS | 1.156 | 2.31E-30 |
| GNS | 1.166 | 4.59E-32 |
| IFI30 | 1.145 | 7.99E-15 |
| HEXA | 1.135 | 4.80E-16 |
| AGA | 1.111 | 4.88E-12 |
| HEXB | 1.117 | 2.67E-23 |
| IDUA | 1.057 | 4.92E-16 |
| LITAF | 1.022 | 1.97E-17 |
| LAMP2 | 1.082 | 5.64E-29 |
| SGSH | 0.983 | 4.75E-17 |
| CTNS | 1.077 | 8.63E-11 |
| RNASET2 | 1.008 | 1.76E-08 |
| NAGLU | 0.982 | 9.05E-16 |
| SLC36A1 | 0.968 | 1.14E-12 |
| ARSA | 0.958 | 1.53E-18 |
| ABCA2 | 0.969 | 2.39E-15 |
| PCYOX1 | 0.963 | 5.47E-18 |
| GBA | 0.975 | 3.56E-13 |
| CTSB | 0.947 | 1.51E-13 |
| HGSNAT | 0.896 | 2.32E-16 |
| CLN5 | 0.974 | 4.57E-14 |
| MAN2B2 | 0.870 | 1.64E-12 |
| CPVL | 4.550 | 6.33E-02 |
| ABCB9 | 0.753 | 4.80E-05 |
| DNASE2 | 0.759 | 2.22E-08 |
| GM2A | 0.722 | 1.97E-09 |
| ARSB | 0.697 | 2.40E-07 |
| SMPD1 | 0.723 | 1.50E-09 |
| GLB1 | 0.633 | 6.89E-06 |
| MAN2B1 | 0.586 | 5.75E-07 |
| GALNS | 0.664 | 3.03E-06 |
| LIPA | 0.574 | 1.84E-06 |
| CD63 | 0.465 | 1.49E-03 |
| LAPTM4A | 0.460 | 3.44E-05 |
| ARSG | 0.426 | 6.36E-02 |
| NEU4 | 1.289 | 8.29E-01 |
| SIAE | 0.365 | 2.44E-02 |
| GLA | 0.381 | 1.72E-02 |
| EPDR1 | 0.350 | 1.66E-01 |
| NAAA | 0.334 | 2.65E-02 |
| GALC | 0.272 | 1.19E-01 |
| CTSC | 0.179 | 3.19E-01 |
| HYAL1 | 0.217 | 3.05E-01 |
| CD68 | 0.237 | 1.63E-01 |
| LAPTM5 | 0.199 | 9.14E-01 |
| SCARB2 | 0.201 | 5.37E-02 |
| NCSTN | 0.166 | 2.66E-01 |
| LMBRD1 | 0.119 | 5.60E-01 |
| CTSZ | 0.095 | 4.91E-01 |
| ACP2 | 0.071 | 7.27E-01 |
| NAGA | 0.034 | 8.62E-01 |
| CTBS | -0.006 | 9.76E-01 |
| MFSD8 | -0.317 | 1.33E-01 |
| ENTPD4 | -0.066 | 6.61E-01 |
| CLN3 | -0.112 | 6.76E-01 |
| GUSB | -0.134 | 4.53E-01 |
| HYAL2 | -0.168 | 2.83E-01 |
| TMEM92 | -1.444 | 4.38E-01 |
| GGH | -0.787 | 4.73E-06 |
| PPT1 | -0.819 | 5.25E-09 |
| PPT2 | -0.987 | 4.01E-09 |
| TMEM92 | -1.444 | 4.38E-01 |

**Supplemental Table 3**. Transcription factor enrichment analysis of upregulated lysosomal genes using ChEA3 (Keenan et al. (2019)). Top 10 results are shown.

| **TF** | **Intersect** | **Set length** | **FET p-value** | **FDR** | **Odds Ratio** |
| --- | --- | --- | --- | --- | --- |
| TFEB | 12 | 745 | 1.037E-5 | 0.0023 | 5.351 |
| IRF8 | 7 | 238 | 1.5E-5 | 0.0023 | 9.905 |
| ESRRB | 12 | 1195 | 7.37E-4 | 0.0621 | 3.316 |
| MITF | 32 | 5140 | 0.001188 | 0.0621 | 2.048 |
| LYL1 | 8 | 633 | 0.001209 | 0.0621 | 4.184 |
| CTCF | 12 | 1270 | 0.001213 | 0.0621 | 3.118 |
| GATA2 | 10 | 1017 | 0.002125 | 0.0932 | 3.246 |
| E2F1 | 22 | 3465 | 0.003775 | 0.116 | 2.089 |
| BACH1 | 11 | 1298 | 0.004049 | 0.116 | 2.794 |
| ERG | 13 | 1681 | 0.00423 | 0.116 | 2.548 |

**Supplemental Table 4.** Calculated K_D_ and K_D,app_ values (with 95% confidence interval) for compounds against recombinant, GST-tagged BRD4-BD1 and BRD4-BD2.

| Compound | Recombinant BRD4(BD1), *K*_D_ (nM) | Recombinant BRD4(BD2), *K*_D_ (nM) |
| --- | --- | --- |
| 18A | 28 (26, 30) | 24 (22,27) |
| 81A | 29 (27, 31) | 34 (30, 37) |
| 82A | 41 (39, 43) | 30 (27, 34) |
| 84A | 303 (271, 339) | 199 (176, 226) |
| 85A | 76 (68, 85) | 421 (360, 503) |
| 86A | 168 (153, 184) | 324 (281, 375) |
| 18B | 46 (43, 49) | 158 (139, 180) |
| 81B | 126 (111, 145) | 375 (326, 431) |
| 82B | 147 (131, 165) | 332 (283, 390) |
| 84B | 43 (38, 49) | 133 (112, 159) |
| 85B | 951 (839, 1080) | 2745 (2269, 3454) |
| 86B | 2641 (2203, 3237) | 3712 (2856, 5574) |

**Supplemental Table 5.** Full table of pharmacokinetic properties of 18A and 18B injected intravenously (5 mg/kg) in CD-1 mice.

|  | 18A | | 18B | |
| --- | --- | --- | --- | --- |
|  | **Plasma** | **Brain** | **Plasma** | **Brain** |
| Terminal t_1/2_ (min) | 27 | 30 | 140 | 73 |
| T_max_ (min) | 5 | 5 | 5 | 5 |
| C_max_ (ng/mL or ng/g) | 1780 | 2968 | 1620 | 1691 |
| Auc_last_ (min*ng/mL or min*ng/g) | 39492 | 63285 | 30688 | 31579 |
| V_ss_ (mL) | 67 |  | 145 |  |
| CL (mL/min) | 3.67 |  | 5.61 |  |
| MRT_inf_ (min) | 17.90 | 16.67 | 25.80 | 17.60 |
